# Frailty, Multimorbidity, and Polypharmacy: Exploratory Analyses of the Effects of Empagliflozin from the EMPA-KIDNEY Trial

**DOI:** 10.2215/CJN.0000000000000498

**Published:** 2024-06-27

**Authors:** Kaitlin J. Mayne, Rebecca J. Sardell, Natalie Staplin, Parminder K. Judge, Doreen Zhu, Emily Sammons, David Z.I. Cherney, Alfred K. Cheung, Aldo P. Maggioni, Masaomi Nangaku, Xavier Rossello, Katherine R. Tuttle, Katsuhito Ihara, Tomoko Iwata, Christoph Wanner, Jonathan Emberson, David Preiss, Martin J. Landray, Colin Baigent, Richard Haynes, William G. Herrington

**Affiliations:** 1 Renal Studies Group, Clinical Trial Service Unit and Epidemiological Studies Unit (CTSU), Nuffield Department of Population Health, University of Oxford, Oxford, United Kingdom; 2 School of Cardiovascular and Metabolic Health, College of Medical and Veterinary Life Sciences, University of Glasgow, Glasgow, United Kingdom; 3 Oxford Kidney Unit, Oxford University Hospitals NHS Foundation Trust, Oxford, United Kingdom; 4 University of Toronto, Toronto, Ontario, Canada; 5 University of Utah, Salt Lake City, Utah; 6 Associazione Nazionale Medici Cardiologi Ospedalieri Research Centre, Florence, Italy; 7 University of Tokyo School of Medicine, Tokyo, Japan; 8 Hospital Universitari Son Espases, Health Research Institute of the Balearic Islands, University of the Balearic Islands, Palma de Mallorca, Spain; 9 University of Washington, Seattle, Washington; 10 Providence Inland Northwest Health, Spokane, Washington; 11 Medicine Division, Nippon Boehringer Ingelheim Co., Ltd., Tokyo, Japan; 12 Boehringer Ingelheim Pharma GmbH & Co KG, Biberach, Germany

## Abstract

**Key Points:**

- Frailty, multimorbidity, and polypharmacy overlap and are associated with higher risk of adverse health outcomes in CKD.
- Empagliflozin was safe, well tolerated, and effectively reduced cardiorenal and hospitalization risk irrespective of these characteristics.
- Absolute benefits appeared greater in the most frail participants in this *post hoc* analysis of EMPA-KIDNEY.

**Background:**

Sodium-glucose cotransporter-2 inhibitors are recommended treatment for adults with CKD, but uncertainty exists regarding their use in patients with frailty and/or multimorbidity, among whom polypharmacy is common. We derived a multivariable logistic regression model to predict hospitalization (reflecting frailty) and assessed empagliflozin's risk–benefit profile in a *post hoc* analysis of the double-blind, placebo-controlled EMPA-KIDNEY trial.

**Methods:**

The EMPA-KIDNEY trial randomized 6609 patients with CKD (eGFR ≥20 to <45 ml/min per 1.73 m^2^, or ≥45 to <90 ml/min per 1.73 m^2^ with urinary albumin-to-creatinine ratio ≥200 mg/g) to receive either empagliflozin 10 mg daily or matching placebo and followed them for 2 years (median). Additional characteristics analyzed in subgroups were multimorbidity, polypharmacy, and health-related quality of life at baseline. Cox regression analyses were performed with subgroups defined by approximate thirds of each variable.

**Results:**

The strongest predictors of hospitalization were *N*-terminal prohormone of brain natriuretic peptide, poor mobility, and diabetes and then eGFR and other comorbidities. Empagliflozin was generally well tolerated independent of predicted risk of hospitalization. In relative terms, allocation to empagliflozin reduced the risk of the primary outcome of kidney disease progression or cardiovascular death by 28% (hazard ratio, 0.72; 95% confidence interval, 0.64 to 0.82) and all-cause hospitalization by 14% (hazard ratio, 0.86; 95% confidence interval, 0.78 to 0.95), with broadly consistent effects across subgroups of predicted risk of hospitalization, multimorbidity, polypharmacy, or health-related quality of life. In absolute terms, the estimated benefits of empagliflozin were greater in those at highest predicted risk of hospitalization (reflecting frailty) and outweighed potential serious harms.

**Conclusions:**

These findings support the use of sodium-glucose cotransporter-2 inhibitors in CKD, irrespective of frailty, multimorbidity, or polypharmacy.

**Clinical Trial registration number:**

NCT03594110.

## Visual Abstract

**Figure:**
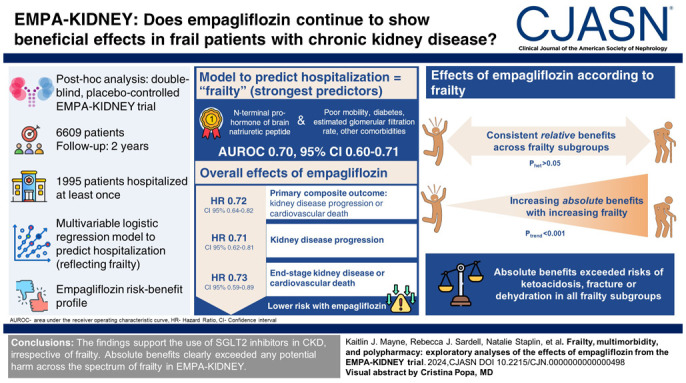

## Introduction

Sodium-glucose cotransporter-2 (SGLT2) inhibitors slow kidney disease progression and reduce cardiovascular risk in patients with CKD.^1,2^ These effects are broadly consistent across different subtypes of patient, including individuals with and without diabetes, and across the spectrum of eGFR studied,^2^ irrespective of kidney disease etiology.^3^ Uncertainty appears to exist surrounding the risk–benefit profile of disease-modifying drugs in older patients and particularly those with frailty, multimorbidity, and/or polypharmacy which are increasingly common in clinical practice^4^ but less well represented in clinical trials.^5^ Recent UK SGLT2 inhibitor clinical practice guidelines recommend “an approach to care that takes account of frailty and multimorbidity… (and) consideration of the balance of disease and treatment burden.”^6^

Frailty, multimorbidity, and polypharmacy are overlapping concepts.^7,8^ Frailty is a syndrome reflecting a state of increased vulnerability to stressors (such as acute illness) because of decline in physiological reserve although there is no internationally accepted definition.^9^ The relationship between frailty and CKD is bidirectional and frailty occurs more commonly in CKD relative to the general population,^10^ particularly as CKD progresses.^11^ Frailty is associated with increased burden of long-term conditions (*i.e*., multimorbidity)^7^ and associated polypharmacy.^8^ Multimorbidity is typically defined as the presence of two or more long-term conditions,^12^ and polypharmacy is generally defined as the regular prescription of five or more drugs.^8^ Frailty, multimorbidity, and polypharmacy may confer an increased absolute risk of drug-related adverse effects.^6,13,14^ Frailty and multimorbidity also confer poor prognosis with greater risks of death, hospitalization, and progression to kidney failure compared with nonfrail adults with CKD.^11,15,16^ Therefore, frailty, multimorbidity, and polypharmacy may all be markers of higher absolute risk, and patients with such characteristics may conversely experience larger absolute benefits of SGLT2 inhibition relative to individuals without burden of frailty, multimorbidity, or polypharmacy.

There are several different tools applied to quantify frailty in clinical practice and research.^17,18^ Approaches in general populations include the Fried frailty phenotype,^7^ the Clinical Frailty Scale,^19^ and the Rockwood Frailty Index.^20^ The Rockwood Frailty Index was developed in community-dwelling adults age older than 70 years in the United States and applies weights to each comorbidity,^20^ which may not be generalizable across diseased populations. Considering this limitation, we developed a bespoke approach for the analysis of EMPA-KIDNEY data on the basis of the established association of clinical frailty with hospitalization,^15,21^ which was a key secondary outcome in EMPA-KIDNEY.^1^ Analyses assessing how the relative and net absolute effects of SGLT2 inhibitors might vary by indicators of frailty, multimorbidity, and polypharmacy in a CKD population have not previously been reported and would help guide practical implementation of current SGLT2 inhibitor guidelines. We used predicted risk of hospitalization at baseline as a surrogate for clinical frailty to quantify how the absolute risk–benefit profile of SGLT2 inhibition varies, as well as separately assessing the impact of multimorbidity, polypharmacy, and health-related quality of life (HRQoL) in this *post hoc* analysis of the EMPA-KIDNEY trial.

## Methods

The full methods of the EMPA-KIDNEY trial and the main results have been reported elsewhere (www.ClinicalTrials.gov number, NCT03594110).^1^ In brief, patients with CKD at risk of progression were identified on the basis of historical and screening local laboratory measurements of an eGFR ≥20 but <45 ml/min per 1.73 m^2^ or an eGFR ≥45 but <90 ml/min per 1.73 m^2^ with a urinary albumin-to-creatinine ratio (UACR) ≥200 mg/g. All participants provided written informed consent. Regulatory authorities, as well as ethics committees in each region, approved the trial.

### Definitions of Frailty, Multimorbidity, Polypharmacy, and HRQoL

In this *post hoc* analysis, baseline predicted risk of hospitalization during follow-up was used as the primary frailty indicator on the basis of the established association with frailty^15,21^ and to maximize the number of events available for analysis (relative to mortality). A wide range of potential predictor variables measured at baseline were considered (Supplemental Table 1). These were then all entered into logistic regression models with first observed hospitalization during follow-up acting as the response variable to select the key independent predictors (see Supplemental Methods) and to estimate each participant's predicted risk of hospitalization (reflecting frailty).

Separate to analyses by predicted risk of hospitalization, we analyzed multimorbidity, polypharmacy, and HRQoL at baseline. Multimorbidity was established by the presence or absence of eight self-reported conditions (Supplemental Methods). Polypharmacy was derived from the number of concomitant medications recorded at the randomization visit and HRQoL was assessed using the EuroQoL EQ-5D-5L tool^22–24^ (Supplemental Methods).

### Outcomes

The same prespecified efficacy and safety outcomes which have previously been reported for the overall trial population^1^ were assessed in these *post hoc* analyses testing the impact of frailty, multimorbidity, and polypharmacy. The primary composite outcome was the time to first occurrence of progression of kidney disease (defined as ESKD [the initiation of maintenance dialysis or receipt of a kidney transplant], a sustained decrease in the eGFR to <10 ml/min per 1.73 m^2^, a sustained decrease from baseline in the eGFR of at least 40%, or death from kidney failure) or death from cardiovascular causes. The prespecified key secondary outcomes were a composite of hospitalization for heart failure or death from cardiovascular causes, hospitalization for any cause (including the first and any subsequent hospitalizations), and death from any cause. Other secondary outcomes were progression of kidney disease, death from cardiovascular causes, and a composite of ESKD or death from cardiovascular causes. Safety outcomes are defined in the Supplemental Methods.

### Statistical Analysis

Full details of prediction models for first hospitalization are provided in the Supplemental Methods. For time to first event outcomes, the effects of allocation to empagliflozin versus placebo were assessed using prespecified Cox regression models adjusted for age, sex, region, eGFR, UACR, and diabetes status. Total hospitalizations were analyzed using joint frailty models as previously described.^1^ Effects on weight and BP were analyzed using a prespecified mixed model repeated measures approach.^1^ Evidence of any effect modification was assessed using standard tests for heterogeneity (for relative effects) or trend (for estimated absolute effects) across the frailty indicator subgroups (see Supplemental Methods for categorizations). Absolute events avoided per 1000 patients treated with empagliflozin per year (SEM) were estimated by applying hazard ratios (HRs) or their 95% confidence intervals (CIs) to the event rate per 1000 patient-years in the placebo group and using the overall HR (or 95% CI) if no strong evidence of heterogeneity in relative effects of treatment was identified or the subgroup-specific HR (or 95% CI) if there was significant heterogeneity (*P* < 0.01).^2^ Analyses were performed using R Studio version 4.2.2 (RStudio: Integrated Development for R. RStudio, PBC, Boston, MA) and SAS version 9.4 (SAS Institute, Cary, NC).

## Results

### Predictors of Frailty Defined as Risk of Hospitalization

**Figure 1:**
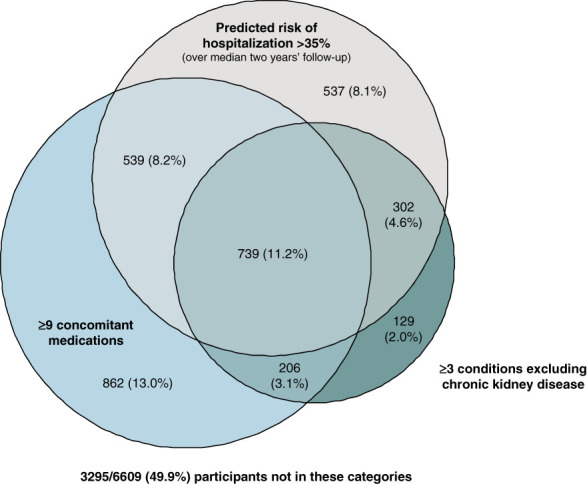
**Number of participants in the top thirds of predicted risk of hospitalization (>35%), multimorbidity (≥3 conditions excluding CKD) and polypharmacy (≥9 concomitant medications) showing degrees of overlap.** Figure presents numbers of participants (%) in the top approximate third of each category applied in subgroup analyses (Figure 3 and Supplemental Tables 7–14). An alternative presentation showing overlap between the highest level of frailty defined in EMPA-KIDNEY (predicted risk of hospitalization >45%) and conventional definitions of multimorbidity (≥2 conditions) and polypharmacy (≥5 medications) is shown in Supplemental Figure 3.

The median (Q1–Q3) follow-up of 6609 randomized participants was 2.0 years (1.5–2.4), during which time 1995 participants were hospitalized at least once (960 in the empagliflozin group and 1035 in the placebo group). The strongest predictors of hospitalization were *N*-terminal prohormone of brain natriuretic peptide (baseline median [Q1–Q3] 160 ng/L [69–419]), poor mobility (based on EQ-5D-5L), and the presence of diabetes (Table 1 and Supplemental Table 2). The final model which additionally included eGFR and other comorbidities (Supplemental Table 3) adequately predicted risk of hospitalization (area under the receiver operating characteristic curve 0.70 [95% CI, 0.69 to 0.71]) and separately death from any cause (Supplemental Figure 1) with acceptable calibration. The median (Q1–Q3) predicted risk of hospitalization was 27% (18–40). Risk of hospitalization was positively correlated with multimorbidity and polypharmacy and inversely with HRQoL (Supplemental Figure 2), and consequently, there was considerable overlap between these subgroups (Figure 1).

**Table 1:** Multivariable logistic regression model used to derive predicted risk of hospitalization

Variable	Participants, *No.* (%)^a^	Hospitalized during Follow-Up, *No.* (%)^a^	OR (95% CI)	*P* Value^b^
Age, per 10 yr increase	—	—	1.09 (1.04 to 1.15)	0.001
Female sex	2192 (33)	615 (28)	0.84 (0.74 to 0.95)	0.004
**Region**				<0.001
Europe	2648 (40)	909 (34)	Ref	
North America	1717 (26)	492 (29)	0.67 (0.58 to 0.77)	
China and Malaysia	1632 (25)	424 (26)	1.14 (0.97 to 1.33)	
Japan	612 (9)	170 (28)	1.20 (0.97 to 1.48)	
Ln NT-proBNP, ng/L	—	—	1.26 (1.20 to 1.33)	<0.001
**Mobility**				<0.001
No problems	4411 (67)	1052 (24)	Ref	
Slight problems	1141 (17)	435 (38)	1.41 (1.21 to 1.64)	
Moderate problems	750 (11)	344 (46)	1.69 (1.41 to 2.02)	
Severe problems	282 (4)	149 (53)	1.93 (1.48 to 2.53)	
Unable to walk about	25 (0.4)	15 (60)	2.59 (1.11 to 6.07)	
**Diabetes**				<0.001
No diabetes	3569 (54)	851 (24)	Ref	
Diabetes without retinopathy	2375 (36)	853 (36)	1.27 (1.12 to 1.44)	
Diabetes with retinopathy	665 (10)	291 (44)	1.62 (1.34 to 1.96)	
Peripheral neuropathy^c^	1316 (20)	557 (42)	1.34 (1.16 to 1.55)	<0.001
Heart failure^c^	658 (10)	333 (51)	1.30 (1.08 to 1.57)	0.006
eGFR, per 10 ml/min per 1.73 m^2^ increase^d^	—	—	0.71 (0.60 to 0.84)	<0.001
Ischemic heart disease^c^	1095 (17)	494 (45)	1.30 (1.12 to 1.51)	0.001
Self-reported ankle swelling^c^	1516 (23)	611 (40)	1.21 (1.06 to 1.38)	0.005

First occurrence of all-cause hospitalization was the response variable. All potential predictor variables assessed are reported in Supplemental Table 1 and were added using a forward stepwise approach based on significance in univariable models (Supplemental Table 2). Age, sex, and region were forced to remain in the model. CI, confidence interval; NT-proBNP, N-terminal pro B-type natriuretic peptide; OR, odds ratio.

a Relevant for categorical variables only.

b Wald test *P* value for continuous and binary outcomes; *P* value from the likelihood ratio test comparing full model with and without the additional variable for categorical variables.

c Effect estimate for presence versus absence of.

d Effect estimate for linear eGFR term, quadratic term also included in the model because of nonlinearity.

### Baseline Characteristics

In the description of results, the use of the term frailty refers to predicted risk of hospitalization during follow-up. Those with the highest levels of frailty had lower levels of albuminuria (*P* < 0.001) but greater 5-year risk of kidney failure (based on the four-variable Kidney Failure Risk Equation; *P* < 0.001) owing to older age and lower eGFR (as would be expected based on the model; Tables 1 and 2; Supplemental Table 4). The 5-year kidney failure risk was 14% (95% CI, 5 to 37) versus 6% (95% CI, 2 to 19) in those with the highest versus lowest levels of frailty. Participants with the highest level of frailty were also more likely to report cardiovascular disease and had higher body mass index (Table 2).

**Table 2:** Characteristics of participants at recruitment by predicted risk of hospitalization

Characteristics	Predicted Risk of Hospitalization during Follow-Up (Median 2 yr)				
	≤20% (*N*=1988)	>20% to ≤35% (*N*=2504)	>35% to ≤45% (*N*=968)	>45% (*N*=1149)	*P* Value
**Demographics**					
Age at randomization (yr)					<0.001
*Mean (SD)*	52.8 (13.8)	65.6 (11.5)	71.4 (9.0)	72.6 (8.8)	
Sex					<0.001
*Female*	745 (38)	860 (34)	294 (30)	293 (26)	
Country					<0.001
*The United Kingdom*	311 (16)	429 (17)	159 (16)	234 (20)	
*Germany*	210 (11)	415 (17)	220 (23)	424 (37)	
*Italy*	72 (4)	95 (4)	40 (4)	39 (3)	
*The United States*	384 (19)	486 (19)	201 (21)	158 (14)	
*Canada*	149 (8)	195 (8)	71 (7)	73 (6)	
*Malaysia*	167 (8)	261 (10)	120 (12)	98 (9)	
*China*	503 (25)	346 (14)	81 (8)	56 (5)	
*Japan*	192 (10)	277 (11)	76 (8)	67 (6)	
**Prior disease**					
Prior diabetes^a^					<0.001
*Yes*	329 (17)	1168 (47)	645 (67)	898 (78)	
Diabetes without retinopathy	308 (16)	980 (39)	478 (49)	609 (53)	
Diabetes with retinopathy	21 (1)	188 (8)	167 (17)	289 (25)	
*No*	1659 (83)	1336 (53)	323 (33)	251 (22)	
History of cardiovascular disease^b^					<0.001
*Yes*	101 (5)	503 (20)	395 (41)	766 (67)	
*No*	1887 (95)	2001 (80)	573 (59)	383 (33)	
No. of comorbid conditions (excluding CKD), median (Q1–Q3)	0 (0–1)	1 (0–2)	2 (1–3)	3 (2–4)	<0.001
**Clinical measurements**					
BP (mm Hg)					
*Mean systolic (SD)*	132 (15)	138 (18)	140 (19)	138 (20)	<0.001
*Mean diastolic (SD)*	82 (11)	79 (12)	75 (11)	73 (12)	<0.001
Body mass index (kg/m^2^)					<0.001
*Mean (SD)*	28.3 (6.3)	29.4 (6.5)	30.8 (7.0)	32.1 (7.1)	
**Laboratory measurements**					
eGFR (ml/min per 1.73 m^2^)					<0.001
*Mean (SD)*	45.1 (15.6)	36.2 (13.7)	33.1 (11.2)	30.0 (9.3)	
UACR (mg/g)					<0.001
*Geometric mean (95% CI)*	299 (277 to 323)	210 (194 to 227)	177 (154 to 202)	183 (162 to 206)	
*Median (Q1–Q3)*	440 (133–1056)	314 (43–1062)	220 (29–1060)	193 (34–1118)	
**Concomitant medication use**					
RAS inhibitor	1791 (90)	2100 (84)	790 (82)	947 (82)	<0.001
Any diuretic therapy	507 (26)	925 (37)	548 (57)	835 (73)	<0.001
Lipid-lowering therapy	968 (49)	1699 (68)	745 (77)	966 (84)	<0.001
Count of concomitant medications at randomization, median (Q1–Q3)	5 (3–7)	7 (5–9)	9 (6–11)	10 (8–13)	<0.001
5-yr risk of kidney failure (KFRE, %), median (Q1–Q3)	6 (2–19)	10 (3–32)	11 (4–34)	14 (5–37)	<0.001
**Cause of kidney disease**					<0.001
Diabetic kidney disease	203 (10)	773 (31)	460 (48)	621 (54)	
Hypertension/renovascular	348 (18)	627 (25)	233 (24)	237 (21)	
Glomerular	987 (50)	545 (22)	81 (8)	56 (5)	
Other/unknown	450 (23)	559 (22)	194 (20)	235 (20)	
**HRQoL**					
Visual analog scale rating, median (Q1–Q3)	85.0 (80.0–90.0)	80.0 (70.0–90.0)	80.0 (68.8–85.0)	70.0 (50.0–80.0)	<0.001
EQ-5D index value, median (Q1–Q3)	0.99 (0.87–0.99)	0.90 (0.80–0.99)	0.84 (0.72–0.99)	0.72 (0.60–0.87)	<0.001

Figures are *No.* (%) or mean (SD) or median (Q1–Q3). Predicted risk of hospitalization was derived from multivariable logistic regression models adjusted for age, sex, and region assessing the association of all potential predictor variables with recorded hospitalization (first event; see Supplemental Methods). CI, confidence interval; HRQoL, health-related quality of life; KFRE, kidney failure risk equation; RAS, renin-angiotensin system; UACR, urinary albumin-to-creatinine ratio.

a Prior diabetes defined as participant-reported history of diabetes of any type, use of glucose-lowering medication, or baseline hemoglobin A1c ≥48 mmol/mol at randomization visit.

b Defined as self-reported history of myocardial infarction, heart failure, stroke, transient ischemic attack, or peripheral arterial disease. *P* values are from Chi squared tests for categorical variables; one-way ANOVA for normally distributed and Kruskal–Wallis tests for non-normally distributed continuous variables, respectively.

The median (Q1–Q3) number of comorbid conditions (excluding CKD) before randomization was 1 (0–2); range 0–7 (Supplemental Table 5). The median (Q1–Q3) number of concomitant medications recorded at randomization was 7 (5–10), range 0–36 (Table 2). At randomization, prescription of five or more concomitant medications (*i.e*., polypharmacy) was present in 76% (5044/6609) of participants, and at least one condition in addition to CKD (*i.e*., multimorbidity) was present in 71% of participants (4675/6609). Of 5635 participants who fulfilled these definitions of either polypharmacy or multimorbidity, 72% (4084/5635) were included in both groups (Supplemental Figure 3). The median (Q1–Q3) indexed EQ-5D value was 0.891 (0.773–0.987), and the median (Q1–Q3) self-rated health score (visual analog scale) was 80 (70–90) with scores ranging from 0 to 100.

### Adherence to Study Treatment

Adherence to study treatment was reasonably high in all subgroups but did inversely correlate with frailty at baseline. At 12 months of follow-up (the approximate midpoint of the trial), the proportion of surviving participants reportedly taking most (>80%) of their study treatment was highest in patients in the lowest frailty category (based on risk of hospitalization; 1830/1982, 92%) and lowest in those with the highest level of frailty (938/1090, 86%). Participants with greater levels of frailty (with similar patterns observed for polypharmacy) were more likely to discontinue study treatment (by the end of follow-up) in both the empagliflozin and placebo groups, but reasons for discontinuation were rarely attributed to serious adverse events (Supplemental Table 6).

### Relative Effects on the Primary Outcome and Kidney Disease Progression

**Figure 2:**
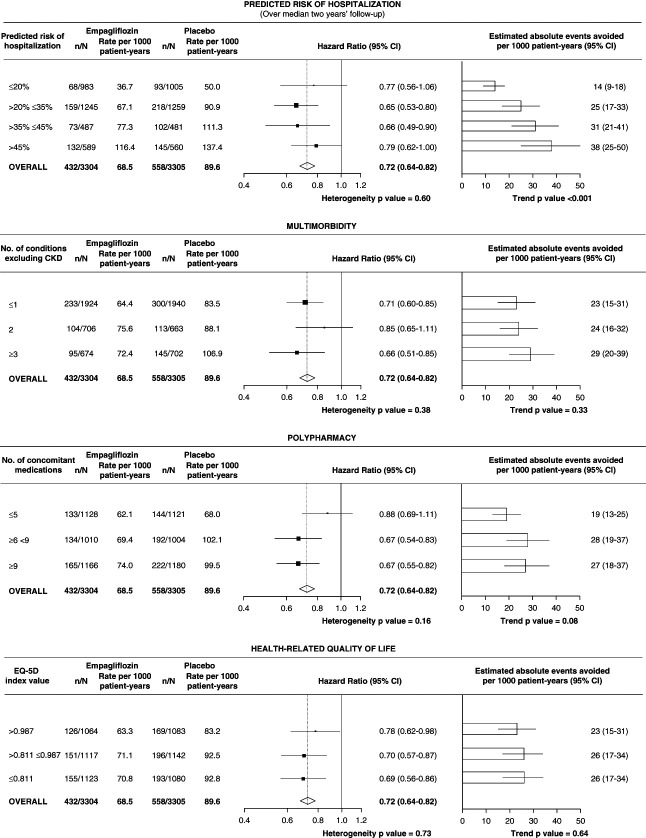
**Effects of empagliflozin on the primary outcome of kidney disease progression or cardiovascular death by frailty, multimorbidity, polypharmacy, and HRQoL.** Because of the absence of any evidence of effect modification by the presented characteristics, absolute events avoided per 1000 patients treated with empagliflozin per 1 year (95% CI) were estimated by applying the overall HR (or 95% CI) to the event rate per 1000 patient-years in the placebo group. CI, confidence interval; HR, hazard ratio; HRQoL, health-related quality of life.

Overall, versus placebo, empagliflozin reduced the risk of the primary composite outcome of kidney disease progression or cardiovascular death by 28% (HR, 0.72; 95% CI, 0.64 to 0.82), with no significant difference in relative effects by baseline level of frailty, multimorbidity, polypharmacy, or HRQoL (*P* for heterogeneity all >0.05, Figure 2, Supplemental Tables 7–10). The majority of the 990 primary outcome events were due to kidney disease progression (888 events), and overall, empagliflozin reduced the risk of this secondary outcome by 29% (HR, 0.71; 95% CI, 0.62 to 0.81) and the risk of a composite of ESKD or cardiovascular death by 27% (HR, 0.73; 95% CI, 0.59 to 0.89), with no significant heterogeneity between subgroups (for frailty, multimorbidity, polypharmacy, or HRQoL) for either outcome (Supplemental Tables 7–10).

### Relative Effects on Key Secondary Outcomes

In total, 1611 hospitalizations occurred among 960 patients in the empagliflozin group, and 1895 hospitalizations occurred among 1035 patients in the placebo group during follow-up. Overall, there was a 14% reduction in total all-cause hospitalizations in participants allocated to empagliflozin versus placebo (HR, 0.86; 95% CI, 0.78 to 0.95), which was not clearly driven by a single cause of hospitalization (see previous reports).^1^ On a relative scale, analyses by baseline measures of frailty showed no strong evidence of heterogeneity by baseline levels of frailty, multimorbidity, or polypharmacy (Supplemental Figure 4). Considering the number of tests conducted, there was weak evidence of heterogeneity by baseline HRQoL (*P* = 0.01, Supplemental Figure 4). These relative effects of empagliflozin on risk of all-cause hospitalization were also similar in those with and without diabetes and were unmodified by baseline eGFR or baseline UACR (Supplemental Figure 5). No significant effect was observed overall on the composite outcome of hospitalization for heart failure or death from cardiovascular causes (HR, 0.84; 95% CI, 0.67 to 1.07) or death from any cause (HR, 0.87; 95% CI, 0.70 to 1.08), with no evidence of significant heterogeneity between subgroups for either outcome (Supplemental Tables 7–10).

### Relative Effects on Safety Outcomes and Physical Measurements

Safety outcomes were more common in participants with indicators of higher frailty, but there was no evidence that these were increased by empagliflozin compared with placebo at any level of frailty. In particular, allocation to empagliflozin relative to placebo did not result in any excess of symptomatic dehydration or fractures (Supplemental Tables 11–14). Nor did the reported effects on body weight or BP vary by baseline frailty (Supplemental Figure 6).

### Absolute Benefits and Risks

**Figure 3:**
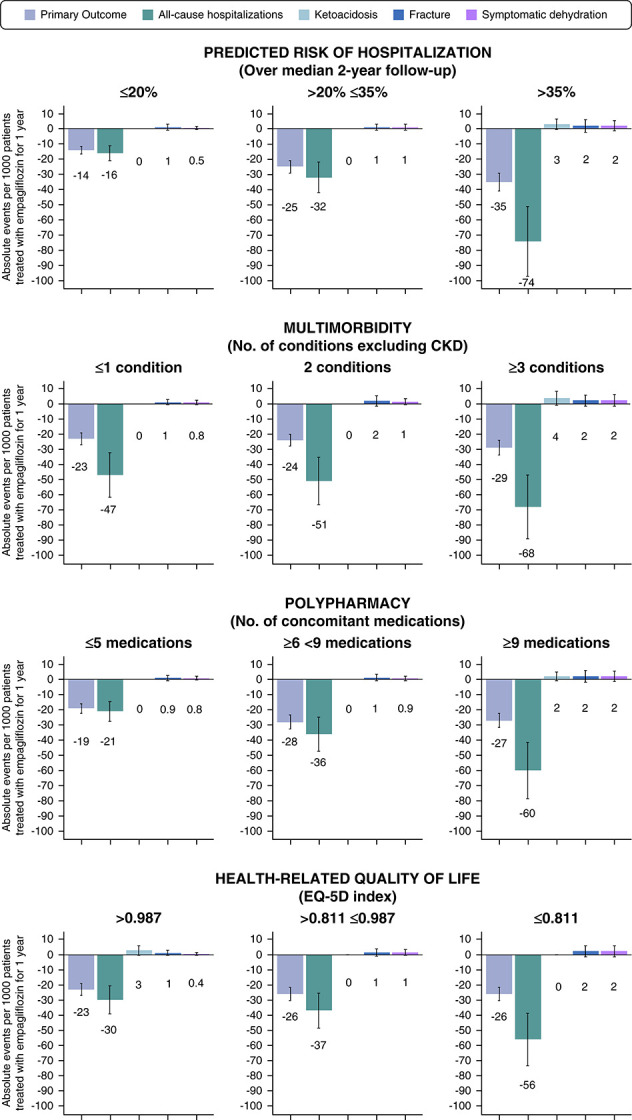
**Absolute benefits and harms of empagliflozin per 1000 patient-years by frailty, multimorbidity, polypharmacy, and HRQoL.** Absolute events avoided per 1000 patients treated with empagliflozin per 1 year (and SEM represented by error bars) were estimated by applying the overall HR or 95% CI to the event rate per 1000 patient-years in the placebo group. If subgroup-specific HRs (or CIs) were used to estimate absolute effects on all-cause hospitalization by HRQoL, based on *P* for heterogeneity=0.01 for relative effects; the numbers of estimated absolute events avoided would be 1, 87, and 39, respectively (rather than 30, 37, and 56 as plotted). Prespecified analyses of all-cause hospitalizations include first and recurrent events; all other events are time-to-first event analyses.

There was evidence of larger estimated absolute benefits on the primary outcome of kidney disease progression or cardiovascular death and on all-cause hospitalizations in participants in the top third of frailty (Figures 2 and 3 and Supplemental Figure 4) compared with those with lesser degrees of frailty. Per 1000 participants treated, it was estimated that occurrences of kidney disease progression or cardiovascular death (*i.e*., primary outcomes) were avoided by empagliflozin in 35, 25, and 14 participants per year in the top, middle, and lowest thirds of frailty, respectively. The total number of hospitalizations avoided annually by empagliflozin treatment was 74, 32, and 16 in the top, middle, and lowest thirds of frailty, respectively (Figure 3). Low absolute excess risk of safety outcomes meant these estimated absolute benefits of empagliflozin substantially outweighed the potential serious harms in the studied population (Figure 3).

## Discussion

The aims of these *post hoc* exploratory analyses of EMPA-KIDNEY data were to characterize the risk–benefit profile of SGLT2 inhibition in CKD by differing levels of frailty (indicated by predicted risk of hospitalization), multimorbidity, and polypharmacy. EMPA-KIDNEY studied a broader range of patients at risk of CKD progression than the other large SGLT2 inhibitor trials in CKD, including large numbers of participants with low levels of albuminuria and without diabetes.^1,25^ Empagliflozin clearly reduced the risk of kidney disease progression or cardiovascular death with no evidence that the relative benefits were modified by levels of frailty, multimorbidity, or polypharmacy. Furthermore, larger absolute benefits were observed in participants with the highest predicted risk of hospitalization (reflecting frailty). These absolute benefits were achieved safely and clearly outweigh the potential harms of SGLT2 inhibition in the studied population. Serious AKI occurred much more commonly in patients with the highest levels of frailty, multimorbidity, or polypharmacy. Other data have shown that SGLT2 inhibitors reduce the risk of AKI meaning any effect of empagliflozin on AKI would mean larger absolute benefits among these types of patient. These findings should encourage use of indicated SGLT2 inhibitor treatment in adults with CKD, irrespective of frailty, multimorbidity, polypharmacy, or HRQoL. Although patient preference is important, SGLT2 inhibitor discontinuation simply to reduce tablet burden is not risk free and would be expected to increase the likelihood of several important and avoidable adverse health consequences.^26^

Our findings are consistent with reports from the DAPA-HF^27^ and DELIVER^28^ trials which used the general population-derived Rockwood Frailty Index approach in populations with heart failure. In these analyses, the beneficial effects of dapagliflozin in reducing the risk of the composite primary outcome of worsening heart failure or cardiovascular death were apparent across the spectrum of frailty represented in the two trials, with larger absolute benefits in the participants with the highest levels of frailty.^27,28^ The Rockwood Frailty Index approach was also applied in the DAPA-CKD trial which recruited patients with proteinuric CKD, about two thirds of whom had diabetes.^29^ The absolute benefits of dapagliflozin versus placebo were the greatest among the most frail with respect to cardiovascular and mortality outcomes although effects on the primary composite kidney outcome did not differ on either a relative or absolute scale when participants were divided according to their Rockwood Frailty Index score.^30^ Importantly, in each of these trials, and similarly in EMPA-KIDNEY, SGLT2 inhibitors were well tolerated even at high levels of frailty.^27,28,30^ We observed that patients at greater predicted risk of hospitalization were more likely to discontinue study treatment, whether allocated empagliflozin or placebo; yet, there was no excess of discontinuation of empagliflozin relative to placebo irrespective of predicted risk of hospitalization. Because levels of discontinuation of study treatment were relatively low across all subgroups, the 28% relative risk reduction for the primary outcome reflects only a slight underestimate of the full effect in all participants, irrespective of level of frailty.

It has been suggested SGLT2 inhibitors may cause a degree of breakdown of skeletal muscle and loss of lean tissue mass,^31,32^ because of upregulated gluconeogenesis, causing lipolysis and proteolysis of adipose and skeletal muscle tissue, respectively.^33^ This may be a particular concern in underweight or malnourished patients; however, because glycosuric effects and body weight reduction are attenuated in those with lower eGFR, such concerns may be less relevant in patients with CKD. Indeed, other studies have not shown changes in muscle mass with SGLT2 inhibition, regardless of eGFR.^34^ An EMPA-KIDNEY bioimpedance substudy conducted in a subset of approximately 10% of the trial population corroborated these findings because there was no significant effect of empagliflozin versus placebo on either lean tissue or fat mass, with weight loss appearing to exclusively reflect reductions in total body water volume.^35^ Furthermore, the effects of empagliflozin on the primary outcome of kidney disease progression or cardiovascular death in the EMPA-KIDNEY population of 6609 individuals were similar across the spectrum of studied body mass index.^1^

EMPA-KIDNEY has demonstrated clear benefits of SGLT2 inhibition on kidney disease progression in a wide range of patients with CKD at risk of progression.^1^ These analyses benefit from the trial's sample size, median 2-year follow-up duration, and randomized double-blind design. However, some limitations remain. First, because clinical trials typically recruit healthier patients, levels of frailty in EMPA-KIDNEY may not be representative of the general CKD population. Second, our assessment of frailty did not use an externally validated frailty assessment tool because these largely depend on clinical assessments not conducted in large streamlined randomized trials like EMPA-KIDNEY. We chose not to use existing indices like the Rockwood Frailty Index which weight characteristics differentially, and because the approach was developed in a general population, this approach to weighting may not be generalizable into diseased populations (*e.g*., CKD). We, therefore, developed a bespoke assessment of frailty using predicted risk of hospitalization as a marker of clinical frailty; accepting our model may not be broadly generalizable but is a scientifically robust approach for the studied population. Our model also performed well for prediction of death. Finally, geographical variation in hospitalization patterns might also limit the generalizability of the absolute benefits for this outcome, but do not affect the overall pattern of increasing net absolute benefits among those with higher levels of frailty, multimorbidity, or polypharmacy.

In conclusion, empagliflozin was safe, well tolerated, and effectively lowered the risk of progression of kidney disease or cardiovascular death (the primary outcome) and all-cause hospitalization in a broad range of patients studied in EMPA-KIDNEY, irrespective of indicators of frailty, multimorbidity, or polypharmacy. The absolute benefits of empagliflozin were in fact greater in patients with the highest levels of frailty (indicated by predicted risk of hospitalization). Clinical guidelines should encourage evidence-based prescribing of SGLT2 inhibitors in individuals with CKD irrespective of frailty, multimorbidity, or polypharmacy and emphasize that such patients may stand to gain most from treatment.

## Supplementary Material

## Data Availability

Partial restrictions to the data and/or materials apply. The complete deidentified patient data set used for presented analyses will be available in due course, and the application system to apply to use data will open 6 months after publication. Departmental policy details can be found here: https://www.ndph.ox.ac.uk/data-access. In adherence with the Boehringer Ingelheim Policy on Transparency and Publication of Clinical Study Data, scientific and medical researchers can request access to clinical study data, typically, 1 year after the approval has been granted by major Regulatory Authorities or after termination of the development program. Researchers should use the https://vivli.org/link to request access to study data and visit https://www.mystudywindow.com/msw/datasharing for further information.

## Supplemental Material

Supplemental Summary 1. Members of the EMPA-KIDNEY Collaborative Group.

Supplemental Methods

Supplemental Table 1. Variables assessed as potential predictors of hospitalization.

Supplemental Table 2. Univariable associations with hospitalization (logistic regression).

Supplemental Table 3. Incremental impact of each variable in the final multivariable logistic regression model to predict hospitalization.

Supplemental Table 4. Other characteristics of participants at recruitment by predicted risk of hospitalization.

Supplemental Table 5. Composition of multimorbidity subgroups.

Supplemental Table 6. Number of participants who had discontinued randomized treatment at the end of follow-up and reasons for discontinuation according to (i) predicted risk of hospitalization and (ii) number of concomitant medications.

Supplemental Table 7. Primary and secondary outcomes by predicted risk of hospitalization.

Supplemental Table 8. Primary and secondary outcomes by multimorbidity.

Supplemental Table 9. Primary and secondary outcomes by concomitant medication count.

Supplemental Table 10. Primary and secondary outcomes by health-related quality of life (EQ-5D index value).

Supplemental Table 11. Safety outcomes by predicted risk of hospitalization.

Supplemental Table 12. Safety outcomes by multimorbidity.

Supplemental Table 13. Safety outcomes by concomitant medication count.

Supplemental Table 14. Safety outcomes by health-related quality of life (EQ-5D index value).

Supplemental Figure 1. Performance of the final multivariable logistic regression model in predicting hospitalization and all-cause death.

Supplemental Figure 2. Associations between predicted risk of hospitalization and multimorbidity, polypharmacy, and health-related quality of life.

Supplemental Figure 3. Number of participants in the highest level of frailty (defined as predicted risk of hospitalization >45%) in EMPA-KIDNEY showing overlap with conventional definitions of multimorbidity and polypharmacy (which differ from Figure 1).

Supplemental Figure 4. Effects of empagliflozin versus placebo on recurrent all-cause hospitalization by frailty (based on predicted risk of hospitalization), multimorbidity, polypharmacy, and health-related quality of life.

Supplemental Figure 5. Effects of empagliflozin versus placebo on recurrent all-cause hospitalization by key prespecified subgroups.

Supplemental Figure 6. Effects of empagliflozin versus placebo on weight and BP by predicted risk of hospitalization.
