# Supplementary material for: Frailty, Multimorbidity, and Polypharmacy: Exploratory Analyses of the Effects of Empagliflozin from the EMPA-KIDNEY Trial
Source: Clin J Am Soc Nephrol. 2024 Jun 27;19(9):1119–29. doi: 10.2215/CJN.0000000000000498 (PMC11390031; doi:10.2215/CJN.0000000000000498)
Supplement: Supplementary file 1 [file cjasn-19-1119-s001.pdf]

ASN American Society of Nephrology | JASN Journal of the American Society of Nephrology | KIDNEY360 Accessing Our World From Every Angle | CJASN Clinical Journal of the American Society of Nephrology

ASN Journal Disclosure Form

As per ASN journal policy, I have disclosed any financial relationships or commitments I have held in the past 36 months as included below. I have listed my Current Employer below to indicate there is a relationship requiring disclosure. If no relationship exists, my Current Employer is not listed.

C. Baigent reports the following:
Employer: University of Oxford; Research Funding: Boehringer Ingelheim; Advisory or Leadership Role: I have served as Chair of the European Society of Cardiology Clinical Practice Guidelines Committee 2020-22; and Other Interests or Relationships: I am a trustee of the UK charity alport-uk, which supports patients and families with Alport Syndrome.

I understand that the information above will be published within the journal article, if accepted, and that failure to comply and/or to accurately and completely report the potential financial conflicts of interest could lead to the following: 1) Prior to publication, article rejection, or 2) Post-publication, sanctions ranging from, but not limited to, issuing a correction, reporting the inaccurate information to the authors' institution, banning authors from submitting work to ASN journals for varying lengths of time, and/or retraction of the published work.

Name: Colin Baigent
Manuscript ID: CJASN-2024-000275R2
Manuscript Title: Frailty, multimorbidity and polypharmacy: exploratory analyses of the effects of empagliflozin from the EMPA-KIDNEY trial
Date of Completion: May 29, 2024
Disclosure Updated Date: May 14, 2024

ASN American Society of Nephrology | JASN Journal of the American Society of Nephrology | KIDNEY360 Accessing Our World From Every Angle | CJASN Clinical Journal of the American Society of Nephrology

ASN Journal Disclosure Form

As per ASN journal policy, I have disclosed any financial relationships or commitments I have held in the past 36 months as included below. I have listed my Current Employer below to indicate there is a relationship requiring disclosure. If no relationship exists, my Current Employer is not listed.

D. Cherney reports the following:
Employer: Toronto General Hospital; Consultancy: Boehringer Ingelheim-Lilly, Merck, AstraZeneca, Sanofi, Mitsubishi-Tanabe, Abbvie, Janssen, Bayer, Prometic, Lexicon, BMS, Maze, CSL-Behring, Otsuka, Novartis, Yeungene and Novo-Nordisk; Research Funding: Boehringer Ingelheim-Lilly, Merck, Novo Nordisk and AstraZeneca, CSL-BEHRING; Honoraria: Boehringer Ingelheim-Lilly, Merck, AstraZeneca, Sanofi, Mitsubishi-Tanabe, Abbvie, Janssen, Bayer, Prometic, BMS, Maze, CSL-Behring, Otsuka, Novartis, Yeungene and Novo-Nordisk; and Advisory or Leadership Role: Boehringer Ingelheim-Lilly, Merck, AstraZeneca, Lexicon, Janssen, Bayer, BMS, Maze, CSL-Behring, Novartis, Novo-Nordisk.

I understand that the information above will be published within the journal article, if accepted, and that failure to comply and/or to accurately and completely report the potential financial conflicts of interest could lead to the following: 1) Prior to publication, article rejection, or 2) Post-publication, sanctions ranging from, but not limited to, issuing a correction, reporting the inaccurate information to the authors' institution, banning authors from submitting work to ASN journals for varying lengths of time, and/or retraction of the published work.

Name: David Cherney
Manuscript ID: CJASN-2024-000275R2
Manuscript Title: EMPA-KIDNEY frailty manuscript
Date of Completion: May 29, 2024
Disclosure Updated Date: January 6, 2024

ASN American Society of Nephrology
JASN Journal of the American Society of Nephrology
KIDNEY360 Accessing Our World From Every Angle
CJASN Clinical Journal of the American Society of Nephrology

ASN Journal Disclosure Form

As per ASN journal policy, I have disclosed any financial relationships or commitments I have held in the past 36 months as included below. I have listed my Current Employer below to indicate there is a relationship requiring disclosure. If no relationship exists, my Current Employer is not listed.

A. Cheung reports the following:
Employer: University of Utah; Consultancy: Boehringer-Ingelheim, CSL Behring, 3D Communications, Alucent, Nova Nordisk.; Ownership Interest: Merck; Patents or Royalties: Uptodate; and Advisory or Leadership Role: KDIGO.

I understand that the information above will be published within the journal article, if accepted, and that failure to comply and/or to accurately and completely report the potential financial conflicts of interest could lead to the following: 1) Prior to publication, article rejection, or 2) Post-publication, sanctions ranging from, but not limited to, issuing a correction, reporting the inaccurate information to the authors' institution, banning authors from submitting work to ASN journals for varying lengths of time, and/or retraction of the published work.

Name: Alfred K. Cheung
Manuscript ID: CJASN-2024-000275R2
Manuscript Title: Frailty, multimorbidity and polypharmacy: exploratory analyses of the effects of empagliflozin from the EMPA-KIDNEY trial
Date of Completion: June 18, 2024
Disclosure Updated Date: June 18, 2024

ASN American Society of Nephrology
JASN Journal of the American Society of Nephrology
KIDNEY360 Accessing Our World From Every Angle
CJASN Clinical Journal of the American Society of Nephrology

ASN Journal Disclosure Form

As per ASN journal policy, I have disclosed any financial relationships or commitments I have held in the past 36 months as included below. I have listed my Current Employer below to indicate there is a relationship requiring disclosure. If no relationship exists, my Current Employer is not listed.

J. Emberson reports the following:
Employer: University of Oxford; and Research Funding: Boehringer Ingleheim.

I understand that the information above will be published within the journal article, if accepted, and that failure to comply and/or to accurately and completely report the potential financial conflicts of interest could lead to the following: 1) Prior to publication, article rejection, or 2) Post-publication, sanctions ranging from, but not limited to, issuing a correction, reporting the inaccurate information to the authors' institution, banning authors from submitting work to ASN journals for varying lengths of time, and/or retraction of the published work.

Name: Jonathan Emberson
Manuscript ID: CJASN-2024-000275R1
Manuscript Title: Frailty, multimorbidity and polypharmacy: exploratory analyses of the effects of empagliflozin in CKD from the EMPA-KIDNEY trial
Date of Completion: April 22, 2024
Disclosure Updated Date: October 9, 2023

ASN American Society of Nephrology | JASN Journal of the American Society of Nephrology | KIDNEY360 Accessing Our World From Every Angle | CJASN Clinical Journal of the American Society of Nephrology

ASN Journal Disclosure Form

As per ASN journal policy, I have disclosed any financial relationships or commitments I have held in the past 36 months as included below. I have listed my Current Employer below to indicate there is a relationship requiring disclosure. If no relationship exists, my Current Employer is not listed.

R. Haynes reports the following:
Employer: University of Oxford

I understand that the information above will be published within the journal article, if accepted, and that failure to comply and/or to accurately and completely report the potential financial conflicts of interest could lead to the following: 1) Prior to publication, article rejection, or 2) Post-publication, sanctions ranging from, but not limited to, issuing a correction, reporting the inaccurate information to the authors' institution, banning authors from submitting work to ASN journals for varying lengths of time, and/or retraction of the published work.

Name: Richard Haynes
Manuscript ID: CJASN-2024-000275
Manuscript Title: Frailty, multimorbidity and polypharmacy: exploratory analyses of the effects of empagliflozin in CKD from the EMPA-KIDNEY trial
Date of Completion: April 22, 2024
Disclosure Updated Date: April 22, 2024

ASN American Society of Nephrology | JASN Journal of the American Society of Nephrology | KIDNEY360 Accessing Our World From Every Angle | CJASN Clinical Journal of the American Society of Nephrology

ASN Journal Disclosure Form

As per ASN journal policy, I have disclosed any financial relationships or commitments I have held in the past 36 months as included below. I have listed my Current Employer below to indicate there is a relationship requiring disclosure. If no relationship exists, my Current Employer is not listed.

W. Herrington reports the following:
Employer: University of Oxford; Research Funding: Boehringer Ingelheim & Eli Lilly to conduct renal trials (including EMPA-KIDNEY); and Advisory or Leadership Role: NDT subject editor; UK Kidney Association, European Society of Cardiology & KDIGO guideline committee roles. UK Renal Trial Network Chair. I decline all honoraria from the pharmaceutical or food industry, except for reasonable travel expenses.

I understand that the information above will be published within the journal article, if accepted, and that failure to comply and/or to accurately and completely report the potential financial conflicts of interest could lead to the following: 1) Prior to publication, article rejection, or 2) Post-publication, sanctions ranging from, but not limited to, issuing a correction, reporting the inaccurate information to the authors' institution, banning authors from submitting work to ASN journals for varying lengths of time, and/or retraction of the published work.

Name: William G. Herrington
Manuscript ID: CJASN-2024-000275R1
Manuscript Title: Frailty, multimorbidity and polypharmacy: exploratory analyses of the effects of empagliflozin in CKD from the EMPA-KIDNEY trial.
Date of Completion: April 18, 2024
Disclosure Updated Date: March 22, 2024

ASN American Society of Nephrology JASN Journal of the American Society of Nephrology KIDNEY360 Accessing Our World From Every Angle CJASN Clinical Journal of the American Society of Nephrology

ASN Journal Disclosure Form

As per ASN journal policy, I have disclosed any financial relationships or commitments I have held in the past 36 months as included below. I have listed my Current Employer below to indicate there is a relationship requiring disclosure. If no relationship exists, my Current Employer is not listed.

K. Ihara reports the following:
Employer: Nippon Boehringer Ingelheim Co., Ltd.

I understand that the information above will be published within the journal article, if accepted, and that failure to comply and/or to accurately and completely report the potential financial conflicts of interest could lead to the following: 1) Prior to publication, article rejection, or 2) Post-publication, sanctions ranging from, but not limited to, issuing a correction, reporting the inaccurate information to the authors' institution, banning authors from submitting work to ASN journals for varying lengths of time, and/or retraction of the published work.

Name: Katsuhito Ihara
Manuscript ID: CJASN-2024-000275R2
Manuscript Title: Frailty, multimorbidity and polypharmacy: exploratory analyses of the effects of empagliflozin from the EMPA-KIDNEY trial
Date of Completion: June 18, 2024
Disclosure Updated Date: May 20, 2024

ASN American Society of Nephrology | JASN Journal of the American Society of Nephrology | KIDNEY360 Accessing Our World From Every Angle | CJASN Clinical Journal of the American Society of Nephrology

ASN Journal Disclosure Form

As per ASN journal policy, I have disclosed any financial relationships or commitments I have held in the past 36 months as included below. I have listed my Current Employer below to indicate there is a relationship requiring disclosure. If no relationship exists, my Current Employer is not listed.

T. Iwata reports the following:
Employer: Boehringer Ingelheim GmbH & Co. KG

I understand that the information above will be published within the journal article, if accepted, and that failure to comply and/or to accurately and completely report the potential financial conflicts of interest could lead to the following: 1) Prior to publication, article rejection, or 2) Post-publication, sanctions ranging from, but not limited to, issuing a correction, reporting the inaccurate information to the authors' institution, banning authors from submitting work to ASN journals for varying lengths of time, and/or retraction of the published work.

Name: Tomoko Iwata
Manuscript ID: CJASN-2024-000275R2
Manuscript Title: Frailty, multimorbidity and polypharmacy: exploratory analyses of the effects of empagliflozin from the EMPA-KIDNEY trial
Date of Completion: June 4, 2024
Disclosure Updated Date: June 4, 2024

ASN American Society of Nephrology | JASN Journal of the American Society of Nephrology | KIDNEY360 Accessing Our World From Every Angle | CJASN Clinical Journal of the American Society of Nephrology

ASN Journal Disclosure Form

As per ASN journal policy, I have disclosed any financial relationships or commitments I have held in the past 36 months as included below. I have listed my Current Employer below to indicate there is a relationship requiring disclosure. If no relationship exists, my Current Employer is not listed.

P. Judge reports the following:
Employer: University of Oxford; and Research Funding: The UK HARP-III trial was funded by a grant to the University of Oxford from Novartis.; The EMPA-KIDNEY trials was funded by a grant to the University of Oxford from Boehringer Ingelheim & Eli Lily; The EASi-KIDNEY trial is funded by a grant to the University of Oxford from Boehringer Ingelheim.

I understand that the information above will be published within the journal article, if accepted, and that failure to comply and/or to accurately and completely report the potential financial conflicts of interest could lead to the following: 1) Prior to publication, article rejection, or 2) Post-publication, sanctions ranging from, but not limited to, issuing a correction, reporting the inaccurate information to the authors' institution, banning authors from submitting work to ASN journals for varying lengths of time, and/or retraction of the published work.

Name: Parminder K. Judge
Manuscript ID: CJASN-2024-000275R1
Manuscript Title: Frailty, multimorbidity and polypharmacy: exploratory analyses of the effects of empagliflozin in CKD from the EMPA-KIDNEY trial
Date of Completion: April 18, 2024
Disclosure Updated Date: April 18, 2024

ASN American Society of Nephrology JASN Journal of the American Society of Nephrology KIDNEY360 Accessing Our World From Every Angle CJASN Clinical Journal of the American Society of Nephrology

ASN Journal Disclosure Form

As per ASN journal policy, I have disclosed any financial relationships or commitments I have held in the past 36 months as included below. I have listed my Current Employer below to indicate there is a relationship requiring disclosure. If no relationship exists, my Current Employer is not listed.

M. Landray reports the following:
Employer: University of Oxford; and Research Funding: Boehringer Ingelheim; Novartis; Regeneron; Sanofi; Moderna; Apollo Tx; GSK; Vaxxinity.

I understand that the information above will be published within the journal article, if accepted, and that failure to comply and/or to accurately and completely report the potential financial conflicts of interest could lead to the following: 1) Prior to publication, article rejection, or 2) Post-publication, sanctions ranging from, but not limited to, issuing a correction, reporting the inaccurate information to the authors' institution, banning authors from submitting work to ASN journals for varying lengths of time, and/or retraction of the published work.

Name: Martin J. Landray
Manuscript ID: CJASN-2024-000275R1
Manuscript Title: Frailty, multimorbidity and polypharmacy: exploratory analyses of the effects of empagliflozin in CKD from the EMPA-KIDNEY trial
Date of Completion: April 29, 2024
Disclosure Updated Date: April 29, 2024

ASN American Society of Nephrology | JASN Journal of the American Society of Nephrology | KIDNEY360 Accessing Our World From Every Angle | CJASN Clinical Journal of the American Society of Nephrology

ASN Journal Disclosure Form

As per ASN journal policy, I have disclosed any financial relationships or commitments I have held in the past 36 months as included below. I have listed my Current Employer below to indicate there is a relationship requiring disclosure. If no relationship exists, my Current Employer is not listed.

A. Maggioni reports the following:
Employer: Heart Care Foundation; Research Funding: Boehringer Ingelheim; and Advisory or Leadership Role: Bayer, Novartis (DSMB member); AstraZeneca, Novartis and Sanofi (Steering Committee member).

I understand that the information above will be published within the journal article, if accepted, and that failure to comply and/or to accurately and completely report the potential financial conflicts of interest could lead to the following: 1) Prior to publication, article rejection, or 2) Post-publication, sanctions ranging from, but not limited to, issuing a correction, reporting the inaccurate information to the authors' institution, banning authors from submitting work to ASN journals for varying lengths of time, and/or retraction of the published work.

Name: Aldo Pietro Maggioni
Manuscript ID: CJASN-2024-000275R1
Manuscript Title: Frailty, multimorbidity and polypharmacy: exploratory analyses of the effects of empagliflozin in CKD from the EMPA-KIDNEY trial
Date of Completion: April 19, 2024
Disclosure Updated Date: April 19, 2024

ASN American Society of Nephrology | JASN Journal of the American Society of Nephrology | KIDNEY360 Accessing Our World From Every Angle | CJASN Clinical Journal of the American Society of Nephrology

ASN Journal Disclosure Form

As per ASN journal policy, I have disclosed any financial relationships or commitments I have held in the past 36 months as included below. I have listed my Current Employer below to indicate there is a relationship requiring disclosure. If no relationship exists, my Current Employer is not listed.

K. Mayne reports the following:
Research Funding: Boehringer Ingelheim and Eli Lilly - grant to institution to design and conduct EMPA-KIDNEY trial & EASi-KIDNEY trial; MRC-UK - core funding paid to department

I understand that the information above will be published within the journal article, if accepted, and that failure to comply and/or to accurately and completely report the potential financial conflicts of interest could lead to the following: 1) Prior to publication, article rejection, or 2) Post-publication, sanctions ranging from, but not limited to, issuing a correction, reporting the inaccurate information to the authors' institution, banning authors from submitting work to ASN journals for varying lengths of time, and/or retraction of the published work.

Name: Kaitlin J. Mayne
Manuscript ID: CJASN-2024-000275R2
Manuscript Title: Frailty, multimorbidity and polypharmacy: exploratory analyses of the effects of empagliflozin from the EMPA-KIDNEY trial
Date of Completion: June 18, 2024
Disclosure Updated Date: April 18, 2024

ASN
American Society of Nephrology
JASN
Journal of the American Society of Nephrology
KIDNEY360
Accessing Our World From Every Angle
CJASN
Clinical Journal of the American Society of Nephrology

ASN Journal Disclosure Form

As per ASN journal policy, I have disclosed any financial relationships or commitments I have held in the past 36 months as included below. I have listed my Current Employer below to indicate there is a relationship requiring disclosure. If no relationship exists, my Current Employer is not listed.

M. Nangaku reports the following:
Employer: the University of Tokyo Graduate School of Medicine; Consultancy: Kyowa-Kirin, Tanabe-Mitsubishi, Boehringer-Ingelheim; Research Funding: Kyowa-Kirin, Chugai, Boehringer-Ingelheim; and Honoraria: Kyowa-Kirin, Tanabe-Mitsubishi.

I understand that the information above will be published within the journal article, if accepted, and that failure to comply and/or to accurately and completely report the potential financial conflicts of interest could lead to the following: 1) Prior to publication, article rejection, or 2) Post-publication, sanctions ranging from, but not limited to, issuing a correction, reporting the inaccurate information to the authors' institution, banning authors from submitting work to ASN journals for varying lengths of time, and/or retraction of the published work.

Name: Masaomi Nangaku
Manuscript ID: CJASN-2024-000275R2
Manuscript Title: Frailty, multimorbidity and polypharmacy: exploratory analyses of the effects of empagliflozin from the EMPA-KIDNEY trial
Date of Completion: June 18, 2024
Disclosure Updated Date: April 8, 2024

ASN Journal Disclosure Form

As per ASN journal policy, I have disclosed any financial relationships or commitments I have held in the past 36 months as included below. I have listed my Current Employer below to indicate there is a relationship requiring disclosure. If no relationship exists, my Current Employer is not listed.

D. Preiss reports the following:
Employer: University of Oxford; and Research Funding: Boehringer Ingelheim, Novartis, Novo Nordisk.

I understand that the information above will be published within the journal article, if accepted, and that failure to comply and/or to accurately and completely report the potential financial conflicts of interest could lead to the following: 1) Prior to publication, article rejection, or 2) Post-publication, sanctions ranging from, but not limited to, issuing a correction, reporting the inaccurate information to the authors' institution, banning authors from submitting work to ASN journals for varying lengths of time, and/or retraction of the published work.

Name: David Preiss
Manuscript ID: CJASN-2024-000275R1
Manuscript Title: Frailty, multimorbidity and polypharmacy: exploratory analyses of the effects of empagliflozin in CKD from the EMPA-KIDNEY trial
Date of Completion: April 23, 2024
Disclosure Updated Date: April 23, 2024

ASN
American Society of Nephrology
JASN
Journal of the American Society of Nephrology
KIDNEY360
Accessing Our World From Every Angle
CJASN
Clinical Journal of the American Society of Nephrology

ASN Journal Disclosure Form

As per ASN journal policy, I have disclosed any financial relationships or commitments I have held in the past 36 months as included below. I have listed my Current Employer below to indicate there is a relationship requiring disclosure. If no relationship exists, my Current Employer is not listed.

X. Rossello reports the following:
Other Interests or Relationships: European Society of Cardiology (ESC) Clinical Practice Guideline Committee member

I understand that the information above will be published within the journal article, if accepted, and that failure to comply and/or to accurately and completely report the potential financial conflicts of interest could lead to the following: 1) Prior to publication, article rejection, or 2) Post-publication, sanctions ranging from, but not limited to, issuing a correction, reporting the inaccurate information to the authors' institution, banning authors from submitting work to ASN journals for varying lengths of time, and/or retraction of the published work.

Name: Xavier Rossello
Manuscript ID: CJASN-2024-000275R2
Manuscript Title: Frailty, multimorbidity and polypharmacy: exploratory analyses of the effects of empagliflozin from the EMPA-KIDNEY trial
Date of Completion: May 28, 2024
Disclosure Updated Date: May 28, 2024

ASN American Society of Nephrology | JASN Journal of the American Society of Nephrology | KIDNEY360 Accessing Our World From Every Angle | CJASN Clinical Journal of the American Society of Nephrology

ASN Journal Disclosure Form

As per ASN journal policy, I have disclosed any financial relationships or commitments I have held in the past 36 months as included below. I have listed my Current Employer below to indicate there is a relationship requiring disclosure. If no relationship exists, my Current Employer is not listed.

E. Sammons reports the following:
Research Funding: Institutional awards from the following companies and charities have supported the EMPA-KIDNEY trial: Boehringer Ingelheim, Eli Lilly, UK Medical Research Council, British Heart Foundation, National Institute for Health and Care Research Biomedical Research Council, Health Data Research UK.

I understand that the information above will be published within the journal article, if accepted, and that failure to comply and/or to accurately and completely report the potential financial conflicts of interest could lead to the following: 1) Prior to publication, article rejection, or 2) Post-publication, sanctions ranging from, but not limited to, issuing a correction, reporting the inaccurate information to the authors' institution, banning authors from submitting work to ASN journals for varying lengths of time, and/or retraction of the published work.

Name: Emily Sammons
Manuscript ID: CJASN-2024-000275R1
Manuscript Title: Frailty, multimorbidity and polypharmacy: exploratory analyses of the effects of empagliflozin in CKD from the EMPA-KIDNEY trial
Date of Completion: April 18, 2024
Disclosure Updated Date: April 18, 2024

ASN American Society of Nephrology JASN Journal of the American Society of Nephrology KIDNEY360 Accessing Our World From Every Angle CJASN Clinical Journal of the American Society of Nephrology

ASN Journal Disclosure Form

As per ASN journal policy, I have disclosed any financial relationships or commitments I have held in the past 36 months as included below. I have listed my Current Employer below to indicate there is a relationship requiring disclosure. If no relationship exists, my Current Employer is not listed.

R. Sardell has nothing to disclose.

I understand that the information above will be published within the journal article, if accepted, and that failure to comply and/or to accurately and completely report the potential financial conflicts of interest could lead to the following: 1) Prior to publication, article rejection, or 2) Post-publication, sanctions ranging from, but not limited to, issuing a correction, reporting the inaccurate information to the authors' institution, banning authors from submitting work to ASN journals for varying lengths of time, and/or retraction of the published work.

Name: Rebecca J. Sardell
Manuscript ID: CJASN-2024-000275R2
Manuscript Title: Frailty, multimorbidity and polypharmacy: exploratory analyses of the effects of empagliflozin from the EMPA-KIDNEY trial
Date of Completion: May 29, 2024
Disclosure Updated Date: May 22, 2024

ASN American Society of Nephrology · JASN Journal of the American Society of Nephrology · KIDNEY360 Accessing Our World From Every Angle · CJASN Clinical Journal of the American Society of Nephrology

ASN Journal Disclosure Form

As per ASN journal policy, I have disclosed any financial relationships or commitments I have held in the past 36 months as included below. I have listed my Current Employer below to indicate there is a relationship requiring disclosure. If no relationship exists, my Current Employer is not listed.

N. Staplin reports the following:
Employer: Clinical Trial Service Unit, University of Oxford; Research Funding: Boehringer Ingelheim; Novo Nordisk; and Advisory or Leadership Role: Associate Editor for Nephrology Dialysis Transplant.

I understand that the information above will be published within the journal article, if accepted, and that failure to comply and/or to accurately and completely report the potential financial conflicts of interest could lead to the following: 1) Prior to publication, article rejection, or 2) Post-publication, sanctions ranging from, but not limited to, issuing a correction, reporting the inaccurate information to the authors' institution, banning authors from submitting work to ASN journals for varying lengths of time, and/or retraction of the published work.

Name: Natalie Staplin
Manuscript ID: CJASN-2024-000275R1
Manuscript Title: Frailty, multimorbidity and polypharmacy: exploratory analyses of the effects of empagliflozin in CKD from the EMPA-KIDNEY trial
Date of Completion: April 23, 2024
Disclosure Updated Date: May 16, 2023

ASN American Society of Nephrology JASN Journal of the American Society of Nephrology KIDNEY360 Accessing Our World From Every Angle CJASN Clinical Journal of the American Society of Nephrology

ASN Journal Disclosure Form

As per ASN journal policy, I have disclosed any financial relationships or commitments I have held in the past 36 months as included below. I have listed my Current Employer below to indicate there is a relationship requiring disclosure. If no relationship exists, my Current Employer is not listed.

K. Tuttle reports the following:
Employer: Providence Medical Research Center/Providence Inland Northwest Health; Consultancy: Boehringer Ingelheim, Novo Nordisk, Bayer, ELi Lilly; Research Funding: Bayer, Travere; Honoraria: Bayer, Novo Nordisk; and Advisory or Leadership Role: Chair, Diabetic Kidney Disease Collborative, American Society of Nephrology (unpaid).

I understand that the information above will be published within the journal article, if accepted, and that failure to comply and/or to accurately and completely report the potential financial conflicts of interest could lead to the following: 1) Prior to publication, article rejection, or 2) Post-publication, sanctions ranging from, but not limited to, issuing a correction, reporting the inaccurate information to the authors' institution, banning authors from submitting work to ASN journals for varying lengths of time, and/or retraction of the published work.

Name: Katherine R. Tuttle
Manuscript ID: CJASN-2024-000275R2
Manuscript Title: Frailty, multimorbidity and polypharmacy: exploratory analyses of the effects of empagliflozin from the EMPA-KIDNEY trial
Date of Completion: May 28, 2024
Disclosure Updated Date: March 20, 2024

ASN American Society of Nephrology
JASN Journal of the American Society of Nephrology
KIDNEY360 Accessing Our World From Every Angle
CJASN Clinical Journal of the American Society of Nephrology

ASN Journal Disclosure Form

As per ASN journal policy, I have disclosed any financial relationships or commitments I have held in the past 36 months as included below. I have listed my Current Employer below to indicate there is a relationship requiring disclosure. If no relationship exists, my Current Employer is not listed.

C. Wanner reports the following:
Employer: University Hospital; Consultancy: Alexion, AstraZeneca, Bayer, Boehringer-Ingelheim, GSK, Idorsia, MSD, NovoNordisk, CSL-Vifor; Research Funding: University of Oxford; Honoraria: Amgen, Amicus, Astellas, AstraZeneca, Bayer, Boehringer-Ingelheim, Chiesi, FMC, Eli-Lilly, GSK, Novartis, Sanofi, Stadapharm, Takeda, CSL-Vifor; and Other Interests or Relationships: European Renal Association.

I understand that the information above will be published within the journal article, if accepted, and that failure to comply and/or to accurately and completely report the potential financial conflicts of interest could lead to the following: 1) Prior to publication, article rejection, or 2) Post-publication, sanctions ranging from, but not limited to, issuing a correction, reporting the inaccurate information to the authors' institution, banning authors from submitting work to ASN journals for varying lengths of time, and/or retraction of the published work.

Name: Christoph Wanner
Manuscript ID: 2024-000275R2
Manuscript Title: Frailty
Date of Completion: June 2, 2024
Disclosure Updated Date: June 2, 2024

ASN American Society of Nephrology | JASN Journal of the American Society of Nephrology | KIDNEY360 Accessing Our World From Every Angle | CJASN Clinical Journal of the American Society of Nephrology

ASN Journal Disclosure Form

As per ASN journal policy, I have disclosed any financial relationships or commitments I have held in the past 36 months as included below. I have listed my Current Employer below to indicate there is a relationship requiring disclosure. If no relationship exists, my Current Employer is not listed.

D. Zhu reports the following:
Employer: Oxford Population Health, University of Oxford

I understand that the information above will be published within the journal article, if accepted, and that failure to comply and/or to accurately and completely report the potential financial conflicts of interest could lead to the following: 1) Prior to publication, article rejection, or 2) Post-publication, sanctions ranging from, but not limited to, issuing a correction, reporting the inaccurate information to the authors' institution, banning authors from submitting work to ASN journals for varying lengths of time, and/or retraction of the published work.

Name: Doreen Zhu
Manuscript ID: CJASN-2024-000275R2
Manuscript Title: Frailty, multimorbidity and polypharmacy: exploratory analyses of the effects of empagliflozin from the EMPA-KIDNEY trial
Date of Completion: June 18, 2024
Disclosure Updated Date: April 12, 2024
