# Supplementary material for: Frailty, Multimorbidity, and Polypharmacy: Exploratory Analyses of the Effects of Empagliflozin from the EMPA-KIDNEY Trial
Source: Clin J Am Soc Nephrol. 2024 Jun 27;19(9):1119–29. doi: 10.2215/CJN.0000000000000498 (PMC11390031; doi:10.2215/CJN.0000000000000498)
Supplement: Supplementary file 2 [file cjasn-19-1119-s002.pdf]

Frailty, multimorbidity and polypharmacy: exploratory analyses of the effects of empagliflozin from the EMPA-KIDNEY trial

The EMPA-KIDNEY Collaborative Group

SUPPLEMENTARY MATERIALS

Contents

Members of the EMPA-KIDNEY Collaborative Group 2
Membership of the Executive Committee, Steering Committee and Independent Data Monitoring Committee 2
Central and Regional Coordinating Centres 2
Central Coordinating Office 2
List of Collaborators, by Site 4
Supplementary Methods 13
Definitions 13
Safety Outcomes 14
Supplementary Statistical Methods 14
Supplementary Tables & Figures 16
Supplementary Table 1: Variables assessed as potential predictors of hospitalization 17
Supplementary Table 2: Univariable associations with hospitalization (logistic regression) 18
Supplementary Table 3: Incremental impact of each variable in the final multivariable logistic regression model to predict hospitalization 19
Supplementary Table 4: Other characteristics of participants at recruitment by predicted risk of hospitalization 20
Supplementary Table 5: Composition of multimorbidity subgroups 22
Supplementary Table 6: Number of participants who had discontinued randomised treatment at the end of follow-up and reasons for discontinuation according to (i) predicted risk of hospitalization; and (ii) number of concomitant medications. 23
Supplementary Table 7: Primary and secondary outcomes by predicted risk of hospitalization 24
Supplementary Table 8: Primary and secondary outcomes by multimorbidity 25
Supplementary Table 9: Primary and secondary outcomes by concomitant medication count 26
Supplementary Table 10: Primary and secondary outcomes by health-related quality of life (EQ-5D index value) 27
Supplementary Table 11: Safety outcomes by predicted risk of hospitalization 28
Supplementary Table 12: Safety outcomes by multimorbidity 29
Supplementary Table 13: Safety outcomes by concomitant medication count 30
Supplementary Table 14: Safety outcomes by health-related quality of life (EQ-5D index value) 31
Supplementary Figure 1: Performance of the final multivariable logistic regression model in predicting hospitalization and all-cause death 32
Supplementary Figure 2: Associations between predicted risk of hospitalization and multimorbidity; polypharmacy; and health-related quality of life 33
Supplementary Figure 3: Number of participants in the highest level of frailty (defined as predicted risk of hospitalization >45%) in EMPA-KIDNEY showing overlap with conventional definitions of multimorbidity and polypharmacy (which differ from Figure 1) 34
Supplementary Figure 4: Effects of empagliflozin versus placebo on recurrent all-cause hospitalization by frailty (based on predicted risk of hospitalization), multimorbidity, polypharmacy and health-related quality of life 35
Supplementary Figure 5: Effects of empagliflozin versus placebo on recurrent all-cause hospitalization by key pre-specified subgroups 36
Supplementary Figure 6: Effects of empagliflozin versus placebo on weight and blood pressure by predicted risk of hospitalization 37

Members of the EMPA-KIDNEY Collaborative Group

Membership of the Executive Committee, Steering Committee and Independent Data Monitoring Committee

Executive Committee
Colin Baigent (co-Chair), Martin J. Landray (co-Chair), Christoph Wanner (Deputy Chair), William G. Herrington (Chief Investigator), Richard Haynes (co-Principal Investigator), Jennifer B. Green, Sibylle J. Hauske*, Martina Brueckmann*, Mark Hopley*
(Previous members: Maximillian von-Eynatten* & Jyothis George*)

Steering Committee
Executive Committee members plus National Representatives: Susanne Brenner (Germany); Alfred K. Cheung (United States); David Preiss (United Kingdom); Zhi-Hong Liu, Jing Li (China); Laiseong Hooi, Wen Liu (Malaysia); Takashi Kadowaki, Masaomi Nangaku (Japan); Adeera Levin, David Cherney (Canada); Roberto Pontremoli, Aldo P. Maggioni (Italy); plus statistician members: Natalie Staplin, Jonathan Emberson, Stefan Hantel*; plus other expert members: Shinya Goto, Rajat Deo, Katherine R. Tuttle. Non-voting members: Michael Hill, Parminder Judge, Kaitlin J. Mayne, Sarah Y.A. Ng, Xavier Rossello, Emily Sammons, Doreen Zhu
* denotes a Boehringer Ingelheim employee

Independent Data Monitoring Committee
Peter Sandercock (Chair), Rudolf Bilous, Charles Herzog, Paul Whelton, Janet Wittes, Derrick Bennett (non-voting statistician)

Central and Regional Coordinating Centres

Central Coordinating Office

Administration: Patricia Achiri, Chrissie Ambrose, Cristina Badin, Jill Barton, Richard Brown, Andy Burke, Sebastian Butler, Rejive Dayanandan, Pia Donaldson, Robert Dykas, Lucy Fletcher, Kate Frederick, Hannah Kingston, Mo Gray, Emily Harding, Akiko Hashimoto, Lyn Howie, Susan Hurley, Ryonfa Lee, Nik Luker, Kevin Murphy, Mariko Nakahara, John Nolan, Michelle Nunn, Sorcha Mulligan, Akiko Omata, Sandra Pickworth, YanRu Qiao, Shraddha Shah, Karen Taylor, Alison Timadjer, Monique Willett, Liz Wincott, Qin Yan, Hui Yu; *Clinical*: Louise Bowman, Fang Chen, Robert Clarke, Michelle Goonasekera, Richard Haynes, William G. Herrington, Parminder Judge, Waseem Karsan, Marion Mafham, Kaitlin J. Mayne, Sarah Y. A. Ng, David Preiss, Christina Reith, Emily Sammons, Mohammed Zayed, Doreen Zhu; *Data Analysis*: Ritva Ellison, Rowan Moys, Will Stevens, Kevin Verdel, Karl Wallendszus; *Finance*: Chris Bowler, Anna Brewer, Andy Measor; *IT Validation*: Guanguo Cui, Charles Daniels, Angela Field, Bob Goodenough, Ashley Lawson, Youcef Mostefai, Dheeptha Radhakrishnan, Samee Syed, Shuang Xia; *Laboratory*: Ruth Adewuyi-Dalton, Thomas Arnold, Anne-Marie Beneat, Anoushka Bhatt, Chloe Bird, Andrew Breach, Laura Brown, Mark Caple, Tatyana Chavagnon, Karen Chung, Sarah Clark, Luminita Condurache, Katarzyna Eichstadt, Marta Espino Obrero, Scarlett Forest, Helen French, Nick Goodwin, Andrew Gordon, Joanne Gordon, Cat Guest, Tina Harding, Michael Hill, Michal Hozak, Matthew Lacey, David MacLean, Louise

Messinger, Stewart Moffat, Martin Radley, Claire Shenton, Sarah Tipper, Jon Tyler, Lesley Weaving, James Wheeler, Elissa Williams, Tim Williams, Hamish Woodhouse; *Monitoring*: Angela Chamberlain, Jo Chambers, Joanne Davies, Denise Donaldson, Pati Faria-Shayler, Denise Fleming-Brown, Jennifer Ingell, Carol Knott, Anna Liew, Helen Lochhead, Juliette Meek, Isabel Rodriguez-Bachiller, Andrea Wilson, Patrick Zettergren; *Programming*: Rach AitSadi, Ian Barton, Alex Baxter, Yonghong Bu, Lukasz Danel, Sonja Grotjahn, Rijo Kurien, Michael Lay, Archie Maskill, Aleksandra Murawska, Rachel Raff, Allen Young; *Principal Investigators*: Colin Baigent, Richard Haynes, William G. Herrington, Martin J. Landray, David Preiss; *Statistics*: Jonathan Emberson, Rebecca Sardell, Natalie Staplin

Regional Coordinating Centres

Germany (Universitätsklinikum Würzburg): Christoph Wanner, Susanne Brenner, Vladimir Cejka, Marcela Fajardo-Moser, Christian Hartner, Doris Poehler, Janina Renner, Franziska Scheidemantel

United States (Duke Clinical Research Institute [DCRI]): Jennifer B. Green, Miya Bryant, Anita Hepditch, Cassandra Johnson, Erin Latore, Yolanda Miller, Lauren Price, Merilee Whalen, Ashleigh Wheeler

UK (Clinical Trial Service Unit and Epidemiological Studies Unit [CTSU], University of Oxford): Richard Haynes, David Preiss, Cristina Badin, Jo Chambers, Joanne Davies, Denise Donaldson, Mo Gray, Emily Harding, Jenny Ingell, Yanru Qiao, Shraddha Shah, Andrea Wilson, Patrick Zettergren

China (National Center for Cardiovascular Disease, Fuwai Hospital & National Clinical Research Center of Kidney Diseases, Jinling Hospital, Nanjing University School of Medicine): Zhi-Hong Liu, Jing Li, Yu An, Yinghua Chen, Peiling Chen, Hao Dai, Hong Du, Fang Feng, Qing Guo, Libo Hou, Wuhanbilige Hundei, Binbin Jin, Yan Li, Jiamin Liu, Xia Song, Yanping Wang, Yanwu Yu, Ning Zhang, Lingshan Zhao, Hui Zhong

Malaysia (Klinsel SDN BHD): Cheng Beng Goh, Ye Mun Low, Soon Yi Sor, Farah Hanis Zulkipli, Sarojini Sivanandam

Japan (Parexel): Natsuki Arai, Ai Fukasawa, Mizue Furukawa, Keisuke Habuki, Shoko Hayashi, Wakako Isari, Saki Kanegae, Maria Kawai, Reiki Kobayashi, Takako Kuramae, Chika Kuribayashi, Sawako Maeno, Satoshi Masumoto, Tomoko Morisaki, Minoru Oda, Kazue Sawada, Kenta Sugamori, Ayana Tatsuzawa, Aiko Tomita, Kazuyuki Yuasa, Hiroko Inazawa

Canada (Providence Health Care, Vancouver): Adeera Levin, Amanda Axler, Kerri Gallo

Italy (ANMCO Research Center): Aldo P. Maggioni, Ester Baldini, Barbara Bartolomei Mecatti, Francesca Bianchini, Martina Ceseri, Laura Cipressa, Gianna Fabbri, Andrea Lorimer, Donata Lucci

List of Collaborators, by Site

Germany: *Universitatsklinikum Wurzburg*: Christoph Wanner, Susanne Brenner, Vladimir Cejka, Sharang Ghavampour, Anja Knoppe; *Zentrum fuer Nieren-, Hochdruck- und Stoffwechselerkrankungen Hannover*: Hans Schmidt-Gurtler, Hubert Dumann, Sybille Merscher, Margret Patecki, Georg Rainer Schlieper, Anke Torp, Bianca Weber, Maja Zietz; *Nephrologisches Zentrum Villingen-Schwenningen*: Bernd Hohenstein, Urs Benck, Diliana Draganova, Thomas Weinreich, Lothar Wolf, Jasmine Gaidu, Hanna Reiner, Mandy Visnjic; *Nierenzentrum Freiburg*: Daniel Steffl, Marie Breitenfeldt, Annette Kraemer-Guth, Christine Braun, Simone Hagge*; Dialysezentrum Heilbronn*: Michael Schomig, Stephan Matthias, Dominik Stoffler, Beate Schumacher; *Klinikum der Universitat Munchen*: Thomas Sitter, Louise Fuessl, Julia Krappe, Jerome Loutan, Volker Vielhauer, Luciano Andriaccio, Magdalena Maurer; *ClinPhenomics GmbH Co. KG*: Bernhard Winkelmann, Martin Dursch, Linda Seifert, Linda Tenbusch; *Universitatsmedizin Mainz*: Julia Weinmann-Menke, Simone Boedecker, Wiebke Kaluza-Schilling, Daniel Kraus, Carina Krieger, Margit Schmude, Anne Schreiber, Ewelina Eckrich; *Herz- und Diabeteszentrum Nordrhein-Westfalen*: Diethelm Tschope, Abdulwahab Arbi, Young Lee-Barkey, Bernd Stratmann, Natalie Prib, Sina Rolfsmeier, Irina Schneider; *Universitatsklinikum Dusseldorf*: Lars Rump, Johannes Stegbauer, Christine Pötz, Mara Schemmelmann, Claudia Schmidt; *Nephrocare Mettmann - Standort Velbert*: Michael Koch, Sendogan Aker, Annika Küpper, Manuela Martin; *Diaverum MVZ Potsdam*: Thiemo Pfab, Christian Albert, Michael Haase, Barbara Zander, Claudia Schneider-Danwitz; *Praxis fur Dialyse und Nierenkrankheiten - Arztezentrum Helle Mitte*: Wolfgang Seeger, Wolf-Adam Seeger, Britta Zemann; *Klinikum Bielefeld*: Christoph Stellbrink, Kristin Marx, Ekaterina Stellbrink, Britta Brettschneider, Stephanie Watson, Marion Iselt; *Studienzentrum Aschaffenburg*: Gerhard Klausmann, Inga-Nadine Kummer, Auguste Kutschat, Simone Streitenberger; *Universitatsklinikum Halle*: Matthias Girndt, Silke Markau, Ina Girakossyan, Claudia Hanf; *Klinikum St. Georg Leipzig*: Joachim Beige, Ralph Wendt, Ulrike Schmidt; *Studienzentrum Nephrologie Nurnberg-Langwasser*: Andreas Schneider, Roland Veelken, Claudia Donhauser, Auguste Kutschat; *UBAG fur Nephrologie und Dialyse Neckarsulm*: Luis Becker, Nexhat Miftari, Ricarda Wolfling, Sarah Morlok; *Universitatsklinikum Dresden*: Christian Hugo, Alexander Paliege, Jens Passauer, Julian Stumpf, Annegret Fleischer, Kerstin Haaser; *Universitatsklinikum Mannheim*: Bernhard Kraemer, Jan Jochims, Bernd Kruger, Claudia Foellinger, Anastassiya Reisler; *Nierenzentrum Wiesbaden*: Frank Strutz, Stefan Haack, Ursula Hohenstatt; *Universitatsklinikum Jena*: Martin Busch, Konstantin Herfurth, Gunter Wolf, Rainer Paul; *Studienzentrum fur Nieren- und Hochdruckerkrankungen Hannover*: Hermann Haller, Jessica Kaufeld, Jan Menne, Elisabeth Bahlmann-Kroll, Angela Bergner; *Universitatsklinikum Augsburg*: Horst Weihprecht, Aydin Er, Florian Sonntag, Elif Turan, Michael Wittmann, Franziska Klauser, Eva Voigt; *Nephrologisches Zentrum Gottingen*: Volker Schettler, Egbert Schulz, Madlen Rohnstock, Elke Schettler; *Universitaetsklinik Ulm*: Bernd Schroppel, Rene van Erp, Martin Kachele, Ulla Ludwig, Lena Schulte-Kemna, Waltraud Kmietschak, Elke Preiss, Martina Ruocco; *AGAPLESION Markus-Krankenhaus*: Gunnar Heine, Martin Brzoska, Sebastian Gabel, Christina Büttner, Asma Sabarai; *Universitatsklinikum Regensburg*: Bernhard Banas, Tobias Bergler, Yvonne Ehrl, Franz Putz, Antonia Schuster, Stefanie Kuhn, Torsten Schramm; *DaVita Viersen - Nettetal*: Stefan Degenhardt, Gerhard Schmidt, Lea Weiland, Ulrike Giebeln-Hudnell; *Klinikum*

Braunschweig: Jan Kielstein, Gabriele Eden, Brigitte Fuchs, Gina Morig, Manuela Winkler; *Nephrocare Mettmann*: Michael Koch, Sendogan Aker, Annika Küpper, Manuela Martin; *Vivantes Klinikum Neukolln*: Harald Darius, Charalampos Kriatselis, Carl-Philipp Roesch; Astrid Maselli, *Robert-Bosch-Krankenhaus Stuttgart*: Dominik Alscher, Markus Ketteler, Moritz Schanz, Severin Schricker, Bianka Rettenmaier, Andrea Schwab

United States: *Clinical Advancement Center*: Pablo Pergola, Irene Leal, Melissa Cagle, Anna Romo, Anthony Torres; *Seacoast Kidney and Hypertension Specialists*: Sucharit Joshi, Kulli Barrett, Alexis Africano, Vicki Dodds, Dorleena Gowen, Ashlee Morris; *Total Research Group, LLC*: Juan Fernandez, Guillermo Jimenez, Ricardo Viera, Kendaling Bruce, Ryan Barrios, Maylin Garcia, Kerelyn Garcia, Iradis Leal; *Nephrology Consultants, LLC*: David Tietjen, David Bains, Carlo Castillo, Genielle Brewer, Justin Davis, Natalie Freking, Brittany Golson, Sally Ham, Jesslyn Roesch; *Sumter Medical Specialists*: Pusadee Suchinda, Shameem Beigh, Usah Lilavivat, Joyce Bilton, Kim Bocchicchia; *Yale University*: Jeffrey Turner, Neera Dahl, Aldo Peixoto, Yasemin Kavak, Lauren Liberti, Hari Nair, Nicolas Page, Stephanie Rosenberg, Kathryn Simmons; *Northwestern University*: Tamara Isakova, Rebecca Frazier, Rupal Mehta, Anand Srivastava, Patrick Fox, Jonathan Hecktman, Alexander Hodakowski, Carlos Martinez, Rachel Phillips, Alexis Stevenson; *University of Kansas Medical Center*: Reem Mustafa, Kyle Jansson, Cassandra Kimber, Jason Stubbs, Ahmad Tuffaha, Sri Yarlagadda, Debbie Griffin, Elisabeth Laundy, Zhuo Tang; *Providence Sacred Heart Medical Center and Childrens Hospital*: Radica Alicic, Katherine R. Tuttle, Ann Cooper, Lisa Davis; *East Coast Research Institute*: Ashwini Gore, Rebecca Goldfaden, Leslie Harvill, Lisa Hichkad, Barry Johns, Thomas Jones, Kayla Merritt, Jennifer Sheldon, Jennifer Stanfield, Lindsay Alexander, Kaitlyn Preston, Lindsey Wood; *Monument Health*: Rajesh Pradhan, Roger DeRaad, Kelli McIntosh, Louis Raymond, Michael Shepperd, Susan McLaughlin, Mary Seifert, Andrew Shepherd; *Mountain Kidney & Hypertension Associates*: Joseph Aiello, William Durham, Laurie Loudermilk, John Manley, Sabrina Burnette, Stephanie Evans, Tara Johnson; *Texas Institute for Kidney and Endocrine Disorders*: Lance Sloan, Judy Ann Acosta, Stacy Gillham, Katia Sloan, SueAnn Squyres; *Wake Forest University Health Sciences*: Michael Rocco, Amret Hawfield, Ben Bagwell, Lauren Richmond; *Chase Medical Research*: Joseph Soufer, Subha Clarke, Amanda Aliu, Kristine Calabrese, Amanda Davis, Veronica Poma, Tracy Spinola; *East Coast Institute for Research LLC*: James Magee, Ricardo Silva, Rushab Choksi, Lorraine Dajani, John Evans, Anil George, Rebecca Goldfaden, Prasanth Krish, Gerard Martins, Mae Sheikh-Ali, David Sutton, Freda Driver, Abraham Hanburry, Laura Hume, Amber Hurst, Matthew Taddeo, Marla Turner, Veronica Yousif; *University of Utah Health Sciences*: Srinivasan Beddhu, Laith Al-Rabadi, Nikita Abraham, Amalia Caamano, Judy Carle, Victoria Gonce, Kaitlyn Staylor, Na Zhou; *University of Texas Health Science Center at San Antonio*: Shweta Bansal, Manoj Bhattarai, Kumar Sharma, Subrata Debnath, Aliseiya Garza, Chakradhar Velagapudi; *Academy of Diabetes, Thyroid, and Endocrine, PA*: Sergio Rovner, Javier Almeida, Pablo Casares, Verlaine Stewart-Ray, Rene Almaraz, Renata Dayrell, Ana Moncada, Ricardo Pulido, Roxana Rodriquez; *East Coast Institute for Research*: James Magee, Wasim Deeb, Kathryn DeGoursey, Rodel Gloria, Trevor Greene, Robert Miller, Edward Pereira, Miguel Roura, Mae Sheikh-Ali, David Sutton, Debbie Domingo, Sasha Dorestin, William Hodge, Cathy Jackson, Deborah Lund, Katrina Taylor; *Aventiv Research*: Kenneth Boren, Brittany Cleveland, Sandra Gaiser, Mandeep

Sahani, Logan Aldrich, Exodus Edmerson, Edmond Limon, Cole Valletta, Patricia Vasquez; *St. Clair Nephrology Research*: Christopher Provenzano, Navkiranjot Brar, Heather Henderson, Bellovich Keith, Qur Khai, Quresh Khairullah, Gail Makos, Joel Topf, Sherry Gasko, Rosemarie Henschel, Kaitlin Knapp, Teresa Kozlowski, Paula LaFleur, Ashwathy Varughese; *Kaiser Permanente San Diego*: Hui Xue, Patricia Wu, Olga Arechiga, Shan Darbeau, Michael Fechter, Stephanie Martinez; *Hanson Clinical Research Center*: Lenita Hanson, Nyla Cooper, Arelis Madera, Jay Cadorna, Rita Sheridan, Helen Sparks; *Saint Elizabeth Healthcare*: Bradley Eilerman, Susanne Bodine, Wael Eid, Rebecca Flora, Amber Avery, Cashmere Hardy; *Thomas Jefferson/ARIA Health Northeast Endocrine Metabolic Associates*: Mihaela Biscoveanu, Steven Nagelberg, Tracey Cummins; *Emory University*: Frederic Rahbari-Oskoui, Anju Oommen, Zohreh Forghani, Stacie Hitchcock, Darya Hosein, Diane Watkins; *East Coast Institute Research, LLC*: Minesh Patel, Anthony Lambert, Elizabeth Newman, Autumn Wood, Tammy Ross, Stephany Topping; *Kidney Care and Transplant Services of New England*: Jeffrey Mulhern, Lorna Murphy, Ann Vasseur; *Brookview Hills Research Associates LLC*: Gregory Greenwood, Alexander Hadley, Denise Laurienti, Christopher Marshall, Nicholas McLean, Scott Satko, Brandy Caudill, Jacob Maris, Janice Rogers, Cindy Vanhoy; *Cleveland Clinic*: George Thomas, Georges Nakhoul, John O'Toole, Jonathan Taliercio, Leslie Cooperman, Marina Markovic, Barbara Tucky; *Salem V.A. Medical Center*: Devasmita Dev, Alia Hasan, Hima Yalamanchili, Namita Jain, Lesley McNeil, Eric Wines; *Medstar Health Research Institute*: Jean Park, Adline Ghazi, Mia Hamm, Tejas Patel; *University of North Carolina Hospital*: Amy Mottl, Emily Chang, Vimal Derebail, Emmie Cole, Anne Froment, Sara Kelley, Jordan Osmond Foster; *Olive View - UCLA Medical Center*: Vahid Mahabadi, Golriz Jafari, Anita Kamarzarian, Wendy Arriaga, Daisy Arteaga, Rosario Machicado, Genesis Naverrete; *P&I Clinical Research, LLC*: Prashant Kumar, Imran Nazeer, Karina Urquia, Tammi Glider, Vickie Jones, Savannah Rucker, Jennifer Wiley; *Pioneer Research Solutions*: Rahul Pandey, Jesus Arroyo, Harish Pariani, Mohammad Ahmad, Shahin Mozaffari, Erika Perez; *Los Angeles Biomedical Research Institute at Harbor-UCLA Medical Center*: Matthew Budoff, Sion Roy, Divya Birudaraju, Ahmed Ghanem, Sajad Hamal; *Research Institute of Dallas*: Stephen Aronoff, Elisa Joye Petr, Richard Sachson, Jaime Wiebel, Sana Akram, Laurie Jones, Curtis Knight; Maurie Tarlac; *Renal Disease Research Institute*: Shahbaz Ahmed, Harold Szerlip, Akinwande Akinfolarin, Ankit Mehta, Shana Camp, Cindy Castro, Zanaida Cooper, Jessica Terry; *Clinical Research Consultants*: Ahmed Awad, Bhavya Kothapalli, Ryan Lustig, Serine Alfaress, Hyder Jasim, Mary Parrigon; *Lexington V.A. Health Care System*: Dennis Karounos, Sadiq Ahmed, Maggie Berry, Ruth Oremus; *VA Southern Nevada Healthcare System*: Carlos Hernandez-Cassis, Elias Ugwu, Nazia Junejo, Nancy Suazo; *University of Florida Health*: Mark Segal, Amir Kazory, Sherry Brown, Tristan Daniels, Sofia Dayi, Renee Hogan, Kathy McCray, Jennifer Stickley; *University Hospitals Cleveland Medical Center*: Mahboob Rahman, Mirela Dobre, Lavinia Negrea, Aparna Padiyar, Nishigandha Pradhan, Arash Rashidi, Nagaraju Sarabu, Vicki Donley, Tricia Young; *Midland Florida Clinical Research Center*: Godson Oguchi, Judepatricks Onyema, Kahla Damianik, Jack Dienes, Judith Plummer-Morgan, Marilyn Roman, Mauver Skipper, Stacey-Ann Villaruel, Krystle Williams; *Cedar Crosse Research Center*: Danny Sugimoto, Jeffrey Dugas, Ismeal Ahmed, Jamie Bhairoo, Dolores Rijos, Huzaifa Salim

UK: *Oxford University Hospitals*: Richard Haynes, William G. Herrington, Doreen Zhu, Madita Gavrila, Kathryn Lafferty, Ria Rabara, Sally Ruse, Maria Weetman; *Southmead*

Hospital, Bristol: James Bushnell, Albert Power, Alison Jenkins, Stefanie Jones, Amanda Scott; *Nottingham City Hospital*: Cath Byrne, Mark Jesky, Alison Cowley, Emma McHaffie, Holly Waterfall; *Dorset County Hospital*: Jo Taylor, Laura Bough, Thomas Phillips, Barbara Winter-Goodwin; *King's College Hospital, London*: Sui Phin Kon, Iain MacDougall, Eirini Lioudaki, Sapna Shah, Claire Sharpe, Francisco Aguilar, Abegail Hernandez Pena, Conception Pugay, Amelia Te; *Queen Elizabeth Hospital Birmingham*: Hugh Finn, Wasim Hanif, Samiul Mostafa, Alice Aitken, Katharine Draxlbauer, Evelina Grobovaite, Jennifer Kearney, Theresa McCarthy; *Royal Cornwall Hospital*: Giorgio Gentile, Duncan Browne, Palanichamy Chellamuthu, Tabinda Dugal, Terri Chant, Laura Jones, Emily Laity, Megan Miners, James Muir, Elizabeth Swanson; *Imperial College Healthcare NHS Trust*: Andrew Frankel, James Tomlinson, Marlon Alegata, Rashid Almasarwah, Anthoula Apostolidi, Maria Vourvou, Thomas Walters; *Royal Derby Hospital*: Maarten Taal, Hari Dukka, Nitin Kolhe, Carly McDonald, Kelly White; *The Queen Elizabeth Hospital, King's Lynn*: Shiva Ugni, Smita Gunda, Rotimi Oluyombo, Vicki Brindle, Ping Coutts, Tracy Fuller, Evelyn Nadar; *Princess Royal Hospital, Telford*: Suresh Ramadoss, Denise Donaldson, Nichola Motherwell, Susannah Pajak, Louise Tonks; *Hull Royal Infirmary*: Sunil Bhandari, Richard Bodington, Adil Hazara, Dominic Fellowes; *University Hospital Aintree*: Christopher Wong, Christopher Goldsmith, Sherald Barnes, Ann Bennett, Claire Burston, Samantha Hope, Nicola Hunt, Lini Kurian; *UHNM Royal Stoke University Hospital*: Richard Fish, Daniela Farrugia, Judy Lee, Emma Sadler, Hannah Turner; *Belfast City Hospital*: Christopher Hill, Henry Brown, Agnes Masengu, Peter Maxwell, Nina Bleakley, Hugh Murtagh; *West Suffolk NHS Foundation Trust*: William Petchey, Vivian Yiu, Joanne Kellett, Angharad Williams; *Royal Devon and Exeter Hospital*: Helen Clarke, Victoria Carnall, Sarah Benyon, Caroline Blake, Stephanie Estcourt, Jane Piper; *Daisy Hill Hospital*: Neal Morgan, Carolyn Hutchinson, Teresa McKinley; *Ulster Hospital, Dundonald*: Alastair Woodman, Judi Graham, Niall Leonard, John Smyth, Vicki Adell, Samantha Hagan; *Royal Free London NHS Foundation Trust*: Ben Caplin, Amin Oomatia, Eleanor Damian, Toluleyi Sobande; *Kent & Canterbury Hospital*: Tim Doulton, Michael Delaney, Mahmoud Montasser, Jenny Hansen, David Loader, Angela Moon, Frances Morris; *Salford Royal NHS Foundation Trust*: Smeeta Sinha, Chukwuma Chukwu, Amy Hudson, Diane Campbell, Melanie Kershaw, Stephanie Whittaker; *Brighton and Sussex University Hospital's NHS Trust*: Ayesha Irtiza-Ali, Farid Ghalli, Heba Nosseir, Allison Leslie, Kate Trivedi; *University Hospital of Wales, Cardiff*: Donald Fraser, Mohammad Alhadj Ali, Sian Griffin, Farah Latif, Justyna Witczak, Alexa Wonnacott, Lynda Jeffers, Yvette Webley; *Edinburgh Royal Infirmary*: Paul Phelan, Eve Miller-Hodges, Ailsa Geddes, Margaret Glenwright, Amy Hunter; *Gloucestershire Hospitals NHS Foundation Trust*: Thomas Pickett, Jim Moriarty, Linda Hill, Amanda Tyler; *University Hospitals Coventry and Warwickshire*: Waqar Ayub, Gail Evans, Sue Hewins, Davina Hewitt, Kerry Read; *Ninewells Hospital*: Samira Bell, Leanne Cosgrove, Rachel Craik, Shona Murray; *Royal Berkshire Hospital, Reading*: Nitin Bhandary, Holly Coles, Rashmi Easow, Maya Joseph; *Northern General Hospital, Sheffield*: Arif Khwaja, Yvonne Jackson, Angeline Mbuyisa, Rachel Sellars; *Darent Valley Hospital, Dartford*: Nihil Chitalia, Cynthia Mohandas, Anca Gherman, Charlotte Kamundi, Olumide Olufuwa; *Royal London Hospital*: Kieran McCafferty, Adedolapo Adeleke, Cara Healy, Damini Jeyarajah, Edward Kinsella-Perks; *Ipswich Hospital*: Richard Smith, Brian Camilleri, Carol Buckman, Jenny Finch, Vanessa Rivers; *University Hospitals Plymouth NHS Trust*: Andrew Connor, Sheila Carr, Lisa Shainberg; *Cheltenham General Hospital*: Thomas Pickett, Linda Hill, Amanda Tyler; *St. James's University Hospital, Leeds*: Andrew

Lewington, Richard Baker, Suzannah Dorey, Kay Tobin, Rosalyn Wheatley; *St. George's University Hospitals NHS Foundation Trust*: Debasish Banerjee, Richard Hull, Sharirose Abat, Riny Paul; *Norfolk and Norwich University Hospitals*: Mahzuz Karim, Zay Htet, Rotimi Oluyombo, Saad Tufail, Ravi Varma, Karen Convery, Deirdre Fottrell-Gould, Lisa Hudig, Emily Tropman; *Walsall Healthcare NHS Trust*: Thahir Abdul-Samad, Anne Grace, Marie Phipps; *St Helier Hospital, Carshalton*: Rebecca Suckling, Subash Somalanka, Bhrigu Sood, Pauline Swift, Sarah Acheampong, Kwame Ansu, Martia Augustin; *Wessex Kidney Centre, Queen Alexandra Hospital, Portsmouth*: Anna Sampson, Lynn Vinall, Kim Wren; *St Bartholomew's Medical Centre*: Shamila Wanninayake, Nicholas Wooding, Heather Edwards, Lydia Owen; *Antrim Area Hospital*: Stephanie Bolton, Marion Carson, Michael Matthews; *University Hospitals of Leicester*: Nigel Brunskill, Jorge Jesus-Silva, Alex Howson, Mary Quashie-Akponeware; *North Middlesex University Trust Diabetes Department, North Middlesex University Hospital*: Hilary Tindall, Chidambaram Nethaji, Helen Eldon; *Glasgow Clinical Research Facility, Queen Elizabeth University Hospital*: Rajan Patel, Patrick Mark, Alastair Rankin, Michael Sullivan, Kirsty Forsyth, Rowan McDougall; *Great Western Hospital, Swindon*: Tanaji Dasgupta, Louisa Davies, Maggie Ryder; *Hathaway Medical Centre, Chippenham*: Philip Grimmer, Clare Macdonald, Mary Webster; Newcastle: Timothy Ellam, Edwin Wong, Christine Meshykhi, Andrea Webster, Peter Wilson; *Lister Hospital*: Enric Vilar, Jocelyn Berdeprado, Eunice Doctolero, Lily Wilkinson; *Altnagelvin Hospital, Western Health & Social Care Trust*: Frank McCarroll, Hesham Ammar, Ying Kuan, Conor Moran, Girish Shivashankar, Ryan Campbell, Deborah Glowski, Paula McDermott; *Oakenhurst Medical Practice, Blackburn*: Amar Ali, Zuber Patel, Christine Bond, Gillian Whalley

China: *National Clinical Research Center of Kidney Diseases, Jinling Hospital, Nanjing University School of Medicine*: Haitao Zhang, Peiling Chen, Yu An, Yinghua Chen, Liu Yang, Lihua Zhang, Tingting Kan, Ling Zhu; *The Second Affiliated Hospital of Army Medical University, PLA*: Jinghong Zhao, Weiping Hou, Jing Wu; *Beijing Anzhen Hospital, Capital Medical University*: Hong Cheng, Weijing Bian, Zhirui Zhao; *Henan Provincial People's Hospital*: Fengmin Shao, Huixia Cao, Xiaojing Jiao, Peiyuan Niu; *Shanghai Fifth People's Hospital, Fudan University*: Jianying Niu, Yu Chen, Lihong Zhang; *Huazhong University of Science and Technology Union Shenzhen Hospital*: Shenglang Zhu, Haiyan Lin, Shaopeng Yao, Jiehui Chen, Ying Jiang; *The second affiliated hospital of Zhejiang University School of Medicine*: Ying Hu, Huaying Xiao, Fuye Yang; *Shenzhen People's Hospital*: Xinzhou Zhang, Baochun Guo, Qiu Jin, Lixia Liu; *Xiangya Hospital, Central South University*: Xiangcheng Xiao, Yanyun Xie, Ting Meng; *Wuhan Fourth Hospital*: Chuanwen Xu, Jie Huang, Yanmei Xu; *Suzhou Kowloon Hospital*: Weixin Kong, Xiaoliang Wang, Qianpan Liu,; *Jinzhou Central Hospital*: Xueying Wang, Ming Gao; *Zhuzhou Central Hospital (Nephrology)*: Xiumei Hu, Ying Lu; *Sichuan Provincial People's Hospital*: Li Wang, Kun Peng, Wei Wang; *Fuwai Hospital, Chinese Academy of Medical Sciences*: Qiuhong Gong, Jianfang Cai, Xiaojue Li, Xuejiao Liu, Haitao Zhang, Shuhan Zhou; *Zhuzhou Central Hospital (Endocrinology)*: Hong Liu, Yao Weng, Shuai Tang, Yao Yao; *The Central Hospital of Wuhan*: Shi Zhao, Chen Cheng, Wei Wei, Na Li

Malaysia: *Hospital Kajang*: Sadanah Aqashiah Mazlan, Alia Zubaidah Bahtar, Elliyyin Katiman, Noraini Othman; *Hospital Tuanku Ja'afar*: Lily Mushahar, Nurdiana Mazlan, Nur Sharafina Safiee, Sarasa Ramasamy; *Hospital Selayang*: Hin Seng Wong, Hajar Ahmad Rosdi, Esther Zhao Zhi Tan, Ju Fan Tay; *Hospital Taiping*: Kok Seng Teng,

Hasnah Yahaya; *Hospital Sultanah Aminah*: Wen Jiun Liu, Lik Wee Ee, Kenneth Kay Leong Khoo, Yuana Mohd Yusoff; *Hospital Tengku Ampuan Afzan*: Fariz Safhan Mohamad Nor, Mohd Kamil Ahmad, Mohd Ramli Seman; *Hospital Umum Sarawak*: Clare Hui Hong Tan, Laura Lui Sian Ngu, Jaime Yoke May Chan, Javelin Peji; *Hospital Raja Permaisuri Bainun*: Chek Loong Loh, Yee Yan Lee, Sridhar Ramanaidu, Kah Mean Thong, Yik Hong Wong, Suria Junus; *Hospital Sultanah Bahiyah*: Chen Hua Ching, Mohammad Faisal Asmee, Ku Ruziana Ku Md Razi, Chun Leong Low, Christopher Sze Bing Sim, Zhang Duan Tham, Noor Kamila Abdullah; *Hospital Sultan Abdul Halim*: Tai Meng Chen, Yong Chieh Chan, Eason Chang, Huan Yean Kang, Kai Quan Lee, Sue Ann Lee, Aik Kheng Lee, Jeevika Vinathan; *Universiti Kebangsaan Malaysia Medical Centre*: Rizna Abdul Cader, Ruslinda Mustafar, Lydia Kamaruzaman, Rozita Mohd, Rahimah Ismail; *Hospital Kulim*: Chong Men Leong, Chee Koon Low, Liang Wei Wong, Norlezah Adnan, Sabariah Ibrahim; *Hospital Kuala Lumpur*: Mohamad Zaimi Abdul Wahab, Sunita Bavanandan, Yik Shen Lim, Zhang Duan Tham, Wan Hazlina Wan Mohamad, Siti Munirah Jaafar, Nur Ashykeen Mohd Fauzi, Aziee Sudin; *University Malaya Medical Centre*: Soo Kun Lim, Chye Chung Gan, Albert Hing, Wan Ahmad Faizal Alaidin Razali; *Hospital Pulau Pinang*: Yew Fong Liew, Chelsia Bao Tyng Chan, Mei Chih Cheng, Yu Chen Ong, Loke Meng Ong, Farah Amalina Mohamed Affandi; *Hospital Melaka*: Korina Rahmat, Ban Chai Peng, Masayu Amat; *Hospital Pakar Sultanah Fatimah*: Nuzaimin Hadafi Ahmad, Doo Yee Mah, Yi Loon Tye, Zaid Azhari, Siti Nabilah Mohamad Zaini, Mohd Aidil Musa; *Hospital Ampang*: Norazinizah Ahmad Miswan, Rafizanur Ramli, Nor Aziah Ahmad; *Hospital Serdang*: Bak Leong Goh, Nurul Izah Ahmad, Fairol Huda Ibrahim, Tze Jian Ng, Malini Shanmuganathan, Li Lian Tay; *Hospital Sultanah Nur Zahirah*: Zaiha Harun, Salmi Ramli, Nurul 'Ain Yusof, Rossenizal Abd Rahman; *Pusat Perubatan UiTM*: Muhammad Iqbal Abdul Hafidz, Nur Hidayati Mohd Sharif, Irda Yasmoon Awang

Japan: *Chubu Rosai Hospital*: Eitaro Nakashima, Rui Imamine, Makiko Minatoguchi, Yukari Miura, Miduki Nakaoka, Yoshiki Suzuki, Hitomi Yoshikawa; *Shin Clinic*: Koki Shin, Kanae Fujita, Misuzu Iwasa, Haruka Sasajima, Airi Sato; *Kansai Electric Power Hospital*: Yoshiyuki Hamamoto, Yuki Fujita, Takuya Haraguchi, Takanori Hyo, Kiyohiro Izumi, Toshiyuki Komiya, Sodai Kubota, Takeshi Kurose, Hitoshi Kuwata, Susumu Nakatani, Kaori Oishi, Saki Okamoto, Kaori Okamura, Jun Takeoka, Nagaaki Tanaka, Katsuya Tanigaki, Naohiro Toda, Koin Watanabe, Hiromi Komori, Rika Kumuji, Asako Takesada, Aya Tanaka; *Nagoya University Hospital*: Shoichi Maruyama, Tomonori Hasegawa, Akiko Ishiguro, Takuji Ishimoto, Kazuhiro Ito, Yutaka Kamimura, Noritoshi Kato, Sawako Kato, Hiroshi Kojima, Tomoki Kosugi, Kayaho Maeda, Masasi Mizuno, Shoji Saito, Hitomi Sato, Yuka Sato, Yasuhiro Suzuki, Akihito Tanaka, Yoshinari Yasuda, Fujiko Hasegawa, Maiko Hayashi, Shizuka Higashi, Kaho Shimamura, Momoko Sumi, Kazuki Tajima, Chimaki Unekawa, Kana Wakayama, Yukiko Wakita; *Ota diabetes clinic*: Takatoshi Otani, Ayako Imai, Sayaka Kawashima, Eri Kogure, Tomoe Sato, Misato Takezawa, Shinya Yoshida; *Fukui Prefectural Hospital*: Hideo Araki, Yuko Katsuda, Masahiro Konishi, Takahiro Matsunaga, Masashi Oe, Kunihiro Ogane, Masato Sakai, Tomoko Takahashi, Takahiro Yamano, Takuya Yokoyama, Hitomi Ito, Masayo Katayama, Emi Kuroda; *Medical Corporation Seijinkai Ikeda Hospital*: Toru Ikeda, Takuma Kojo, Etsuo Yoshidome, Rieko Mizumachi, Akane Yamamoto, Narihisa Yamasaki, Yoshihiko Yamasaki; *Okayama University Hospital*: Jun Wada, Jun Eguchi, Chigusa Higuchi, Akihiro Katayama, Masaru Kinomura, Masashi Kitagawa, Shinji Kitamura, Satoshi Miyamoto, Hiroshi Morinaga, Atsuko Nakatsuka, Ichiro Nojima, Kenichi Shikata, Hitoshi Sugiyama,

Katsuyuki Tanabe, Kenji Tsuji, Haruhito Uchida, Mayu Watanabe, Chie Hashimoto, Takahiro Kato, Sayaka Yamamoto; *Tokai University Hospital*: Takehiko Wada, Masafumi Fukagawa, Naoto Hamano, Masahiro Koizumi, Hirotaka Komaba, Yosuke Nakagawa, Michiyo Iwamoto; *Fukuoka University Hospital*: Kosuke Masutani, Akane Katanosaka, Mayu Kiyota, Hikari Uchi, Yuka Ueda, Sonoka Yamamoto; *Kawasaki Medical School Hospital*: Hajime Nagasu, Seiji Itano, Tsukasa Iwakura, Hiroyuki Kadoya, Eiichiro Kanda, Naoki Kashihara, Kengo Kidokoro, Megumi Kondo, Tamaki Sasaki, Minoru Satoh, Atsuyuki Tokuyama, Reina Umeno, Yoshihisa Wada, Toshiya Yamamoto, Yu Yamanouchi, Masumi Abe, Yoko Inukai; *Kobe University Hospital*: Wataru Ogawa, Shunichiro Asahara, Hideki Fujii, Shunsuke Goto, Yushi Hirota, Tetsuya Hosooka, Keiji Kono, Shinichi Nishi, Yuko Okada, Kazuhiko Sakaguchi, Kenji Sugawara, Michiko Takahashi, Tomoko Takai, Yoshikazu Tamori, Kentaro Watanabe, Miyu Kitajima, Misaki Nishi, Junko Wada; *Aichi Medical University Hospital*: Yasuhiko Ito, Hideki Kamiya, Akimasa Asai, Nao Asai, Saeko Asano, Shogo Banno, Yohei Ejima, Hanako Hase, Tomohide Hayami, Tatsuhito Himeno, Takahiro Ishikawa, Mayumi Ito, Shiho Iwagaitsu, Rina Kasagi, Yoshiro Kato, Makoto Kato, Koichi Kato, Takayuki Katsuno, Miyuka Kawai, Hiroshi Kinashi, Masaki Kondo, Masako Koshino, Naoya Matsuoka, Yoshiaki Morishita, Mikio Motegi, Jiro Nakamura, Hiromi Shimoda, Hirokazu Sugiyama, Shin Tsunekawa, Makoto Yamaguchi, Kazuyo Takahashi; *Juntendo University Hospital*: Hirotaka Watada, Takashi Funayama, Yasuhiko Furukawa, Tomohito Gohda, Hiromasa Goto, Hideyoshi Kaga, Yasuhiko Kanaguchi, Akio Kanazawa, Kayo Kaneko, Toshiki Kano, Masao Kihara, Shogo Kimura, Takashi Kobayashi, Masayuki Maiguma, Yuko Makita, Satoshi Mano, Tomoya Mita, Takeshi Miyatsuka, Maki Murakoshi, Masahiro Muto, Masami Nakata, Junichiro Nakata, Yuya Nishida, Nao Nohara, Takeshi Ogihara, Daisuke Sato, Junko Sato, Hiroaki Sato, Yusuke Suzuki, Ruka Suzuki, Hitoshi Suzuki, Miyuki Takagi, Yoshifumi Tamura, Toyoyoshi Uchida, Seiji Ueda, Miki Asawa, Minako Miyaji, Eri Nagashima, Yoshie Shibata, Eri Yanagisawa; *The University of Tokyo School of Medicine/Toranomon Hospital*: Takashi Kadowaki, Toshimasa Yamauchi, Masaomi Nangaku, Yosuke Hirakawa, Hiroshi Nishi, Nobuhiro Shojima, Satoko Horikawa, Yukiko Nakayama, Naoko Yamada, Yuki Omori; *Maebashi Hirosegawa Clinic*: Shintaro Yano, Miyabi Ioka, Nahoko Kuwabara, Remi Nagano, Megumi Nozawa, Yumi Osawa; *Shiga University of Medical Science Hospital*: Hiroshi Maegawa, Shinji Kume, Shinichi Araki, Itsuko Miyazawa, Katsutaro Morino, Ikuko Kawai, Masumi Sobata, Motoko Takaoka; *Koukan Clinic*: Yasushi Iwaita, Takashi Udagawa, Ami Inamori, Aya Kawase, Aya Yamanaka; *University of Tsukuba Hospital*: Hitoshi Shimano, Akiko Fujita, Hitoshi Iwasaki, Hirayasu Kai, Yoshinori Osaki, Chie Saito, Motohiro Sekiya, Ryoya Tsunoda, Kunihiro Yamagata, Rikako Nakamura, Aiko Yamada; *Center Hospital of the National Center for Global Health and Medicine*: Mitsuru Ohsugi, Motoharu Awazawa, Ryotaro Bouchi, Shota Hashimoto, Makiko Hashimoto, Tomoko Hisatake, Noriko Ihana, Koko Ishizuka, Kazuo Izumi, Hiroshi Kajio, Michi Kobayashi, Noriko Kodani, Koji Maruyama, Michihiro Matsumoto, Maya Matsushita, Tomoka Nakamura, Takehiro Sugiyama, Akiyo Tanabe, Aiko Terakawa, Kojiro Ueki, Yuko Orimo, Takako Ozawa, Eriko Takahira; *AMC Nishi-Umeda Clinic*: Yoshimitsu Yamasaki, Masakazu Haneda, Tadahiro Tomita, Saori Akimoto, Akihiro Fujimoto, Kenji Ishihara, Chiho Murakami, Akiyo Nishiyama, Yukiko Toyonaga, Kana Uozumi, Yukihiro Yamaji; *Jyoumou Ohashi Clinic*: Tetsuya Shigehara, Jun Okajyo, Yukihiro Shimizu; *Iwasaki internal medicine clinic*: Shingo Iwasaki, Yuki Fukao, Megumi Furusho, Shintaro Nunokawa; *Tohoku University Hospital*: Hideki Katagiri, Tomohito Izumi, Keizo Kaneko, Shinjiro Kodama, Mariko Miyazaki, Yuichiro Munakata, Tasuku

Nagasawa, Yuji Oe, Hiroto Sugawara, Kei Takahashi, Kazushige Hirata, Keiko Inomata, Shoko Otomo, Taeko Uchida, Chigusa Yamashita; *Tokyo-eki Center-building Clinic*: Arihiro Kiyosue, Ryota Tamura

Canada: *CRIUCPQ*: Francois Dube, Marilene Bolduc, Marie-Christine Talbot; *University Health Network-Toronto General Hospital*: David Cherney, Leslie Cham, Vesta Lai, Josephine Tse; *Clinical Research Solutions Inc.*: Shivinder Jolly, Tabbatha Duck; *Interior Health Kelowna General Hospital*: Scott Lyle, Rachel Epp, Camille Galloway, Susan Haskett, Elizabeta Matvienko, Liam Paulsen; *London Health Sciences Centre*: Louise Moist, Kerri Gallo, Zabrina Lozon, Tina Ramsey, Brittany Whitmore; *St Paul's Hospital*: Adeera Levin, Bader Al-Zeer, Paula Macleod, Aoife O'Sullivan, Zainab Sheriff, Sam Tholl; *Cambridge Cardiac Care Centre*: Amritanshu Pandey, Samantha Armstrong, Bethelihem Gebeyehu, Patrick Toth; *LMC Clinical Research Inc. (Thornhill)*: Ronald Goldenberg, Mahsa Jahangiriesmaili, Shariff Sanguila, Neethi Suresh, Tanvi Talsania; *Vancouver General Hospital*: Nadia Zalunardo, Bader Al-Zeer, Paula Macleod, Aoife O'Sullivan, Zainab Sheriff; *CHU de Quebec-Universite Laval*: Mohsen Agharazii, Marie-Pier Roussel, Annie Saillant, France Samson; *LMC Clinical Research Inc. Brampton*: Harpreet Bajaj, Miken Bhavsar, Parul Dhall, Gagandeep Dhillon, Bhupinder Grewal, Taniya Nimbkar; *CIUSSS Nord de l'ile de Montreal*: Francois Madore, Guylaine Marcotte; *LMC Clinical Research Inc. (Bayview)*: Oren Steen, Mathura Bullen, Shayani Raguwaran, Andre Valleteau; *CIUSSS de l'Estrie-CHUS, Hopital Fleurimont*: Marie-France Langlois, Christine Brown; *Lakeridge Health*: Andrew Steele, Melissa Garrity, Taneera Ghate, Holly Robinson, Michael Tolibas; *LMC Clinical Research Inc. (Ottawa)*: Chetna Tailor, Lauren Elliott, Christine McClary-Wright; *Fadia El Boreky Medicine Professional*: Fadia Boreky, Sameh Fikry, Ayesha Ali, Chintankumar Barot, Wagdy Basily, Bethelihem Gebeyehu, Thisun Saram, Vinay Varad; *LMC Clinical Research Inc (Etobicoke)*: Hasnain Khandwala, Alex Aguilera, Patricia Alvarez, Balwinder Gill, Nazihah Huda, Aamir Navivala, Daniel Pinto; *Kidney Care Centre-Fraser Health*: Micheli Bevilacqua, Elaine Fung, Geraldine Hernandez, Puneet Mann, Jaskiran Saini; *Institut de recherches cliniques de Montreal*: Remi Rabasa-Lhoret, Danijela Bovan, Marie Devaux

Italy: *Policlinico San Martino, Genova*: Roberto Pontremoli, Cecilia Barnini, Giovanna Leoncini, Luca Manco, Giulia Nobili; *Ospedale Casa Sollievo della Sofferenza, San Giovanni Rotondo*: Matteo Piemontese, Filippo Aucella, Rachele Grifa, Francesco Totaro; *Policlinico S. Orsola-Malpighi, Bologna*: Gaetano La Manna, Irene Capelli, Giuseppe Cianciolo, Sarah Lerario, Fulvia Zappulo; *Ospedale S. Giovanni di Dio, Firenze*: Alberto Rosati, Filippo Fani, Giuseppe Spatoliatore, Ester Baldini, Francesca Bianchini; *AOU Policlinico, Bari*: Loreto Gesualdo, Francesco Pesce, Maria Russo, Maria Zippo, Cesira Cafiero; *Ospedale Martini, Torino*: Daria Motta, Simona Bianco, Donatella Bilucaglia; *Ospedale Maggiore Policlinico, Milano*: Piergiorgio Messa, Laura Pavone, Federica Tripodi, Simone Vettoretti; *AOU Padova*: Paola Fioretto, Gianni Carraro, Filippo Farnia, Anna Postal; *Ospedale Sacro Cuore di Gesù, Gallipoli*: Alessandro D'Amelio, Antonio Cardone, Giovanni Piccinni, Annalisa Aloisi; *ASST Spedali Civili, Brescia*: Francesco Scolari, Federico Alberici, Alice Guerini, Chiara Saccà, Chiara Salviani, Roberta Zani; *AOU L. Vanvitelli, Napoli*: Luca De Nicola, Carlo Garofalo, Maria Elena Liberti, Roberto Minutolo, Luigi Pennino, Lucio

Polese; AOU *Sant' Andrea, Roma*: Paolo Mené, Simona Barberi, Clorinda Falcone; *Ospedale Ignazio Veris delli Ponti, Scorrano*: Francesco Russo, Maurizio Caroppo; *Ospedale di Circolo, Desio*: Gennaro Santorelli, Rodolfo Rivera; AOU *Policlinico G. Martino, Messina*: Domenico Santoro, Alfio Giuffrida, Fortunata Zirino; *Ospedale Civile SS. Antonio e Biagio, Alessandria*: Cristina Calvi, Luca Estienne; *AOUI, Verona*: Giovanni Gambaro, Concetta Gangemi, Vittorio Ortalda, Giuseppina Pessolano; *Fondazione Policlinico Universitario Agostino Gemelli, Roma*: Giuseppe Grandaliano, Rocco Baccaro, Pietro Ferraro, Roberto Mangiacapra; *IRCCS Ospedale San Raffaele, Milano*: Marco Melandri, Nadia Foligno, Rita Quartagno, Giuseppe Vezzoli, Elena Brioni

Supplementary Methods

Definitions

Level of multimorbidity was established by the presence or absence of eight self-reported conditions at baseline: diabetes, heart failure, ischaemic heart disease [any history of myocardial infarction or angina], cerebrovascular disease [any history of stroke or transient ischaemic attack], peripheral arterial disease, atrial fibrillation, peripheral neuropathy and gout). CKD was not included in the level of multimorbidity since it determined eligibility (i.e. all participants had CKD).

Health-related quality of life at randomization was assessed using the EuroQoL EQ-5D-5L tool which requires participants to rate on an ordinal scale five domains (mobility, self-care, usual activities, pain or discomfort and anxiety or depression) from which an index value is derived; and additionally to rate their overall health on that day between zero and 100 using a visual analogue scale.

To enable subgroup analyses, participants were categorised into approximate thirds of predicted risk of hospitalization (reflecting frailty), multimorbidity, polypharmacy and health-related quality of life. The top third of hospitalization risk was further dichotomized to provide greater discrimination among those at highest risk. Categorisations were ≤20%, >20% ≤35%, >35% ≤45% and >45% for predicted risk of hospitalization; ≤1, 2 and ≥3 conditions (excluding CKD) to reflect multimorbidity; ≤5, ≥6 to <9 and ≥9 concomitant medications; and EQ-5D index value >0.987, >0.811 ≤0.987 and ≤0.811 (lower values indicate poorer quality of life).

Safety Outcomes

Safety outcomes were serious (defined as life-threatening or resulting in death, hospitalization, persistent or significant disability or incapacity) occurrences of urinary tract infection, genital infection, hyperkalaemia, acute kidney injury, hypoglycaemia; as well as any liver injury, ketoacidosis, lower limb amputation, bone fracture; and serious and symptomatic dehydration. Bone fractures and symptomatic dehydration are emphasised (in addition to ketoacidosis, a recognized risk of SGLT2 inhibitor therapy) in presentation of absolute risks as key vulnerabilities associated with frailty in CKD.

Supplementary Statistical Methods

Logistic regression models adjusted for age, sex and region assessed the individual association of all potential predictor variables with recorded hospitalization (first event). Missing data in predictor variables was handled by mean (or median for non-normal data) imputation (or the most common category for categorical variables) since missingness was infrequent and not associated with first hospitalization on logistic regression. For continuous variables, nonlinearity was assessed by comparing models with and without quadratic terms in addition to the linear terms using likelihood ratio tests. If the inclusion of the quadratic term improved model fit, both terms were included in multivariable model building. All variables which were significantly associated ($P<0.01$) with hospitalization in univariable (adjusted for age, sex and region) models proceeded to inclusion in multivariable model building using forward stepwise selection, adding variables in order of best-fitting univariable (adjusted for age, sex and region) models (determined by lowest Akaike information criterion

values). The impact of stepwise additions was assessed using likelihood ratio tests with significance threshold P<0.01. Model performance was assessed using calibration plots and the area under the receiver operating characteristic curve (AUROC) for all-cause hospitalization. The model developed with all-cause hospitalization as the response variable was also separately assessed using the AUROC with death from any cause as the response variable to test whether the identified predictors (of risk of hospitalization as an indicator of frailty) also had reasonable discrimination for death (which has established associations with clinical frailty).

Supplementary Tables & Figures

Supplementary Table 1: Variables assessed as potential predictors of hospitalization

Data item	Data type	Description
DEMOGRAPHICS		
Age, years	Continuous	Age at randomization; all models were adjusted for age
Sex	Binary	All models were adjusted for sex
Region	Categorical	All models were adjusted for region in the categories used in the minimised randomization algorithm (Europe, North America, Asian [China and Malaysia], Japan)
COMORBIDITIES		
Cardiovascular disease	Binary	Pre-specified variable defined as patient-reported history of myocardial infarction, heart failure, stroke, transient ischaemic attack, or peripheral arterial disease; not retained in model building favouring counting component conditions individually applying cumulative deficits principles
Heart failure	Binary	Patient-reported history at randomization
Myocardial infarction	Binary	Patient-reported history at randomization; combined with angina to derive ischaemic heart disease variable to be used in preference
Angina	Binary	Patient-reported history at randomization; combined with myocardial infarction to derive ischaemic heart disease variable to be used in preference
Stroke	Binary	Patient-reported history at randomization; combined with transient ischaemic attack to derive cerebrovascular disease variable to be used in preference
Transient ischaemic attack (TIA)	Binary	Patient-reported history at randomization; combined with stroke to derive cerebrovascular disease variable to be used in preference
Ischaemic heart disease	Binary	Derived variable defined as any history of either myocardial infarction or angina reported at randomization
Cerebrovascular disease	Binary	Derived variable defined as any history of either stroke or TIA reported at randomization
Peripheral arterial disease	Binary	Patient-reported history at randomization
Atrial fibrillation	Binary	Patient-reported history at randomization
Diabetes	Binary	Pre-specified variable defined as a patient-reported history of diabetes of any type, use of glucose-lowering medication, or a glycated haemoglobin level of at least 48 mmol per mole (6.5%) at the randomization visit
Diabetic retinopathy	Binary	Patient-reported history of diabetic retinopathy at randomization
Diabetes with retinopathy	Categorical	Participants with diabetes were further split into those reporting history of diabetic retinopathy and those not to derive a three-level variable reflecting disease severity (end-organ damage)
Peripheral neuropathy	Binary	Patient-reported history at randomization
Swollen ankles	Binary	Patient-reported history at randomization
Gout	Binary	Patient-reported history at randomization
CLINICAL MEASUREMENTS		
Body mass index, kg/m^2	Continuous	Weight in kilograms divided by the square of the height in meters at randomization
Waist:hip ratio	Continuous	Waist circumference in centimetres divided by hip circumference in centimetres at randomization
Systolic blood pressure, mmHg	Continuous	Measured at randomization; not retained in model building in favour of pulse pressure which was more strongly associated with hospitalization than either systolic or diastolic blood pressure
Diastolic blood pressure, mmHg	Continuous	
Pulse pressure, mmHg	Continuous	The difference between systolic and diastolic blood pressure (diastolic subtracted from systolic)
LABORATORY MEASUREMENTS		
Estimated glomerular filtration rate, ml/min/1.73 m^2	Continuous	Measurement recorded at the randomization visit or the most recent local laboratory result recorded before randomization
Urinary albumin-to-creatinine ratio, mg/g	Continuous	Measurement recorded at the randomization visit or the most recent local laboratory result recorded before randomization; log-transformed due to non-normal distribution; albumin was measured in milligrams and creatinine was measured in grams
N-terminal-pro B-type natriuretic peptide, ng/L	Continuous	Measurement recorded at the randomization visit; log-transformed due to non-normal distribution
Haemoglobin, mg/dL	Continuous	Measurement recorded at the randomization visit
HEALTH-RELATED QUALITY OF LIFE		
Mobility (EQ-5D)	Ordinal	Each domain was assessed separately at the randomization visit; mobility was strongly associated with hospitalization; the addition of other domains did not meaningfully add to model fit therefore only mobility was retained in the model building process (principle of parsimony)
Self-care (EQ-5D)	Ordinal	
Usual activities (EQ-5D)	Ordinal	
Pain/discomfort (EQ-5D)	Ordinal	
Anxiety/depression (EQ-5D)	Ordinal	
Self-rated health (EQ-5D visual analogue scale)	Continuous	Assessed at the randomization visit as part of the EQ-5D questionnaire; participants are asked to rate their overall health on the day between zero and 100 reflecting the worst and best health imaginable, respectively, using the visual analogue scale.
Exercise tolerance (NYHA)	Ordinal	Assessed at the randomization visit. The addition of exercise tolerance did not meaningfully add to model fit and overlaps with mobility therefore only mobility was retained in the model building process (principle of parsimony)

Supplementary Table 2: Univariable associations with hospitalization (logistic regression)

**CATEGORICAL & BINARY VARIABLES**									
	**ΔAIC***	**LRT statistic**	**LRT P**		**OR (95% CI)†**				
**Mobility**	-164.1	172.1	<0.001						
No problems					Ref				
Slight problems					1.76 (1.52-2.04)				
Moderate problems					2.41 (2.03-2.86)				
Severe problems					3.09 (2.40-3.99)				
Unable to walk about					4.45 (1.97-10.02)				
**Diabetes**	-106.0	110.0	<0.001						
No diabetes					Ref				
Diabetes without retinopathy					1.54 (1.36-1.73)				
Diabetes with retinopathy					2.40 (2.01-2.86)				
**Peripheral neuropathy**	-83.0	85.0	<0.001		1.86 (1.63-2.12)				
**Heart failure**	-74.9	76.9	<0.001		2.14 (1.81-2.53)				
**Ischaemic heart disease**	-62.5	64.5	<0.001		1.77 (1.54-2.04)				
**Self-reported ankle swelling**	-58.3	60.3	<0.001		1.64 (1.45-1.86)				
**Peripheral arterial disease**	-41.5	43.5	<0.001		1.94 (1.59-2.35)				
**Atrial fibrillation**	-29.6	31.6	<0.001		1.58 (1.35-1.86)				
**Cerebrovascular disease**	-28.2	30.2	<0.001		1.61 (1.36-1.90)				
**Gout**	0.6	1.4	0.240		1.08 (0.95-1.22)				
**CONTINUOUS VARIABLES**									
	**Linear terms only**					**Addition of quadratic terms**			
	**Improvement in fit**					**Improvement in fit**			
	**ΔAIC‡**	**LRT statistic**	**LRT P**	**Direction**	**OR (95% CI)†**	**ΔAIC§**	**LRT statistic**	**LRT P‖**	**Direction**
**Ln NT-proBNP**	-230.6	232.6	<0.001	+	1.40 (1.34-1.46)	-1.9	3.9	0.047	+
**EQ-5D visual analogue scale score**	-87.6	89.6	<0.001	-	0.98 (0.98-0.99)	1.2	0.8	0.360	-
**Haemoglobin**	-77.5	79.5	<0.001	-	0.98 (0.98-0.99)	-9.9	11.9	0.001	+
**eGFR**	-51.8	53.8	<0.001	-	0.98 (0.98-0.99)	-15.6	17.6	<0.001	+
**Body mass index**	-28.2	30.2	<0.001	+	1.02 (1.02-1.03)	2.0	0.0	0.923	-
**Pulse pressure**	-23.3	25.3	<0.001	+	1.01 (1.01-1.01)	1.8	0.2	0.669	+
**Waist:hip ratio**	-20.3	22.3	<0.001	+	3.25 (1.99-5.29)	-4.1	6.1	0.013	-
**Ln UACR**	-16.6	18.6	<0.001	+	1.07 (1.04-1.10)	-15.1	17.1	<0.001	+

* Improvement relative to model fitting age, sex and region only. † Adjusted for age, sex and region; for continuous variables the effect estimate is per one unit increment. ‡ Change in AIC for model including linear term for that predictor versus model fitting age, sex and region only. § Change in AIC for model with addition of quadratic term for that predictor versus model containing only linear term (adjusted for age, sex and region). ‖ Significant improvement in model fit at 1% threshold (P<0.01) used to determine inclusion of quadratic term. Missing values were infrequent and imputed with the mean (normally-distributed continuous variables), median (non-normally-distributed continuous variables) or mode (categorical variables). Abbreviations: AIC = Akaike information criterion; LRT = likelihood ratio test; eGFR = estimated glomerular filtration rate; Ln = natural logarithm; UACR = urinary albumin-to-creatinine ratio; NT-proBNP = N-terminal-pro B-type natriuretic peptide.

Supplementary Table 3: Incremental impact of each variable in the final multivariable logistic regression model to predict hospitalization

Model	AIC	ΔAIC	LRT statistic	LRT P value
Age, sex & region only	7870.0	NA	NA	NA
plus Ln NT-proBNP	7639.4	-230.6	232.6	<0.001
plus Mobility	7530.0	-109.3	117.3	<0.001
plus Diabetes	7479.8	-50.2	54.2	<0.001
plus Peripheral neuropathy	7462.9	-17.0	19.0	<0.001
plus Heart failure	7453.3	-9.5	11.5	<0.001
plus eGFR	7434.3	-19.0	23.0	<0.001
plus Ischaemic heart disease	7425.6	-8.8	10.8	0.001
plus Self-reported ankle swelling	7419.9	-5.7	7.7	0.006

Variables were sequentially added in order of smallest AIC (as an indicator of better fit) in models adjusted for age, sex & region only without adjustment for other characteristics. ΔAIC is the change in AIC with the addition of each variable relative to the model in the previous step with one fewer variable. Likelihood ratio tests also compare the addition of each variable to the model in the previous step with one fewer variable. The model adding eGFR includes linear and quadratic eGFR terms. Missing values were infrequent and imputed with the mean (normally-distributed continuous variables), median (non-normally-distributed continuous variables) or mode (categorical variables). Abbreviations: AIC = Akaike information criterion; LRT = likelihood ratio test; Ln = natural logarithm; NT-proBNP = N-terminal-pro B-type natriuretic peptide; eGFR = estimated glomerular filtration rate.

Supplementary Table 4: Other characteristics of participants at recruitment by predicted risk of hospitalization

	Predicted risk of hospitalization during follow-up (median 2 years)				
	≤20% (N=1988)	>20% ≤35% (N=2504)	>35% ≤45% (N=968)	>45% (N=1149)	P
**DEMOGRAPHICS**					
**Age at randomization (years)**					
Mean (SD)	52.8 (13.8)	65.6 (11.5)	71.4 (9.0)	72.6 (8.8)	<0.001
Category					<0.001
<60	1355 (68.2)	698 (27.9)	100 (10.3)	99 (8.6)	
≥60 <70	381 (19.2)	765 (30.6)	272 (28.1)	302 (26.3)	
≥70 <80	225 (11.3)	880 (35.1)	458 (47.3)	527 (45.9)	
≥80	27 (1.4)	161 (6.4)	138 (14.3)	221 (19.2)	
**Race (all regions)**					<0.001
White	930 (46.8)	1435 (57.3)	624 (64.5)	870 (75.7)	
Black	99 (5.0)	96 (3.8)	36 (3.7)	31 (2.7)	
Asian	920 (46.3)	942 (37.6)	295 (30.5)	236 (20.5)	
Mixed	9 (0.5)	7 (0.3)	2 (0.2)	3 (0.3)	
Other	30 (1.5)	24 (1.0)	11 (1.1)	9 (0.8)	
**PRIOR DISEASE**					
**History of heart failure**					<0.001
Yes	9 (0.5)	103 (4.1)	130 (13.4)	416 (36.2)	
No or missing	1979 (99.5)	2401 (95.9)	838 (86.6)	733 (63.8)	
**History of ischaemic heart disease***					<0.001
Yes	39 (2.0)	276 (11.0)	254 (26.2)	526 (45.8)	
No	1949 (98.0)	2228 (89.0)	714 (73.8)	623 (54.2)	
**History of peripheral arterial disease**					<0.001
Yes	23 (1.2)	121 (4.8)	91 (9.4)	235 (20.5)	
No	1965 (98.8)	2383 (95.2)	877 (90.6)	914 (79.5)	
**History of peripheral neuropathy**					<0.001
Yes	73 (3.7)	403 (16.1)	299 (30.9)	541 (47.1)	
No	1915 (96.3)	2101 (83.9)	669 (69.1)	608 (52.9)	
**Self-reported ankle swelling**					<0.001
Yes	146 (7.3)	476 (19.0)	335 (34.6)	559 (48.7)	
No	1842 (92.7)	2028 (81.0)	633 (65.4)	590 (51.3)	
**CLINICAL MEASUREMENTS**					
**Systolic blood pressure (mmHg)**					
Mean (SD)	132 (15)	138 (18)	140 (19)	138 (20)	<0.001
Category					<0.001
<130	925 (46.5)	802 (32.0)	278 (28.7)	393 (34.2)	
≥130 <145	678 (34.1)	836 (33.4)	301 (31.1)	374 (32.6)	
≥145	385 (19.4)	866 (34.6)	389 (40.2)	382 (33.2)	
**Diastolic blood pressure (mmHg)**					
Mean (SD)	82 (11)	79 (12)	75 (11)	73 (12)	<0.001
Category					<0.001
<75	503 (25.3)	913 (36.5)	499 (51.5)	665 (57.9)	
≥75 <85	678 (34.1)	820 (32.7)	265 (27.4)	289 (25.2)	
≥85	807 (40.6)	771 (30.8)	204 (21.1)	195 (17.0)	
**Pulse pressure (mmHg)**	49.8 (12.8)	59.6 (16.1)	65.5 (16.8)	65.1 (18.4)	<0.001
**Body mass index (kg/m^2)**					
Mean (SD)	28.3 (6.3)	29.4 (6.5)	30.8 (7.0)	32.1 (7.1)	<0.001
Category					<0.001
<25	620 (31.2)	662 (26.4)	172 (17.8)	165 (14.4)	
≥25 <30	746 (37.5)	877 (35.0)	340 (35.1)	334 (29.1)	
≥30	621 (31.2)	961 (38.4)	453 (46.8)	642 (55.9)	
Missing	1 (0.1)	4 (0.2)	3 (0.3)	8 (0.7)	
**Waist-to-hip ratio**	0.9 (0.1)	1.0 (0.1)	1.0 (0.1)	1.0 (0.1)	<0.001
**LABORATORY MEASUREMENTS**					
**Estimated GFR (mL/min/1.73m^2)**					
Mean (SD)	45.1 (15.6)	36.2 (13.7)	33.1 (11.2)	30.0 (9.3)	<0.001
Category					<0.001
<30	275 (13.8)	898 (35.9)	444 (45.9)	665 (57.9)	
≥30 <45	915 (46.0)	1178 (47.0)	413 (42.7)	422 (36.7)	
≥45	798 (40.1)	428 (17.1)	111 (11.5)	62 (5.4)	

**Urinary albumin-to-creatinine ratio (mg/g)**					<0.001
Geometric mean (95% CI)	299 (277-323)	210 (194-227)	177 (154-202)	183 (162-206)	
Median (Q1-Q3)	440 (133-1056)	314 (43-1062)	220 (29-1060)	193 (34-1118)	
Category					<0.001
<30	279 (14.0)	543 (21.7)	245 (25.3)	261 (22.7)	
≥30 ≤300	507 (25.5)	692 (27.6)	281 (29.0)	384 (33.4)	
>300	1202 (60.5)	1269 (50.7)	442 (45.7)	504 (43.9)	
**Glycated haemoglobin (mmol/mol)**					
Mean (SD)	39.1 (10.3)	45.4 (13.6)	48.8 (13.7)	51.1 (14.6)	<0.001
Category					<0.001
<39	1284 (64.6)	953 (38.1)	240 (24.8)	205 (17.8)	
≥39 <48	463 (23.3)	729 (29.1)	291 (30.1)	354 (30.8)	
≥48 <75	168 (8.5)	688 (27.5)	366 (37.8)	502 (43.7)	
≥75	36 (1.8)	89 (3.6)	47 (4.9)	80 (7.0)	
Missing	37 (1.9)	45 (1.8)	24 (2.5)	8 (0.7)	
**NT-proBNP (ng/L)**					<0.001
Geometric mean (95% CI)	48 (46-50)	164 (158-171)	369 (346-393)	846 (792-903)	
Median (Q1-Q3)	52 (15-98)	160 (90-302)	356 (181-714)	851 (369-1865)	
Category					<0.001
<110	1552 (78.1)	821 (32.8)	104 (10.7)	33 (2.9)	
≥110 <330	382 (19.2)	1109 (44.3)	348 (36.0)	222 (19.3)	
≥330	37 (1.9)	542 (21.6)	507 (52.4)	890 (77.5)	
Missing	17 (0.9)	32 (1.3)	9 (0.9)	4 (0.3)	
**Haematocrit (%)**					
Mean (SD)	40.8 (4.9)	39.0 (4.9)	37.8 (4.9)	37.4 (5.3)	<0.001
Category					<0.001
<37%	331 (16.6)	688 (27.5)	332 (34.3)	467 (40.6)	
≥37 <41%	538 (27.1)	735 (29.4)	301 (31.1)	317 (27.6)	
≥41%	948 (47.7)	817 (32.6)	232 (24.0)	254 (22.1)	
Missing	171 (8.6)	264 (10.5)	103 (10.6)	111 (9.7)	
**KDIGO risk category**					<0.001
Low, moderate or high	678 (34.1)	640 (25.6)	197 (20.4)	157 (13.7)	
Very high	1310 (65.9)	1864 (74.4)	771 (79.6)	992 (86.3)	
**CONCOMITANT MEDICATION USE**					
Any diuretic	507 (25.5)	925 (36.9)	548 (56.6)	835 (72.7)	<0.001
Loop diuretic	164 (8.2)	474 (18.9)	385 (39.8)	724 (63.0)	<0.001
Thiazide diuretic	287 (14.4)	434 (17.3)	202 (20.9)	199 (17.3)	<0.001
Mineralocorticoid receptor antagonist	105 (5.3)	132 (5.3)	86 (8.9)	152 (13.2)	<0.001
Potassium sparing & other	12 (0.6)	13 (0.5)	9 (0.9)	4 (0.3)	0.343
Beta blocker	404 (20.3)	976 (39.0)	573 (59.2)	808 (70.3)	<0.001
Anticoagulant	20 (1.0)	81 (3.2)	69 (7.1)	146 (12.7)	<0.001
Antiplatelet therapy	308 (15.5)	809 (32.3)	476 (49.2)	646 (56.2)	<0.001
Diabetes treatment	292 (14.7)	1037 (41.4)	561 (58.0)	805 (70.1)	<0.001
Biguanide (e.g. metformin)	103 (5.2)	308 (12.3)	125 (12.9)	133 (11.6)	<0.001
Sulfonylurea	85 (4.3)	252 (10.1)	123 (12.7)	125 (10.9)	<0.001
Insulin	145 (7.3)	590 (23.6)	357 (36.9)	571 (49.7)	<0.001
DPP-4 inhibitor	74 (3.7)	345 (13.8)	186 (19.2)	277 (24.1)	<0.001
GLP-1 agonist	44 (2.2)	142 (5.7)	71 (7.3)	80 (7.0)	<0.001
Other antidiabetic agent	34 (1.7)	127 (5.1)	80 (8.3)	73 (6.4)	<0.001

Figures are n (%) or mean (SD) or median (Q1-Q3). Predicted risk of hospitalization was derived from multivariable logistic regression models adjusted for age, sex and region assessing the association of all potential predictor variables with recorded hospitalization (first event; see Supplementary Methods). * Defined as self-reported history of myocardial infarction or angina. P values are from Chi squared tests for categorical variables; one-way ANOVA for normally distributed and Kruskal-Wallis tests for non-normally distributed continuous variables, respectively. Abbreviations: NT-proBNP = N-terminal pro B-type natriuretic peptide; DPP-4 = dipeptidyl peptidase-4; GLP-1 = glucagon-like peptide-1.

Supplementary Table 5: Composition of multimorbidity subgroups

	No. of conditions (excluding CKD)			
	**None or one (N=3864)**	**Two (N=1369)**	**Three or more (N=1376)**	**Overall (N=6609)**
**Diabetes***	923 (23.9)	982 (71.7)	1135 (82.5)	3040 (46.0)
**Heart failure**	20 (0.5)	98 (7.2)	540 (39.2)	658 (10.0)
**Ischaemic heart disease†**	108 (2.8)	276 (20.2)	711 (51.7)	1095 (16.6)
**Cerebrovascular disease‡**	94 (2.4)	183 (13.4)	386 (28.1)	663 (10.0)
**Atrial fibrillation**	88 (2.3)	187 (13.7)	510 (37.1)	785 (11.9)
**Peripheral arterial disease**	31 (0.8)	100 (7.3)	339 (24.6)	470 (7.1)
**Peripheral neuropathy**	102 (2.6)	459 (33.5)	755 (54.9)	1316 (19.9)
**Gout**	564 (14.6)	453 (33.1)	690 (50.1)	1707 (25.8)

Data are n (%) of participants in each category of "No. of conditions (excluding CKD)" with that condition. All diagnoses are based on patient reports at baseline.
* Defined as patient-reported history of diabetes of any type, use of glucose-lowering medication, or a glycated haemoglobin level of at least 48 mmol per mole (6.5%) at the randomization visit.
† Defined as any patient-reported history of myocardial infarction or angina at baseline.
‡ Defined as any patient-reported history of stroke or transient ischaemic attack at baseline.

Supplementary Table 6: Number of participants who had discontinued randomised treatment at the end of follow-up and reasons for discontinuation according to (i) predicted risk of hospitalization; and (ii) number of concomitant medications.

**(i) PREDICTED RISK OF HOSPITALIZATION**						
			**EMPAGLIFLOZIN**			
	**≤20%**	**>20% ≤35%**	**>35% ≤45%**	**>45%**	**Total**	**P***
	**N=983**	**N=1245**	**N=487**	**N=589**	**N=3304**	
**Any reason†**	**139 (14.1%)**	**198 (15.9%)**	**93 (19.1%)**	**127 (21.6%)**	**557 (16.9%)**	<0.001
SAE	11	22	8	18	**59**	
NSAE	14	20	8	16	**58**	
Other	54	74	41	57	**226**	
Unknown	60	82	36	36	**214**	
			**PLACEBO**			
	**≤20%**	**>20% ≤35%**	**>35% ≤45%**	**>45%**	**Total**	**P***
	**N=1005**	**N=1259**	**N=481**	**N=560**	**N=3305**	
**Any reason†**	**147 (14.6%)**	**237 (18.8%)**	**99 (20.6%)**	**157 (28.0%)**	**640 (19.4%)**	<0.001
SAE	10	30	11	24	**75**	
NSAE	7	18	7	10	**42**	
Other	58	97	48	65	**268**	
Unknown	72	92	33	58	**255**	

**(ii) NUMBER OF CONCOMITANT MEDICATIONS**					
		**EMPAGLIFLOZIN**			
	**<5**	**≥6 <9**	**≥9**	**Total**	**P***
	**N=1128**	**N=1010**	**N=1166**	**N=3304**	
**Any reason†**	**178 (15.7%)**	**145 (14.4%)**	**234 (20.1%)**	**557 (16.9%)**	<0.001
SAE	17	15	27	**59**	
NSAE	17	15	26	**58**	
Other	72	55	99	**226**	
Unknown	72	60	82	**214**	
		**PLACEBO**			
	**<5**	**≥6 <9**	**≥9**	**Total**	**P***
	**N=1121**	**N=1004**	**N=1180**	**N=3305**	0.004
**Any reason†**	**188 (16.8%)**	**190 (18.9%)**	**262 (22.2%)**	**640 (19.4%)**	
SAE	16	15	44	**75**	
NSAE	11	9	22	**42**	
Other	65	88	115	**268**	
Unknown	96	78	81	**255**	

Predicted risk of hospitalization during follow-up (median 2 years) was derived from multivariable logistic regression models adjusted for age, sex and region assessing the association of all potential predictor variables with recorded hospitalization (first event; see Supplementary Methods). * P value from Chi squared test comparing proportion discontinuing treatment for any reason for across categories, separately for the empagliflozin and placebo groups. † Number (%) of participants who had discontinued study treatment at the end of follow-up out of all randomised participants in that subgroup. Abbreviations: SAE = serious adverse event; NSAE = non-serious adverse event. All SAE, NSAE and Other reasons for discontinuation of study treatment are listed in a previous publication (The EMPA-KIDNEY Collaborative Group. Empagliflozin in Patients with Chronic Kidney Disease. *N Engl J Med.* 2023; Table S3).

Supplementary Table 7: Primary and secondary outcomes by predicted risk of hospitalization

Predicted risk of hospitalization (%)	Empagliflozin		Placebo		Relative effects		Estimated absolute effects*	
	n/N	Rate per 1000 patient-years	n/N	Rate per 1000 patient-years	Hazard Ratio (95% CI)	P_{het}	Events avoided per 1000 patient-years (SE)	P_{trend}
**PRIMARY OUTCOME AND ITS COMPONENTS**								
**Primary outcome: progression of kidney disease or death from cardiovascular causes**						0.60		<0.001
≤20%	68/983	36.7	93/1005	50.0	0.77 (0.56-1.06)		13.8 (2.3)	
>20% ≤35%	159/1245	67.1	218/1259	90.9	0.65 (0.53-0.80)		25.1 (4.2)	
>35% ≤45%	73/487	77.3	102/481	111.3	0.66 (0.49-0.90)		30.7 (5.2)	
>45%	132/589	116.4	145/560	137.4	0.79 (0.62-1.00)		37.9 (6.4)	
**Overall**	**432/3304**	**68.5**	**558/3305**	**89.6**	**0.72 (0.64-0.82)**			
**KEY SECONDARY OUTCOMES**								
**Hospitalization for heart failure or death from cardiovascular causes**						0.27		0.01
≤20%	3/983	1.6	1/1005	0.5	‡		0.1 (0.1)	
>20% ≤35%	16/1245	6.6	31/1259	12.4	0.53 (0.29-0.96)		1.9 (1.3)	
>35% ≤45%	22/487	22.6	22/481	23.1	1.02 (0.56-1.84)		3.6 (2.3)	
>45%	90/589	78.9	98/560	92.5	0.85 (0.64-1.13)		14.5 (9.3)	
**Overall**	**131/3304**	**20.4**	**152/3305**	**23.7**	**0.84 (0.67-1.07)**			
**Hospitalization for any cause (first and all subsequent events)**						0.63		<0.001
≤20%	186	99	221	116	0.86 (0.68-1.07)		16.4 (5.1)	
>20% ≤35%	501	206	577	230	0.91 (0.77-1.06)		32.3 (10.0)	
>35% ≤45%	327	332	395	410	0.79 (0.63-0.98)		57.6 (17.8)	
>45%	597	502	702	627	0.78 (0.65-0.95)		88.1 (27.3)	
**Overall**	**1611**	**248**	**1895**	**292**	**0.86 (0.78-0.95)**			
**Death from any cause**						0.48		0.03
≤20%	4/983	2.1	3/1005	1.6	‡		0.2 (0.2)	
>20% ≤35%	35/1245	14.4	41/1259	16.4	0.87 (0.56-1.37)		2.1 (1.6)	
>35% ≤45%	23/487	23.3	37/481	38.4	0.61 (0.36-1.02)		5.0 (3.8)	
>45%	86/589	72.3	86/560	76.8	0.95 (0.71-1.29)		10.1 (7.5)	
**Overall**	**148/3304**	**22.8**	**167/3305**	**25.8**	**0.87 (0.70-1.08)**			
**OTHER SECONDARY OUTCOMES**								
**Any kidney disease progression**						0.76		<0.001
≤20%	67/983	36.1	93/1005	50.0	0.77 (0.56-1.06)		14.6 (2.4)	
>20% ≤35%	150/1245	63.3	204/1259	85.1	0.65 (0.52-0.80)		24.8 (4.1)	
>35% ≤45%	66/487	69.9	90/481	98.2	0.66 (0.48-0.91)		28.6 (4.7)	
>45%	101/589	89.1	117/560	110.8	0.74 (0.57-0.97)		32.3 (5.3)	
**Overall**	**384/3304**	**60.9**	**504/3305**	**80.9**	**0.71 (0.62-0.81)**			
**Death from cardiovascular causes**						0.63§		-
≤20%	1/983	0.5	0/1005	0.0	‡		0.0 (0.0)	
>20% ≤35%	9/1245	3.7	15/1259	6.0	0.62 (0.27-1.41)		0.9 (0.9)	
>35% ≤45%	9/487	9.1	13/481	13.5	0.68 (0.29-1.59)		2.1 (2.0)	
>45%	40/589	33.6	41/560	36.6	0.92 (0.60-1.43)		5.7 (5.5)	
**Overall**	**59/3304**	**9.1**	**69/3305**	**10.6**	**0.84 (0.60-1.19)**			
**ESKD or death from cardiovascular causes †**						0.09		<0.001
≤20%	17/983	9.1	15/1005	7.9	1.23 (0.61-2.46)		2.2 (0.6)	
>20% ≤35%	46/1245	19.1	79/1259	32.1	0.55 (0.38-0.79)		8.7 (2.4)	
>35% ≤45%	25/487	25.7	42/481	44.5	0.58 (0.35-0.95)		12.1 (3.4)	
>45%	75/589	64.7	81/560	74.5	0.86 (0.62-1.17)		20.2 (5.6)	
**Overall**	**163/3304**	**25.4**	**217/3305**	**34.0**	**0.73 (0.59-0.89)**			

Predicted risk of hospitalization during follow-up (median 2 years) was derived from multivariable logistic regression models adjusted for age, sex and region assessing the association of all potential predictor variables with recorded hospitalization (first event; see Supplementary Methods). The p values shown are standard tests for heterogeneity or trend across categories of predicted risk of hospitalization for the relative and estimated absolute effects; respectively. * Absolute events avoided per 1000 patients treated with empagliflozin for 1 year (SE) were estimated by applying the overall hazard ratio (or 95% CI) to the subgroup-specific event rate per 1000 patient-years in the placebo group. † ESKD: End-Stage Kidney Disease, defined as start of maintenance dialysis or receipt of a kidney transplant. ‡ Hazard ratios are not presented for outcomes with fewer than 10 events. § Heterogeneity test compares >20% ≤35%, >35% ≤45% and >45% since event numbers precluded reliable hazard ratio estimation for ≤20%; all other P_{het} refer to comparisons across all 4 levels of predicted risk of hospitalization.

Supplementary Table 8: Primary and secondary outcomes by multimorbidity

No. of conditions (excluding CKD)	Empagliflozin		Placebo		Relative effects		Estimated absolute effects*	
	n/N	Rate per 1000 patient-years	n/N	Rate per 1000 patient-years	Hazard Ratio (95% CI)	P_{het}	Events avoided per 1000 patient-years (SE)	P_{trend}
**PRIMARY OUTCOME AND ITS COMPONENTS**								
**Primary outcome: progression of kidney disease or death from cardiovascular causes**						0.38		0.33
≤1	233/1924	64.4	300/1940	83.5	0.71 (0.60-0.85)		23.1 (3.9)	
2	104/706	75.6	113/663	88.1	0.85 (0.65-1.11)		24.3 (4.1)	
≥3	95/674	72.4	145/702	106.9	0.66 (0.51-0.85)		29.5 (5.0)	
**Overall**	**432/3304**	**68.5**	**558/3305**	**89.6**	**0.72 (0.64-0.82)**			
**KEY SECONDARY OUTCOMES**								
**Hospitalization for heart failure or death from cardiovascular causes**						0.70		0.09
≤1	21/1924	5.6	29/1940	7.8	0.71 (0.41-1.25)		1.2 (0.8)	
2	37/706	26.3	36/663	27.4	0.98 (0.62-1.55)		4.3 (2.8)	
≥3	73/674	56.1	87/702	63.9	0.86 (0.63-1.17)		10.0 (6.4)	
**Overall**	**131/3304**	**20.4**	**152/3305**	**23.7**	**0.84 (0.67-1.07)**			
**Hospitalization for any cause (first and all subsequent events)**						0.78		0.44
≤1	638	290	728	333	0.89 (0.77-1.03)		46.8 (14.5)	
2	426	299	486	363	0.83 (0.68-1.01)		51.0 (15.8)	
≥3	547	407	681	483	0.83 (0.69-1.00)		67.8 (21.0)	
Overall	**1611**	**248**	**1895**	**292**	**0.86 (0.78-0.95)**			
**Death from any cause**						0.86		0.25
≤1	41/1924	11.0	48/1940	12.8	0.84 (0.55-1.27)		1.7 (1.3)	
2	40/706	28.1	38/663	28.4	0.98 (0.63-1.53)		3.7 (2.8)	
≥3	67/674	49.9	81/702	57.4	0.86 (0.62-1.19)		7.5 (5.6)	
**Overall**	**148/3304**	**22.8**	**167/3305**	**25.8**	**0.87 (0.70-1.08)**			
**OTHER SECONDARY OUTCOMES**								
**Any kidney disease progression**						0.59		0.92
≤1	223/1924	61.7	287/1940	79.9	0.71 (0.59-0.84)		23.3 (3.8)	
2	91/706	66.2	106/663	82.7	0.79 (0.60-1.04)		24.1 (4.0)	
≥3	70/674	53.4	111/702	81.8	0.63 (0.47-0.86)		23.8 (3.9)	
**Overall**	**384/3304**	**60.9**	**504/3305**	**80.9**	**0.71 (0.62-0.81)**			
**Death from cardiovascular causes**						0.44		0.36
≤1	12/1924	3.2	13/1940	3.5	0.91 (0.41-1.99)		0.5 (0.5)	
2	15/706	10.5	11/663	8.2	1.31 (0.60-2.85)		1.3 (1.2)	
≥3	32/674	23.8	45/702	31.9	0.73 (0.46-1.15)		5.0 (4.8)	
**Overall**	**59/3304**	**9.1**	**69/3305**	**10.6**	**0.84 (0.60-1.19)**			
**ESKD or death from cardiovascular causes**†						0.49		0.08
≤1	74/1924	20.1	94/1940	25.5	0.74 (0.54-1.00)		6.9 (1.9)	
2	41/706	29.1	45/663	34.3	0.90 (0.59-1.38)		9.3 (2.6)	
≥3	48/674	36.1	78/702	56.2	0.64 (0.45-0.92)		15.2 (4.3)	
**Overall**	**163/3304**	**25.4**	**217/3305**	**34.0**	**0.73 (0.59-0.89)**			

Multimorbidity was determined based on the presence/absence of 8 comorbidities at randomization (see Supplementary Table 5) excluding chronic kidney disease. The p values shown are standard tests for heterogeneity or trend across categories of comorbidities for the relative and estimated absolute effects; respectively. Hazard ratios are not presented for outcomes with fewer than 10 events. * Absolute events avoided per 1000 patients treated with empagliflozin for 1 year (SE) were estimated by applying the overall hazard ratio (or 95% CI) to the subgroup-specific event rate per 1000 patient-years in the placebo group. † ESKD: End-Stage Kidney Disease, defined as start of maintenance dialysis or receipt of a kidney transplant.

Supplementary Table 9: Primary and secondary outcomes by concomitant medication count

No. of concomitant medications	Empagliflozin		Placebo		Relative effects		Estimated absolute effects*	
	n/N	Rate per 1000 patient-years	n/N	Rate per 1000 patient-years	Hazard Ratio (95% CI)	P_{het}	Events avoided per 1000 patient-years (SE)	P_{trend}
**PRIMARY OUTCOME AND ITS COMPONENTS**								
**Primary outcome: progression of kidney disease or death from cardiovascular causes**						0.16		0.08
≤5	133/1128	62.1	144/1121	68.0	0.88 (0.69-1.11)		18.8 (3.2)	
≥6 <9	134/1010	69.4	192/1004	102.1	0.67 (0.54-0.83)		28.2 (4.7)	
≥9	165/1166	74.0	222/1180	99.5	0.67 (0.55-0.82)		27.5 (4.6)	
**Overall**	**432/3304**	**68.5**	**558/3305**	**89.6**	**0.72 (0.64-0.82)**			
**KEY SECONDARY OUTCOMES**								
**Hospitalization for heart failure or death from cardiovascular causes**						0.26		0.13
≤5	7/1128	3.2	14/1121	6.4	0.48 (0.19-1.19)		1.0 (0.6)	
≥6 <9	38/1010	19.3	35/1004	17.9	1.08 (0.68-1.72)		2.8 (1.8)	
≥9	86/1166	38.0	103/1180	45.3	0.82 (0.62-1.09)		7.1 (4.6)	
**Overall**	**131/3304**	**20.4**	**152/3305**	**23.7**	**0.84 (0.67-1.07)**			
**Hospitalization for any cause (first and all subsequent events)**						0.05		0.04
≤5	353	160	335	153	1.06 (0.87-1.28)		21.5 (6.6)	
≥6 <9	444	202	568	258	0.77 (0.65-0.92)		36.2 (11.2)	
≥9	814	354	992	425	0.83 (0.71-0.96)		59.6 (18.5)	
**Overall**	**1611**	**248**	**1895**	**292**	**0.86 (0.78-0.95)**			
**Death from any cause**						0.75		0.22
≤5	19/1128	8.6	19/1121	8.7	0.96 (0.51-1.81)		1.1 (0.9)	
≥6 <9	43/1010	21.6	43/1004	21.9	0.98 (0.64-1.49)		2.9 (2.1)	
≥9	86/1166	37.4	105/1180	45.0	0.82 (0.61-1.09)		5.9 (4.4)	
**Overall**	**148/3304**	**22.8**	**167/3305**	**25.8**	**0.87 (0.70-1.08)**			
**OTHER SECONDARY OUTCOMES**								
**Any kidney disease progression**						0.08		0.22
≤5	128/1128	59.7	138/1121	65.2	0.89 (0.70-1.13)		19.0 (3.1)	
≥6 <9	122/1010	63.2	178/1004	94.6	0.66 (0.52-0.83)		27.6 (4.6)	
≥9	134/1166	60.1	188/1180	84.3	0.63 (0.50-0.78)		24.5 (4.1)	
**Overall**	**384/3304**	**60.9**	**504/3305**	**80.9**	**0.71 (0.62-0.81)**			
**Death from cardiovascular causes**						0.81		0.33
≤5	5/1128	2.3	8/1121	3.7	0.61 (0.20-1.86)		0.6 (0.5)	
≥6 <9	16/1010	8.0	19/1004	9.7	0.82 (0.42-1.60)		1.5 (1.4)	
≥9	38/1166	16.5	42/1180	18.0	0.90 (0.58-1.40)		2.8 (2.7)	
**Overall**	**59/3304**	**9.1**	**69/3305**	**10.6**	**0.84 (0.60-1.19)**			
**ESKD or death from cardiovascular causes**[†]						0.53		0.02
≤5	37/1128	16.9	40/1121	18.5	0.87 (0.56-1.37)		5.0 (1.4)	
≥6 <9	46/1010	23.4	74/1004	38.3	0.63 (0.44-0.91)		10.4 (2.9)	
≥9	80/1166	35.3	103/1180	45.0	0.75 (0.56-1.00)		12.2 (3.4)	
**Overall**	**163/3304**	**25.4**	**217/3305**	**34.0**	**0.73 (0.59-0.89)**			

The p values shown are standard tests for heterogeneity or trend across categories of concomitant medications for the relative and estimated absolute effects; respectively. Hazard ratios are not presented for outcomes with fewer than 10 events. * Absolute events avoided per 1000 patients treated with empagliflozin for 1 year (SE) were estimated by applying the overall hazard ratio (or 95% CI) to the subgroup-specific event rate per 1000 patient-years in the placebo group. † ESKD: End-Stage Kidney Disease, defined as start of maintenance dialysis or receipt of a kidney transplant.

Supplementary Table 10: Primary and secondary outcomes by health-related quality of life (EQ-5D index value)

EQ-5D index value	Empagliflozin		Placebo		Relative effects		Estimated absolute effects*	
	n/N	Rate per 1000 patient-years	n/N	Rate per 1000 patient-years	Hazard Ratio (95% CI)	P_{het}	Events avoided per 1000 patient-years (SE)	P_{trend}
**PRIMARY OUTCOME AND ITS COMPONENTS**								
**Primary outcome: progression of kidney disease or death from cardiovascular causes**						0.73		0.64
>0.987	126/1064	63.3	169/1083	83.2	0.78 (0.62-0.98)		23.0 (3.9)	
>0.811 ≤0.987	151/1117	71.1	196/1142	92.5	0.70 (0.57-0.87)		25.5 (4.3)	
≤0.811	155/1123	70.8	193/1080	92.8	0.69 (0.56-0.86)		25.6 (4.3)	
**Overall**	**432/3304**	**68.5**	**558/3305**	**89.6**	**0.72 (0.64-0.82)**			
**KEY SECONDARY OUTCOMES**								
**Hospitalization for heart failure or death from cardiovascular causes**						0.21		0.45
>0.987	21/1064	10.3	30/1083	14.4	0.76 (0.43-1.32)		2.3 (1.4)	
>0.811 ≤0.987	25/1117	11.4	38/1142	17.4	0.59 (0.36-0.98)		2.7 (1.8)	
≤0.811	85/1123	38.5	84/1080	39.5	0.99 (0.73-1.33)		6.2 (4.0)	
**Overall**	**131/3304**	**20.4**	**152/3305**	**23.7**	**0.84 (0.67-1.07)**			
**Hospitalization for any cause (first and all subsequent events)**						0.01[†]		0.22
>0.987	401	196	453	215	0.99 (0.83-1.20)		30.2 (9.4)	
>0.811 ≤0.987	407	185	579	263	0.67 (0.56-0.80)		36.9 (11.4)	
≤0.811	803	356	863	397	0.90 (0.77-1.05)		55.7 (17.2)	
**Overall**	**1611**	**248**	**1895**	**292**	**0.86 (0.78-0.95)**			
**Death from any cause**						0.08		0.34
>0.987	22/1064	10.8	26/1083	12.4	0.91 (0.52-1.61)		1.6 (1.2)	
>0.811 ≤0.987	28/1117	12.7	47/1142	21.3	0.54 (0.34-0.86)		2.8 (2.1)	
≤0.811	98/1123	43.5	94/1080	43.2	1.02 (0.76-1.35)		5.7 (4.2)	
**Overall**	**148/3304**	**22.8**	**167/3305**	**25.8**	**0.87 (0.70-1.08)**			
**OTHER SECONDARY OUTCOMES**								
**Any kidney disease progression**						0.70		0.89
>0.987	118/1064	59.3	161/1083	79.3	0.77 (0.61-0.98)		23.1 (3.8)	
>0.811 ≤0.987	137/1117	64.5	183/1142	86.4	0.68 (0.54-0.85)		25.2 (4.2)	
≤0.811	129/1123	58.9	160/1080	76.9	0.68 (0.54-0.86)		22.4 (3.7)	
**Overall**	**384/3304**	**60.9**	**504/3305**	**80.9**	**0.71 (0.62-0.81)**			
**Death from cardiovascular causes**						0.89		0.56
>0.987	8/1064	3.9	10/1083	4.8	0.85 (0.34-2.16)		0.7 (0.7)	
>0.811 ≤0.987	15/1117	6.8	14/1142	6.3	0.98 (0.47-2.02)		1.0 (1.0)	
≤0.811	36/1123	16.0	45/1080	20.7	0.79 (0.51-1.23)		3.2 (3.1)	
**Overall**	**59/3304**	**9.1**	**69/3305**	**10.6**	**0.84 (0.60-1.19)**			
**ESKD or death from cardiovascular causes[‡]**						0.88		0.13
>0.987	34/1064	16.8	50/1083	24.0	0.72 (0.46-1.11)		6.5 (1.8)	
>0.811 ≤0.987	57/1117	26.3	68/1142	31.3	0.78 (0.55-1.11)		8.5 (2.4)	
≤0.811	72/1123	32.3	99/1080	46.4	0.69 (0.51-0.94)		12.6 (3.5)	
**Overall**	**163/3304**	**25.4**	**217/3305**	**34.0**	**0.73 (0.59-0.89)**			

The EQ-5D index value is a weighted index of the 5 EQ-5D domain scores (mobility, self-care, usual activities, pain/discomfort and anxiety/depression) derived using established methodology (see Supplementary Methods); lower values indicate poorer quality of life. The p values shown are standard tests for heterogeneity or trend across categories of EQ-5D index for the relative and estimated absolute effects; respectively. Hazard ratios are not presented for outcomes with fewer than 10 events. * Absolute events avoided per 1000 patients treated with empagliflozin for 1 year (SE) were estimated by applying the overall hazard ratio (or 95% CI) to the subgroup-specific event rate per 1000 patient-years in the placebo group. † If subgroup-specific hazard ratios (or CIs) were used to estimate absolute effects on all-cause hospitalization by health-related quality of life, based on P for heterogeneity = 0.01 for relative effects; estimated absolute events avoided (SE) would be 1.3 (20.1), 87.0 (15.9) and 38.8 (28.4) rather than 30.2 (9.4), 36.9 (11.4) and 55.7 (17.2). ‡ ESKD: End-Stage Kidney Disease, defined as start of maintenance dialysis or receipt of a kidney transplant.

Supplementary Table 11: Safety outcomes by predicted risk of hospitalization

Predicted risk of hospitalization (%)	Empagliflozin		Placebo		Relative effects		Estimated absolute effects*	
	n/N	Rate per 1000 patient-years	n/N	Rate per 1000 patient-years	Hazard Ratio (95% CI)	P_{het}	Events caused per 1000 patient-years (SE)	P_{trend}
**Serious urinary tract infection**						0.99		0.76
≤20%	7/983	3.7	8/1005	4.2			-0.3 (0.8)	
>20% ≤35%	13/1245	5.4	15/1259	6.0			-0.4 (1.1)	
>35% ≤45%	12/487	12.4	13/481	13.7			-0.9 (2.5)	
>45%	20/589	17.0	18/560	16.3			-1.0 (3.0)	
**Overall**	**52/3304**	**8.1**	**54/3305**	**8.4**	**0.94 (0.64-1.37)**			
**Serious genital infection**						-		-
≤20%	0/983	0.0	0/1005	0.0			-	
>20% ≤35%	0/1245	0.0	0/1259	0.0			-	
>35% ≤45%	1/487	1.0	0/481	0.0			-	
>45%	0/589	0.0	1/560	0.9			-	
**Overall**	**1/3304**	**0.2**	**1/3305**	**0.2**	-			
**Serious hyperkalaemia**						0.59		0.27
≤20%	11/983	5.9	17/1005	9.1			-1.6 (1.1)	
>20% ≤35%	32/1245	13.3	41/1259	16.7			-2.9 (2.0)	
>35% ≤45%	21/487	21.8	16/481	17.0			-2.9 (2.0)	
>45%	28/589	24.3	35/560	32.6			-5.6 (3.8)	
**Overall**	**92/3304**	**14.4**	**109/3305**	**17.2**	**0.83 (0.63-1.09)**			
**Serious acute kidney injury**						0.28		0.01
≤20%	7/983	3.7	13/1005	6.9			-1.5 (0.7)	
>20% ≤35%	37/1245	15.4	41/1259	16.6			-3.7 (1.7)	
>35% ≤45%	14/487	14.4	28/481	29.9			-6.6 (3.0)	
>45%	49/589	42.8	53/560	48.6			-10.7 (4.9)	
**Overall**	**107/3304**	**16.7**	**135/3305**	**21.1**	**0.78 (0.60-1.00)**			
**Serious dehydration**						0.84		-
≤20%	0/983	0.0	0/1005	0.0			0.0 (0.0)	
>20% ≤35%	12/1245	4.9	8/1259	3.2			0.8 (1.1)	
>35% ≤45%	5/487	5.1	6/481	6.3			1.6 (2.1)	
>45%	13/589	11.0	10/560	9.0			2.3 (3.1)	
**Overall**	**30/3304**	**4.6**	**24/3305**	**3.7**	**1.25 (0.73-2.14)**			
**Liver injury**						0.67		0.88
≤20%	2/983	1.1	2/1005	1.1			0.1 (0.5)	
>20% ≤35%	6/1245	2.5	3/1259	1.2			0.1 (0.5)	
>35% ≤45%	2/487	2.0	3/481	3.1			0.3 (1.4)	
>45%	3/589	2.5	4/560	3.6			0.3 (1.6)	
**Overall**	**13/3304**	**2.0**	**12/3305**	**1.9**	**1.09 (0.50-2.38)**			
**Ketoacidosis**						-		-
≤20%	0/983	0.0	0/1005	0.0			-	
>20% ≤35%	3/1245	1.2	0/1259	0.0			-	
>35% ≤45%	0/487	0.0	1/481	1.0			-	
>45%	3/589	2.5	0/560	0.0			-	
**Overall**	**6/3304**	**0.9**	**1/3305**	**0.2**	**-**			
**Lower limb amputation**						0.40		0.93
≤20%	1/983	0.5	1/1005	0.5			0.2 (0.2)	
>20% ≤35%	7/1245	2.9	1/1259	0.4			0.2 (0.2)	
>35% ≤45%	8/487	8.2	7/481	7.4			3.2 (3.1)	
>45%	12/589	10.2	10/560	9.0			3.9 (3.8)	
**Overall**	**28/3304**	**4.3**	**19/3305**	**2.9**	**1.43 (0.80-2.57)**			
**Bone fracture**						0.29		0.77
≤20%	23/983	12.4	28/1005	15.0			1.1 (2.0)	
>20% ≤35%	38/1245	15.8	34/1259	13.8			1.0 (1.9)	
>35% ≤45%	35/487	36.9	22/481	23.4			1.8 (3.2)	
>45%	37/589	32.2	39/560	36.0			2.7 (4.8)	
**Overall**	**133/3304**	**20.9**	**123/3305**	**19.3**	**1.08 (0.84-1.38)**			
**Severe hypoglycaemia†**						0.45		0.96
≤20%	4/983	2.1	2/1005	1.1			-0.0 (0.2)	
>20% ≤35%	21/1245	8.7	29/1259	11.7			-0.1 (1.9)	
>35% ≤45%	17/487	17.6	20/481	21.3			-0.1 (3.4)	
>45%	35/589	30.5	26/560	24.1			-0.1 (3.9)	
**Overall**	**77/3304**	**12.0**	**77/3305**	**12.1**	**<1.00 (0.73-1.37)**			
**Symptomatic dehydration‡**						0.995		0.59
≤20%	10/983	5.4	10/1005	5.3			0.5 (0.9)	
>20% ≤35%	30/1245	12.5	27/1259	10.9			1.1 (1.9)	
>35% ≤45%	14/487	14.6	12/481	12.7			1.3 (2.2)	
>45%	29/589	25.1	27/560	24.7			2.6 (4.3)	
**Overall**	**83/3304**	**13.0**	**76/3305**	**11.9**	**1.10 (0.81-1.51)**			

Predicted risk of hospitalization during follow-up (median 2 years) was derived from multivariable logistic regression models assessing the association of all potential predictor variables with recorded hospitalization (first event; Supplemental Methods). The p values shown are standard tests for heterogeneity or trend across categories of predicted risk of hospitalization for the relative and estimated absolute effects; respectively. * Absolute events avoided per 1000 patients treated with empagliflozin for 1 year (SE) were estimated by applying the overall hazard ratio (or 95% CI) to the subgroup-specific event rate per 1000 patient-years in the placebo group. Hazard ratios are not presented for outcomes with <10 events. † Defined as low blood sugar causing severe cognitive impairment which requires assistance from another person for recovery. ‡ Defined as whether or not a participant has experienced symptoms they attribute to dehydration, such as feeling faint or fainting.

Supplementary Table 12: Safety outcomes by multimorbidity

No. of conditions (excluding CKD)	Empagliflozin		Placebo		Relative effects		Estimated absolute effects*	
	n/N	Rate per 1000 patient-years	n/N	Rate per 1000 patient-years	Hazard Ratio (95% CI)	P_{het}	Events caused per 1000 patient-years (SE)	P_{trend}
**Serious urinary tract infection**						0.42		0.87
≤1	18/1924	4.9	23/1940	6.2			-0.4 (1.1)	
2	13/706	9.2	15/663	11.3			-0.7 (2.1)	
≥3	21/674	15.8	16/702	11.5			-0.7 (2.1)	
**Overall**	**52/3304**	**8.1**	**54/3305**	**8.4**	**0.94 (0.64-1.37)**			
**Serious genital infection**								-
≤1	0/1924	0.0	0/1940	0.0		-	-	
2	0/706	0.0	0/663	0.0			-	
≥3	1/674	0.7	1/702	0.7			-	
**Overall**	**1/3304**	**0.2**	**1/3305**	**0.2**	**-**			
**Serious hyperkalaemia**						0.61		0.66
≤1	50/1924	13.6	51/1940	13.9			-2.4 (1.6)	
2	22/706	15.8	30/663	23.2			-4.0 (2.7)	
≥3	20/674	15.2	28/702	20.2			-3.5 (2.4)	
**Overall**	**92/3304**	**14.4**	**109/3305**	**17.2**	**0.83 (0.63-1.09)**			
**Serious acute kidney injury**						0.64		0.15
≤1	35/1924	9.5	51/1940	13.8			-3.0 (1.4)	
2	35/706	25.1	36/663	27.5			-6.1 (2.8)	
≥3	37/674	28.3	48/702	34.7			-7.6 (3.5)	
**Overall**	**107/3304**	**16.7**	**135/3305**	**21.1**	**0.78 (0.60-1.00)**			
**Serious dehydration**						0.77		0.60
≤1	8/1924	2.2	8/1940	2.1			0.5 (0.7)	
2	9/706	6.3	5/663	3.7			0.9 (1.3)	
≥3	13/674	9.7	11/702	7.9			2.0 (2.7)	
**Overall**	**30/3304**	**4.6**	**24/3305**	**3.7**	**1.25 (0.73-2.14)**			
**Liver injury**						0.55		0.88
≤1	7/1924	1.9	4/1940	1.1			0.1 (0.5)	
2	3/706	2.1	3/663	2.2			0.2 (1.0)	
≥3	3/674	2.2	5/702	3.6			0.3 (1.5)	
**Overall**	**13/3304**	**2.0**	**12/3305**	**1.9**	**1.09 (0.50-2.38)**			
**Ketoacidosis**						-		-
≤1	1/1924	0.3	0/1940	0.0			-	
2	2/706	1.4	0/663	0.0			-	
≥3	3/674	2.2	1/702	0.7			-	
**Overall**	**6/3304**	**0.9**	**1/3305**	**0.2**	**-**			
**Lower limb amputation**						0.33		0.20
≤1	7/1924	1.9	2/1940	0.5			0.2 (0.2)	
2	11/706	7.8	6/663	4.5			1.9 (1.9)	
≥3	10/674	7.5	11/702	7.9			3.4 (3.4)	
**Overall**	**28/3304**	**4.3**	**19/3305**	**2.9**	**1.43 (0.80-2.57)**			
**Bone fracture**						0.09		0.77
≤1	68/1924	18.6	52/1940	14.1			1.1 (1.9)	
2	23/706	16.4	33/663	25.2			1.9 (3.4)	
≥3	42/674	32.4	38/702	27.7			2.1 (3.7)	
**Overall**	**133/3304**	**20.9**	**123/3305**	**19.3**	**1.08 (0.84-1.38)**			
**Severe hypoglycaemia†**						0.34		0.97
≤1	19/1924	5.1	13/1940	3.5			-0.0 (0.6)	
2	26/706	18.7	23/663	17.6			-0.1 (2.8)	
≥3	32/674	24.5	41/702	30.2			-0.1 (4.9)	
**Overall**	**77/3304**	**12.0**	**77/3305**	**12.1**	**<1.00 (0.73-1.37)**			
**Symptomatic dehydration‡**						0.74		0.73
≤1	31/1924	8.4	30/1940	8.1			0.8 (1.4)	
2	23/706	16.5	16/663	12.1			1.3 (2.1)	
≥3	29/674	22.2	30/702	21.8			2.3 (3.8)	
**Overall**	**83/3304**	**13.0**	**76/3305**	**11.9**	**1.10 (0.81-1.51)**			

Multimorbidity was determined based on the presence/absence of 8 patient-reported comorbidities at randomization (see Supplementary Table 5) excluding chronic kidney disease. The p values shown are standard tests for heterogeneity or trend across categories of multimorbidity for the relative and estimated absolute effects; respectively. * Absolute events avoided per 1000 patients treated with empagliflozin for 1 year (SE) were estimated by applying the overall hazard ratio (or 95% CI) to the subgroup-specific event rate per 1000 patient-years in the placebo group. Hazard ratios are not presented for outcomes with <10 events. † Defined as low blood sugar causing severe cognitive impairment which requires assistance from another person for recovery. ‡ Defined as whether or not a participant has experienced symptoms they attribute to dehydration, such as feeling faint or fainting.

Supplementary Table 13: Safety outcomes by concomitant medication count

No. of concomitant medications	Empagliflozin		Placebo		Relative effects		Estimated absolute effects*	
	n/N	Rate per 1000 patient-years	n/N	Rate per 1000 patient-years	Hazard Ratio (95% CI)	P_{het}	Events caused per 1000 patient-years (SE)	P_{trend}
**Serious urinary tract infection**						0.40		0.87
≤5	8/1128	3.6	12/1121	5.5			-0.3 (1.0)	
≥6 <9	14/1010	7.1	17/1004	8.7			-0.5 (1.6)	
≥9	30/1166	13.2	25/1180	10.8			-0.7 (2.0)	
**Overall**	**52/3304**	**8.1**	**54/3305**	**8.4**	**0.94 (0.64-1.37)**			
**Serious genital infection**						-		-
≤5	0/1128	0.0	0/1121	0.0			-	
≥6 <9	0/1010	0.0	1/1004	0.5			-	
≥9	1/1166	0.4	0/1180	0.0			-	
**Overall**	**1/3304**	**0.2**	**1/3305**	**0.2**	-			
**Serious hyperkalaemia**						0.97		0.64
≤5	24/1128	11.0	29/1121	13.5			-2.3 (1.6)	
≥6 <9	24/1010	12.2	30/1004	15.5			-2.7 (1.8)	
≥9	44/1166	19.6	50/1180	22.1			-3.8 (2.6)	
**Overall**	**92/3304**	**14.4**	**109/3305**	**17.2**	**0.83 (0.63-1.09)**			
**Serious acute kidney injury**						0.26		0.12
≤5	16/1128	7.3	23/1121	10.6			-2.3 (1.1)	
≥6 <9	25/1010	12.6	42/1004	21.7			-4.8 (2.2)	
≥9	66/1166	29.5	70/1180	30.6			-6.7 (3.1)	
**Overall**	**107/3304**	**16.7**	**135/3305**	**21.1**	**0.78 (0.60-1.00)**			
**Serious dehydration**						0.30		0.57
≤5	1/1128	0.5	3/1121	1.4			0.3 (0.5)	
≥6 <9	4/1010	2.0	5/1004	2.6			0.6 (0.9)	
≥9	25/1166	11.0	16/1180	6.9			1.7 (2.4)	
**Overall**	**30/3304**	**4.6**	**24/3305**	**3.7**	**1.25 (0.73-2.14)**			
**Liver injury**						0.22		0.97
≤5	6/1128	2.7	3/1121	1.4			0.1 (0.6)	
≥6 <9	4/1010	2.0	2/1004	1.0			0.1 (0.4)	
≥9	3/1166	1.3	7/1180	3.0			0.3 (1.3)	
**Overall**	**13/3304**	**2.0**	**12/3305**	**1.9**	**1.09 (0.50-2.38)**			
**Ketoacidosis**						-		-
≤5	2/1128	0.9	0/1121	0.0			-	
≥6 <9	3/1010	1.5	0/1004	0.0			-	
≥9	1/1166	0.4	1/1180	0.4			-	
**Overall**	**6/3304**	**0.9**	**1/3305**	**0.2**	**-**			
**Lower limb amputation**						0.96		0.22
≤5	2/1128	0.9	1/1121	0.5			0.2 (0.2)	
≥6 <9	10/1010	5.1	7/1004	3.6			1.5 (1.5)	
≥9	16/1166	7.0	11/1180	4.7			2.0 (2.0)	
**Overall**	**28/3304**	**4.3**	**19/3305**	**2.9**	**1.43 (0.80-2.57)**			
**Bone fracture**						0.29		0.75
≤5	36/1128	16.6	25/1121	11.6			0.9 (1.6)	
≥6 <9	42/1010	21.6	34/1004	17.5			1.3 (2.4)	
≥9	55/1166	24.5	64/1180	28.2			2.1 (3.8)	
**Overall**	**133/3304**	**20.9**	**123/3305**	**19.3**	**1.08 (0.84-1.38)**			
**Severe hypoglycaemia†**						0.50		0.98
≤5	8/1128	3.6	5/1121	2.3			-0.0 (0.4)	
≥6 <9	20/1010	10.2	16/1004	8.2			-0.0 (1.3)	
≥9	49/1166	21.8	56/1180	24.8			-0.1 (4.0)	
**Overall**	**77/3304**	**12.0**	**77/3305**	**12.1**	**<1.00 (0.73-1.37)**			
**Symptomatic dehydration‡**						0.60		0.80
≤5	13/1128	6.0	16/1121	7.4			0.8 (1.3)	
≥6 <9	18/1010	9.2	16/1004	8.2			0.9 (1.4)	
≥9	52/1166	23.2	44/1180	19.2			2.0 (3.4)	
**Overall**	**83/3304**	**13.0**	**76/3305**	**11.9**	**1.10 (0.81-1.51)**			

The p values shown are standard tests for heterogeneity or trend across categories of concomitant medications for the relative and estimated absolute effects; respectively. * Absolute events avoided per 1000 patients treated with empagliflozin for 1 year (SE) were estimated by applying the overall hazard ratio (or 95% CI) to the subgroup-specific event rate per 1000 patient-years in the placebo group. Hazard ratios are not presented for outcomes with <10 events. † Defined as low blood sugar causing severe cognitive impairment which requires assistance from another person for recovery. ‡ Defined as whether or not a participant has experienced symptoms they attribute to dehydration, such as feeling faint or fainting.

Supplementary Table 14: Safety outcomes by health-related quality of life (EQ-5D index value)

EQ-5D index value	Empagliflozin		Placebo		Relative effects		Estimated absolute effects*	
	n/N	Rate per 1000 patient-years	n/N	Rate per 1000 patient-years	Hazard Ratio (95% CI)	P_{het}	Events caused per 1000 patient-years (SE)	P_{trend}
**Serious urinary tract infection**						0.55		0.88
>0.987	8/1064	3.9	11/1083	5.3			-0.3 (1.0)	
>0.811 ≤0.987	17/1117	7.8	13/1142	5.9			-0.4 (1.1)	
≤0.811	27/1123	12.1	30/1080	14.0			-0.9 (2.5)	
**Overall**	**52/3304**	**8.1**	**54/3305**	**8.4**	**0.94 (0.64-1.37)**			
**Serious genital infection**						-		-
>0.987	0/1064	0.0	0/1083	0.0			-	
>0.811 ≤0.987	0/1117	0.0	0/1142	0.0			-	
≤0.811	1/1123	0.4	1/1080	0.5			-	
**Overall**	**1/3304**	**0.2**	**1/3305**	**0.2**	-			
**Serious hyperkalaemia**						0.87		0.81
>0.987	25/1064	12.4	33/1083	16.0			-2.8 (1.9)	
>0.811 ≤0.987	22/1117	10.1	28/1142	12.9			-2.2 (1.5)	
≤0.811	45/1123	20.4	48/1080	22.8			-3.9 (2.7)	
**Overall**	**92/3304**	**14.4**	**109/3305**	**17.2**	**0.83 (0.63-1.09)**			
**Serious acute kidney injury**						0.11		0.22
>0.987	26/1064	12.8	27/1083	13.0			-2.9 (1.3)	
>0.811 ≤0.987	23/1117	10.6	45/1142	20.7			-4.6 (2.1)	
≤0.811	58/1123	26.4	63/1080	29.6			-6.5 (3.0)	
**Overall**	**107/3304**	**16.7**	**135/3305**	**21.1**	**0.78 (0.60-1.00)**			
**Serious dehydration**						0.25		0.62
>0.987	4/1064	2.0	4/1083	1.9			0.5 (0.7)	
>0.811 ≤0.987	7/1117	3.2	10/1142	4.6			1.1 (1.6)	
≤0.811	19/1123	8.5	10/1080	4.6			1.2 (1.6)	
**Overall**	**30/3304**	**4.6**	**24/3305**	**3.7**	**1.25 (0.73-2.14)**			
**Liver injury**						0.41		0.89
>0.987	5/1064	2.5	2/1083	1.0			0.1 (0.4)	
>0.811 ≤0.987	3/1117	1.4	5/1142	2.3			0.2 (1.0)	
≤0.811	5/1123	2.2	5/1080	2.3			0.2 (1.0)	
**Overall**	**13/3304**	**2.0**	**12/3305**	**1.9**	**1.09 (0.50-2.38)**			
**Ketoacidosis**						-		-
>0.987	1/1064	0.5	1/1083	0.5			-	
>0.811 ≤0.987	1/1117	0.5	0/1142	0.0			-	
≤0.811	4/1123	1.8	0/1080	0.0			-	
**Overall**	**6/3304**	**0.9**	**1/3305**	**0.2**	**-**			
**Lower limb amputation**						0.17		0.28
>0.987	8/1064	3.9	1/1083	0.5			0.2 (0.2)	
>0.811 ≤0.987	6/1117	2.7	4/1142	1.8			0.8 (0.8)	
≤0.811	14/1123	6.3	14/1080	6.5			2.8 (2.8)	
**Overall**	**28/3304**	**4.3**	**19/3305**	**2.9**	**1.43 (0.80-2.57)**			
**Bone fracture**						0.39		0.79
>0.987	37/1064	18.4	27/1083	13.0			1.0 (1.8)	
>0.811 ≤0.987	37/1117	17.1	38/1142	17.5			1.3 (2.4)	
≤0.811	59/1123	27.0	58/1080	27.4			2.1 (3.7)	
**Overall**	**133/3304**	**20.9**	**123/3305**	**19.3**	**1.08 (0.84-1.38)**			
**Severe hypoglycaemia†**						0.20		0.98
>0.987	11/1064	5.4	6/1083	2.9			-0.0 (0.5)	
>0.811 ≤0.987	22/1117	10.2	17/1142	7.8			-0.0 (1.2)	
≤0.811	44/1123	19.9	54/1080	25.7			-0.1 (4.1)	
**Overall**	**77/3304**	**12.0**	**77/3305**	**12.1**	**<1.00 (0.73-1.37)**			
**Symptomatic dehydration‡**						0.82		0.58
>0.987	12/1064	5.9	9/1083	4.3			0.4 (0.8)	
>0.811 ≤0.987	25/1117	11.6	25/1142	11.5			1.2 (2.0)	
≤0.811	46/1123	20.9	42/1080	19.8			2.0 (3.5)	
**Overall**	**83/3304**	**13.0**	**76/3305**	**11.9**	**1.10 (0.81-1.51)**			

The EQ-5D index value is a weighted index of the 5 EQ-5D domain scores (mobility, self-care, usual activities, pain/discomfort and anxiety/depression) derived using established methodology (see Supplementary Methods); lower values indicate poorer quality of life. The p values shown are standard tests for heterogeneity or trend across categories of EQ-5D index for the relative and estimated absolute effects; respectively.
* Absolute events avoided per 1000 patients treated with empagliflozin for 1 year (SE) were estimated by applying the overall hazard ratio (or 95% CI) to the subgroup-specific event rate per 1000 patient-years in the placebo group. Hazard ratios are not presented for outcomes with <10 events.
† Defined as low blood sugar causing severe cognitive impairment which requires assistance from another person for recovery. ‡ Defined as whether or not a participant has experienced symptoms they attribute to dehydration, such as feeling faint or fainting.

Supplementary Figure 1: Performance of the final multivariable logistic regression model in predicting hospitalization and all-cause death

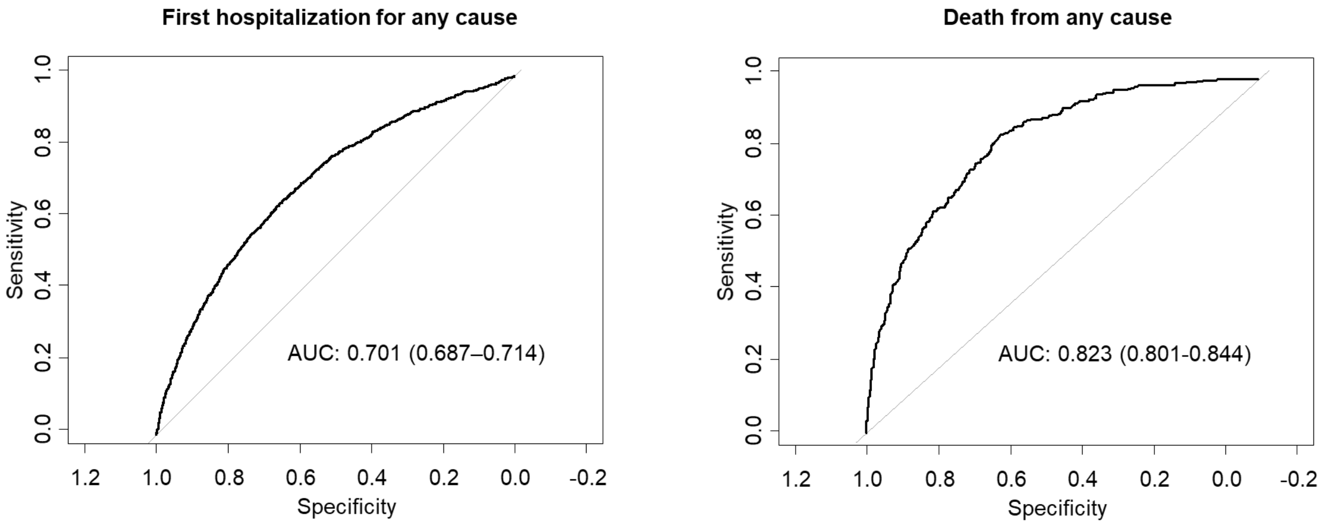

The final multivariable model is presented in Table 1 & Supplementary Table 3. AUC = area under the receiver operating characteristic curve.

Supplementary Figure 2: Associations between predicted risk of hospitalization and multimorbidity; polypharmacy; and health-related quality of life

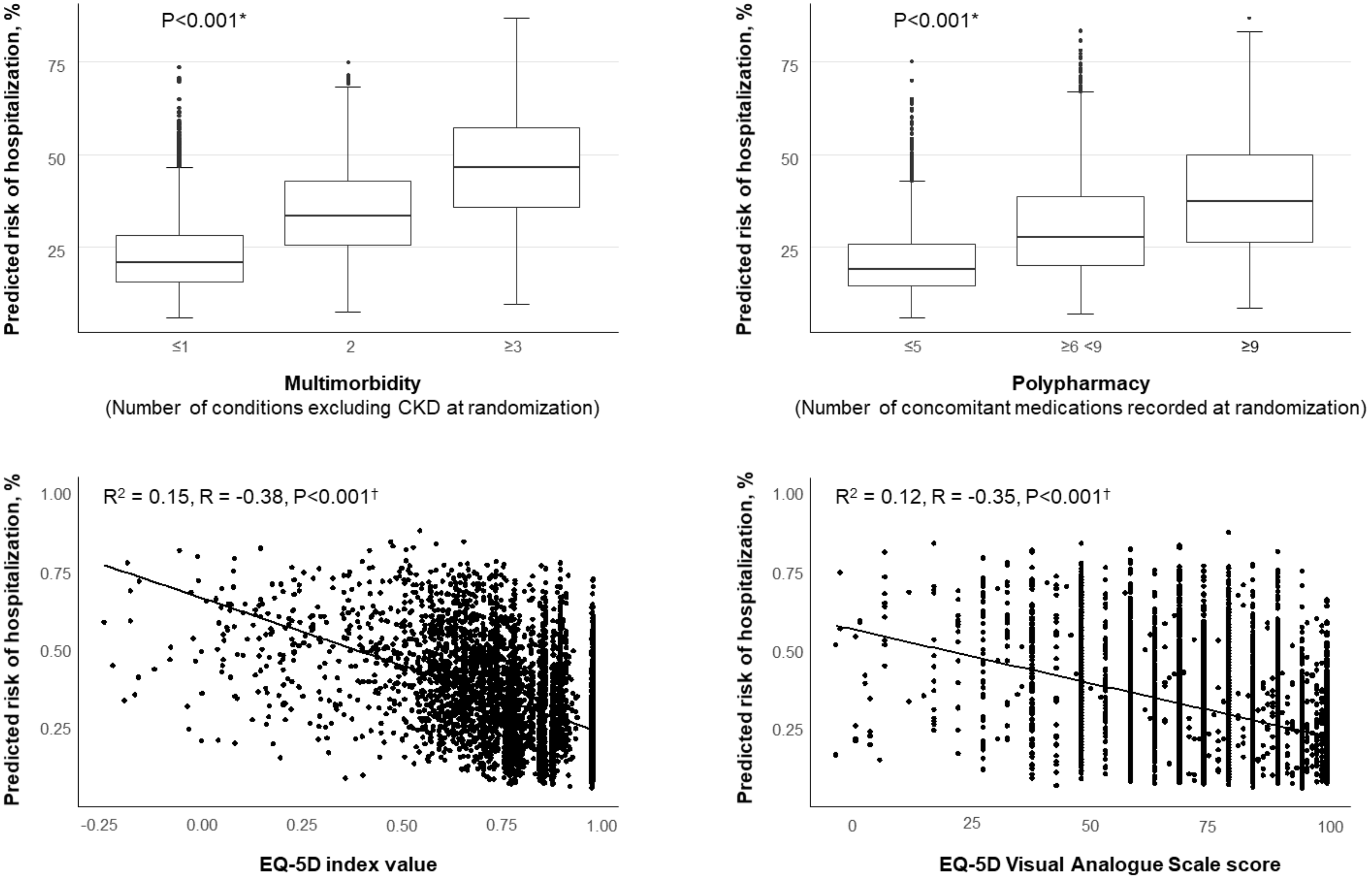

Predicted risk of hospitalization was derived from multivariable logistic regression models adjusted for age, sex and region assessing the association of all potential predictor variables with recorded hospitalization (first event; see Supplementary Methods). Multimorbidity was determined based on the presence/absence of eight patient-reported comorbidities at randomization (see Supplementary Table 5) excluding chronic kidney disease. The EQ-5D index value is a weighted index of the 5 EQ-5D domain scores (mobility, self-care, usual activities, pain/discomfort and anxiety/depression) derived using established methodology (see Supplementary Methods); lower values indicate poorer quality of life. The Visual Analogue Scale asks participants to rate their overall health on the day of randomization between zero and 100 reflecting the worst and best health imaginable, respectively. * P value = analysis of variance (ANOVA). † Spearman's rank-order correlation.

Supplementary Figure 3: Number of participants in the highest level of frailty (defined as predicted risk of hospitalization >45%) in EMPA-KIDNEY showing overlap with conventional definitions of multimorbidity and polypharmacy (which differ from Figure 1)

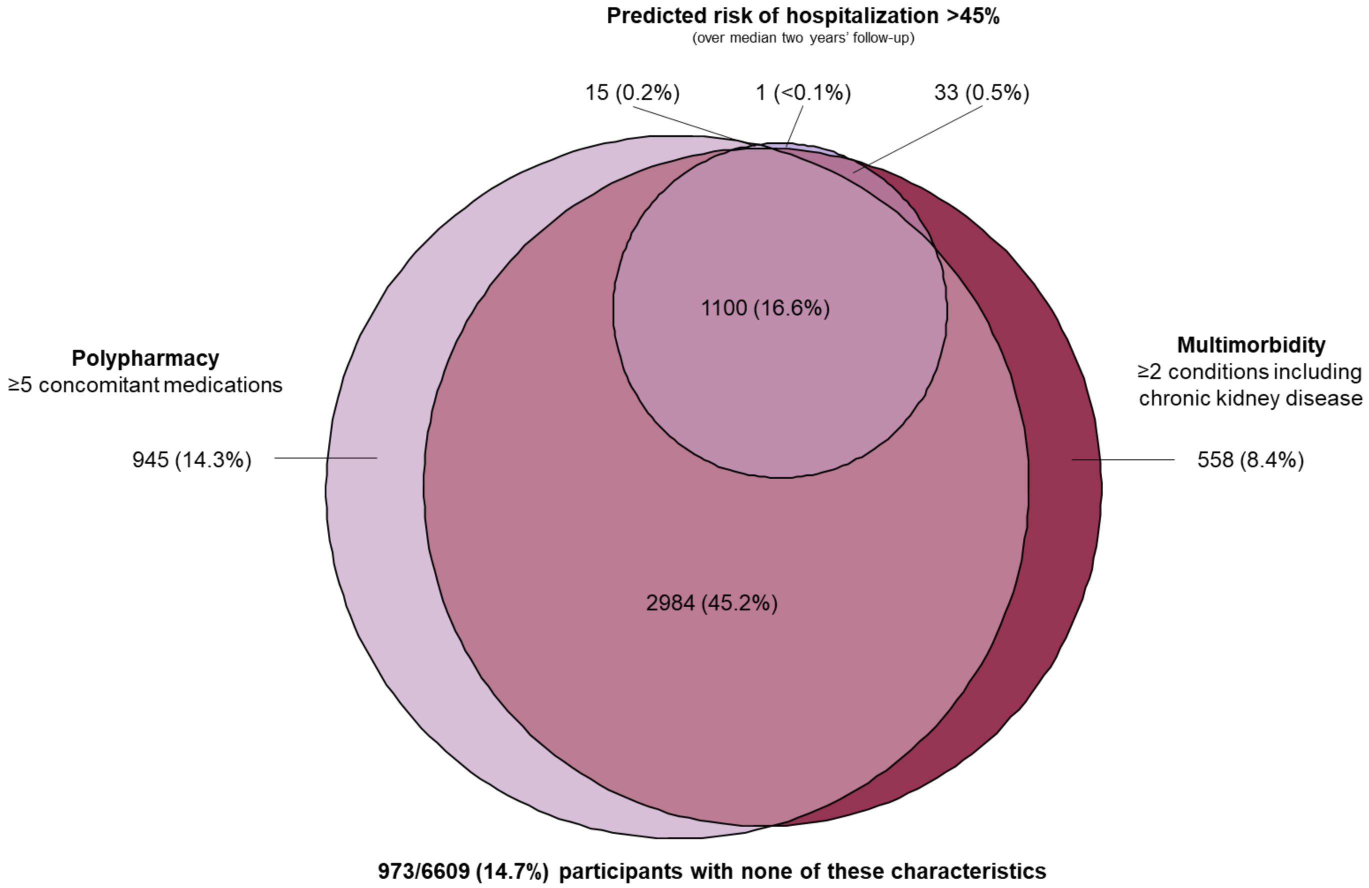

Supplementary Figure 4: Effects of empagliflozin versus placebo on recurrent all-cause hospitalization by frailty (based on predicted risk of hospitalization), multimorbidity, polypharmacy and health-related quality of life

PREDICTED RISK OF HOSPITALIZATION
(Over median 2 years' follow-up)

Predicted risk of hospitalization	Empagliflozin n	Empagliflozin Rate per 100 patient-years	Placebo n	Placebo Rate per 100 patient-years	Hazard Ratio (95% CI)	Estimated absolute events avoided per 100 patient-years (95% CI)
≤20%	186	9.9	221	11.6	0.86 (0.68-1.07)	2 (1-3)
>20% ≤35%	501	20.6	577	23.0	0.91 (0.77-1.06)	3 (1-5)
>35% ≤45%	327	33.2	395	41.0	0.79 (0.63-0.98)	6 (2-9)
>45%	597	50.2	702	62.7	0.78 (0.65-0.95)	9 (3-14)
**OVERALL**	**1611**	**24.8**	**1895**	**29.2**	**0.86 (0.78-0.95)**	

Heterogeneity p value = 0.63 | Trend p value <0.001

MULTIMORBIDITY

Number of conditions excluding CKD	Empagliflozin n	Empagliflozin Rate per 100 patient-years	Placebo n	Placebo Rate per 100 patient-years	Hazard Ratio (95% CI)	Estimated absolute events avoided per 100 patient-years (95% CI)
≤1	638	29.0	728	33.3	0.89 (0.77-1.03)	5 (2-8)
2	426	29.9	486	36.3	0.83 (0.68-1.01)	5 (2-8)
≥3	547	40.7	681	48.3	0.83 (0.69-1.00)	7 (3-11)
**OVERALL**	**1611**	**24.8**	**1895**	**29.2**	**0.86 (0.78-0.95)**	

Heterogeneity p value = 0.78 | Trend p value = 0.44

POLYPHARMACY

Number of concomitant medications	Empagliflozin n	Empagliflozin Rate per 100 patient-years	Placebo n	Placebo Rate per 100 patient-years	Hazard Ratio (95% CI)	Estimated absolute events avoided per 100 patient-years (95% CI)
≤5	353	16.0	335	15.3	1.06 (0.87-1.28)	2 (1-3)
≥6 <9	444	20.2	568	25.8	0.77 (0.65-0.92)	4 (1-6)
≥9	814	35.4	992	42.5	0.83 (0.71-0.96)	6 (2-10)
**OVERALL**	**1611**	**24.8**	**1895**	**29.2**	**0.86 (0.78-0.95)**	

Heterogeneity p value = 0.05 | Trend p value = 0.04

HEALTH-RELATED QUALITY OF LIFE

EQ-5D index value	Empagliflozin n	Empagliflozin Rate per 100 patient-years	Placebo n	Placebo Rate per 100 patient-years	Hazard Ratio (95% CI)	Estimated absolute events avoided per 100 patient-years (95% CI)
>0.987	401	19.6	453	21.5	0.99 (0.83-1.20)	3 (1-5)
>0.811 ≤0.987	407	18.5	579	26.3	0.67 (0.56-0.80)	4 (1-6)
≤0.811	803	35.6	863	39.7	0.90 (0.77-1.05)	6 (2-9)
**OVERALL**	**1611**	**24.8**	**1895**	**29.2**	**0.86 (0.78-0.95)**	

Heterogeneity p value = 0.01 | Trend p value = 0.22

The analysis of hospitalizations for any cause included the first and all subsequent events, n shown = total events; 1611 total hospitalizations occurred among 960 patients in the empagliflozin group, and 1895 total hospitalizations occurred among 1035 patients in the placebo group. Rates are presented per 100 patient-years to match previous reports. Predicted risk of hospitalization during follow-up (median two years) was derived from multivariable logistic regression models adjusted for age, sex and region assessing the association of all potential predictor variables with recorded hospitalization (first event; see Supplementary Methods). Multimorbidity was determined based on the presence/absence of 8 patient-reported comorbidities at randomization (see Supplementary Table 5) in addition to chronic kidney disease. The EQ-5D index value is a weighted index of the 5 EQ-5D domain scores (mobility, self-care, usual activities, pain/discomfort and anxiety/depression) derived using established methodology (see Supplementary Methods); lower values indicate poorer quality of life. Due to absence of any strong evidence of heterogeneity the presented characteristics, absolute events avoided per 100 patients treated with empagliflozin per one year (95% CI) were estimated by applying the overall hazard ratio (or 95% CI) to the event rate per 100 patient-years in the placebo group. If subgroup-specific hazard ratios (or CIs) were used to estimate absolute effects by health-related quality of life, based on P for heterogeneity = 0.01; estimated absolute events avoided (95% CI) would be 0.1 (-4, 4), 9 (6, 12) and 4 (-2, 9) rather than 3 (1, 5), 4 (1, 6) and 6 (2, 9).

Supplementary Figure 5: Effects of empagliflozin versus placebo on recurrent all-cause hospitalization by key pre-specified subgroups

Subgroup		Empagliflozin<br>Events per 100 patient-years	Placebo<br>Events per 100 patient-years	Hazard Ratio (95% CI)	
**Diabetes**	Yes	31.2	36.7	0.86	(0.75–0.98)
	No	19.1	22.6	0.86	(0.74–0.99)
**eGFR** (mL/min/1.73m^2)	<30	32.0	36.3	0.88	(0.75–1.03)
	≥30 <45	22.3	27.3	0.81	(0.70–0.94)
	≥45	18.3	21.3	0.91	(0.72–1.15)
**UACR** (mg/g)	<30	24.7	30.8	0.80	(0.65–0.99)
	≥30 ≤300	24.6	30.5	0.83	(0.69–0.99)
	>300	24.9	27.9	0.89	(0.78–1.02)
**All participants**		**24.8**	**29.2**	**0.86**	**(0.78–0.95)**

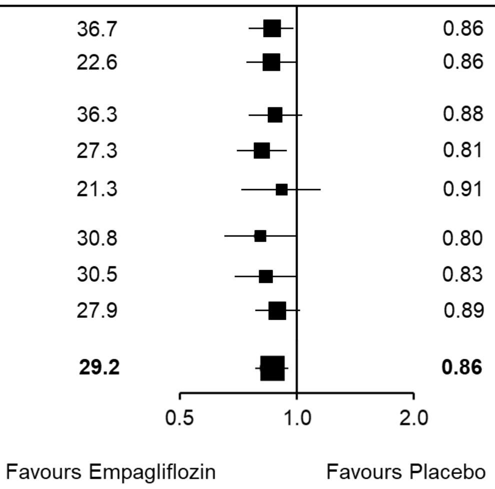

The analysis of hospitalizations for any cause included the first and all subsequent events; 1611 total hospitalizations occurred among 960 patients in the empagliflozin group, and 1895 total hospitalizations occurred among 1035 patients in the placebo group. Abbreviations: eGFR = estimated glomerular filtration rate; UAR = urinary albumin-to-creatinine ratio.

Supplementary Figure 6: Effects of empagliflozin versus placebo on weight and blood pressure by predicted risk of hospitalization

WEIGHT (kg)

	Baseline Mean (SE)	Difference (95% CI)	P_{het}
**Risk of hospitalization**			**0.88**
≤20%	80.2 (0.5)	-0.9 (-1.5, -0.4)	
>20% ≤35%	82.3 (0.4)	-1.0 (-1.4, -0.5)	
>35% ≤45%	87.1 (0.7)	-1.1 (-1.8, -0.3)	
>45%	91.5 (0.7)	-0.7 (-1.4, 0.0)	
**Overall**	**84.0 (0.3)**	**-0.9 (-1.2, -0.6)**	

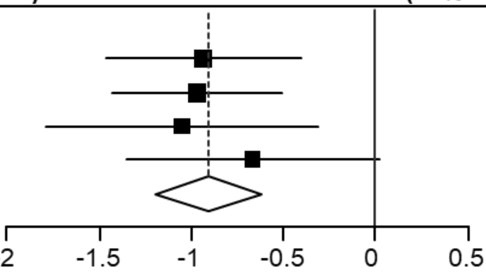

SYSTOLIC BLOOD PRESSURE (mmHg)

	Baseline Mean (SE)	Difference (95% CI)	P_{het}
**Risk of hospitalization**			**0.66**
≤20%	131.6 (0.3)	-2.1 (-3.4, -0.8)	
>20% ≤35%	138.4 (0.4)	-2.6 (-3.7, -1.5)	
>35% ≤45%	140.5 (0.6)	-2.6 (-4.4, -0.9)	
>45%	137.7 (0.6)	-3.4 (-5.0, -1.8)	
**Overall**	**136.5 (0.2)**	**-2.6 (-3.3, -1.9)**	

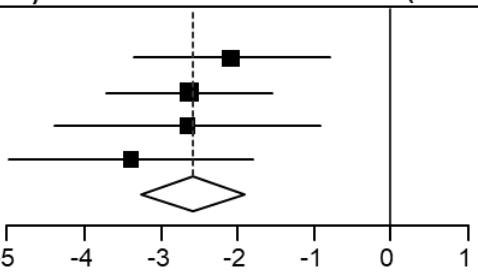

DIASTOLIC BLOOD PRESSURE (mmHg)

	Baseline Mean (SE)	Difference (95% CI)	P_{het}
**Risk of hospitalization**			**0.80**
≤20%	81.7 (0.2)	-0.4 (-1.2, 0.3)	
>20% ≤35%	78.8 (0.2)	-0.3 (-0.9, 0.3)	
>35% ≤45%	74.9 (0.4)	-0.7 (-1.7, 0.4)	
>45%	72.7 (0.4)	-0.8 (-1.8, 0.1)	
**Overall**	**78.1 (0.1)**	**-0.5 (-0.9, -0.1)**	

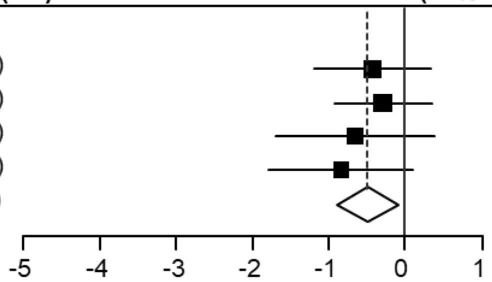

Predicted risk of hospitalization was derived from multivariable logistic regression models adjusted for age, sex and region assessing the association of all potential predictor variables with recorded hospitalization (first event; see Supplementary Methods). Study-average differences are adjusted for baseline values of the dependent variable (in continuous form) and for any differences in key baseline characteristics (categories of age, sex, diabetes, estimated glomerular filtration rate, urinary albumin-to-creatinine ratio and region) between treatment groups and weighted in proportion to the amount of time between follow-up visits. Each analysis includes all individuals with measurement of the outcome variable at baseline and at least once during follow-up.
